# Multistability maintains redox homeostasis in human cells

**DOI:** 10.15252/msb.202110480

**Published:** 2021-10-06

**Authors:** Jo‐Hsi Huang, Hannah KC Co, Yi‐Chen Lee, Chia‐Chou Wu, Sheng‐hong Chen

**Affiliations:** ^1^ Department of Chemical and Systems Biology Stanford University School of Medicine Stanford CA USA; ^2^ Institute of Molecular Biology Academia Sinica Taipei Taiwan; ^3^ Molecular and Cell Biology Taiwan International Graduate Program Academia Sinica and Graduate Institute of Life Science National Defense Medical Center Taipei Taiwan; ^4^ Genome and Systems Biology Degree Program Academia Sinica and National Taiwan University Taipei Taiwan

**Keywords:** bistability, glucose deprivation, redox homeostasis, Metabolism, Signal Transduction

## Abstract

Cells metabolize nutrients through a complex metabolic and signaling network that governs redox homeostasis. At the core of this, redox regulatory network is a mutually inhibitory relationship between reduced glutathione and reactive oxygen species (ROS)—two opposing metabolites that are linked to upstream nutrient metabolic pathways (glucose, cysteine, and glutamine) and downstream feedback loops of signaling pathways (calcium and NADPH oxidase). We developed a nutrient‐redox model of human cells to understand system‐level properties of this network. Combining *in silico* modeling and ROS measurements in individual cells, we show that ROS dynamics follow a switch‐like, all‐or‐none response upon glucose deprivation at a threshold that is approximately two orders of magnitude lower than its physiological concentration. We also confirm that this ROS switch can be irreversible and exhibits hysteresis, a hallmark of bistability. Our findings evidence that bistability modulates redox homeostasis in human cells and provide a general framework for quantitative investigations of redox regulation in humans.

## Graphical abstract

*In‐silico* modeling of a nutrient‐redox network and single‐cell measurements of ROS demonstrate that ROS dynamics follow a bistable response to glucose deprivation in human cells.

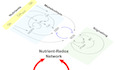

## Introduction

Cells gain energy, mass, and reducing potential by metabolizing environmental nutrients. During these metabolic processes, reactive oxygen species (ROS) can be generated as a signaling molecule. At physiological concentrations, ROS regulate various cellular and physiological functions such as mitogen signaling and the inflammatory response (D'Autreaux & Toledano, 2007; Finkel, 2011; Ayala *et al*, 2014; Zhang *et al*, 2016; Katikaneni *et al*, 2020; Sies & Jones, 2020). However, if they escape regulatory control, ROS can also cause oxidative stress, cell death, and pathological abnormalities such as neurodegeneration and abnormal aging (Sastre *et al*, 2003; Ayala *et al*, 2014; Yang & Stockwell, 2016; Angelova & Abramov, 2018; Katikaneni *et al*, 2020; Sies & Jones, 2020). Therefore, ROS must be tightly controlled by maintaining proper levels of reducing molecules to ensure cell survival and physiological homeostasis.

NADPH and reduced glutathione (GSH) are two major reducing molecules that dampen ROS in cells. NADPH is the cofactor for GSH regeneration, peroxiredoxin recycling (Hanschmann *et al*, 2013), and cystine reduction into bioactive cysteine (a precursor for *de novo* GSH synthesis) (Pader *et al*, 2014). Likewise, GSH buffers protein oxidation (Dalle‐Donne *et al*, 2009), recycles glutaredoxins (Fernandes & Holmgren, 2004), and powers glutathione peroxidases such as GPX4 (an enzyme that prevents ferroptosis by removing lipid hydroperoxides) (Yang *et al*, 2014).

NADPH and GSH production require extracellular nutrients, including glucose, glutamine, and cystine. Glucose fluxes through the oxidative branch of the pentose phosphate pathway (oxPPP) and regenerates NADPH from NADP^+^. Downstream metabolites of both glucose and glutamine can regenerate NADPH through enzymes (ME1 and IDH1) associated with the TCA cycle. Together, the TCA cycle and oxPPP account for nearly all cytosolic NADPH production (Chen *et al*, 2019). For GSH synthesis, glutamine and cystine are metabolized into glutamate and cysteine (Cys), respectively, the two substrates necessary for the rate‐limiting step of *de novo* GSH synthesis, namely glutamate‐cysteine ligation that is catalyzed by glutamate‐cysteine ligase (GCL) (Grant *et al*, 1997; Huang *et al*, 2000). Accordingly, depriving cells of glucose (Lee *et al*, 1998; Jelluma *et al*, 2006; Graham *et al*, 2012; Goji *et al*, 2017; Joly *et al*, 2020; Liu *et al*, 2020), glutamine (Cetinbas *et al*, 2016; Schulte *et al*, 2018; Jin *et al*, 2020), or cystine (Murphy *et al*, 1989; Gao *et al*, 2015; Tang *et al*, 2016; Yu & Long, 2016; Poursaitidis *et al*, 2017) greatly diminishes NADPH and GSH levels, leading to oxidative stress and triggering cell death.

Although glucose, glutamine, and cystine all contribute to cellular reducing potential, their metabolism also creates an oxidative burden. Downstream metabolites of glucose and glutamine feed into the TCA cycle and drive oxidative phosphorylation (OXPHOS), leading to ROS production through electron leakage from the electron transport chain (ETC). Reduction of intracellular cystine consumes NADPH (Pader *et al*, 2014). When NADPH is limited due to starvation (Goji *et al*, 2017; Joly *et al*, 2020; Liu *et al*, 2020), cystine switches from being a precursor of the GSH antioxidant into an oxidative toxin by further depleting NADPH. Moreover, cystine uptake is coupled in a 1:1 ratio to glutamate export through the cystine‐glutamate antiporter system xCT (or Xc^−^) that is encoded by the *SLC7A11* gene (Bannai, 1986; Koppula *et al*, 2018). Excessive extracellular cystine drives cystine‐glutamate exchange, which competes for intracellular glutamate with the TCA cycle for energy (Muir *et al*, 2017; Sayin *et al*, 2017) and NADPH (Goji *et al*, 2017) production.

This highly complex cost–benefit relationship between redox activity and nutrients is exacerbated by signaling pathways, including calcium and NADPH oxidase (NOX)‐mediated tyrosine kinase (TK) signaling (Graham *et al*, 2012), which feed back to ROS production. Therefore, it is a daunting challenge to understand how extracellular nutrients determine cellular redox state and how redox homeostasis can be maintained in the presence of nutrient fluctuations. We reasoned that elucidating systems and quantitative features contributing to this metabolism and signaling network is fundamental to understanding how cells respond to nutrient perturbations.

In recent years, several mathematical models have been developed to help understand various aspects of redox metabolism and ROS‐mediated signaling. These include nutrient transport and its links to intracellular glutathione metabolism (Reed *et al*, 2008; Geenen *et al*, 2012, 2013), ROS‐mediated signaling at the plasma membrane and across mitochondrial networks (Zhou *et al*, 2010; Grecco *et al*, 2011; Nivala *et al*, 2011; Travasso *et al*, 2017), as well as the ferroptosis signaling cascade (Konstorum *et al*, 2020). However, none of these models integrate nutrient metabolism and ROS‐mediated signaling as a redox system, nor did the respective studies investigate how deprivation of redox‐modulating nutrients—glucose, cystine, and glutamine, particularly via the cystine‐glutamate antiporter SLC7A11 (Goji *et al*, 2017; Joly *et al*, 2020; Liu *et al*, 2020)—may lead to elevated ROS and, consequently, cell death.

High glucose‐consuming organ systems, such as heart and brain (Hawkins *et al*, 1992), as well as cancer cells, can be predisposed to oxidative stress under glucose restriction. Although this redox imbalance causes pathological conditions in humans (e.g., hypoglycemia‐induced brain failure), glucose deprivation can be harnessed to target metabolic vulnerabilities in cancer cells. Acute and stringent glucose deprivation was shown to trigger oxidative cell death of multidrug‐resistant cancers (Lee *et al*, 1997, 1998). Subsequently, the molecular mechanisms underlying glucose deprivation‐induced oxidative cell death were further delineated in terms of contributory metabolic and signaling pathways, including nutrient dependency (Lee *et al*, 1998; Goji *et al*, 2017; Koppula *et al*, 2017; Shin *et al*, 2017), metabolic adaptation via AMPK signaling (Jeon *et al*, 2012), metabolic flexibility mediated by the antiporter SLC7A11 (Koppula *et al*, 2017; Shin *et al*, 2017; Liu *et al*, 2020), and signaling feedback to ROS (Graham *et al*, 2012; Lee *et al*, 2018), together highlighting potential therapeutic innovations (Joly *et al*, 2020; Liu *et al*, 2020).

**Figure 1:**
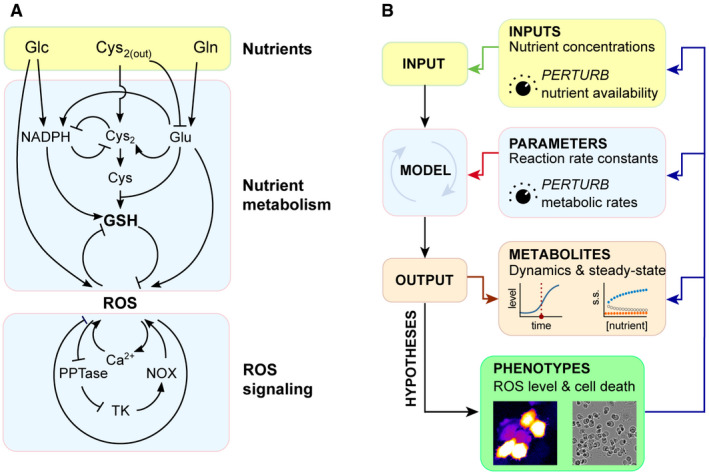
A nutrient‐redox model for human cells The network of nutrient metabolism and ROS signaling for building the nutrient‐redox model.Computing redox dynamics and steady state (OUTPUT) with the nutrient‐redox model by perturbing extracellular nutrient concentrations (INPUT) and metabolic parameters (MODEL). The hypotheses generated from the model were then tested experimentally using ROS imaging and cell death quantification (PHENOTYPES) that, in turn, feed back to all of the *in silico*‐modeled elements.

To integrate these mechanistic insights for a predictive model, we synthesize an inter‐connected metabolic and signaling network to build the first nutrient‐redox model, linking environmental nutrient availability to cellular redox state, as well as the feedback signaling, between glutathione and ROS (Fig 1A). Through this model, we recapitulate the redox dynamics of human cells, highlighting key mechanisms regulating redox imbalance during nutrient deprivation, either individually or in concert. At the systems level of the nutrient‐redox network, we investigate whether interlinked feedback loops give rise to bistability. Our simulations and experimental results show that ROS dynamics follow a switch‐like and all‐or‐none response upon glucose starvation at a threshold that is approximately two orders of magnitude lower than its physiological concentration. This feature of ROS bistability is further evidenced by its irreversibility and hysteresis. Our study provides a mathematical framework for investigating the human redox system and implicates bistability as a key mechanism responsible for redox homeostasis.

## Results

### A nutrient‐redox model of human cells

To investigate how nutrient availability determines the redox state of human cells, we built a nutrient‐redox model describing a redox regulatory network of human cells (Fig 1A and Materials and Methods). This redox regulatory network centers on the mutual inhibitory relationship between ROS and GSH, and it extends to the upstream nutrient metabolism and downstream signaling pathways that regulate the production and consumption of ROS and GSH (Fig 1A). The cellular redox state is modeled as a steady‐state balance between ROS and GSH. In striving for a more realistic recapitulation of cellular redox state, we constrained our model with experimentally measured metabolite and protein concentrations (first panel, Fig 1B and Appendix Table S1), as well as metabolic fluxes (second panel, Fig 1B and Appendix Table S4). Our model parameterizes extracellular nutrient concentrations (glucose, cystine, and glutamine) and expression of their transporters (e.g., SLC7A11 for cystine import) in order to simulate dynamic transitions and steady‐state changes in redox state under differential uptake of extracellular nutrients (third panel, Fig 1B). Moreover, our approach allowed us to pinpoint key regulatory steps for redox balance under various conditions of nutrient transport including the coupled import/export of cystine/glutamate by SLC7A11. Models such as ours can recapitulate systems‐level behaviors, inspiring hypotheses for experimental testing (fourth panel, Fig 1B). The behavior of our model, i.e., ROS and GSH steady states, exhibits differential dependency on glucose and is contingent on the parameter of choice. Parameter settings in this study correspond to those of glucose‐addicted human cells (see Materials and Methods and Discussion).

### Metabolite dynamics and redox catastrophe after glucose deprivation

**Figure 2:**
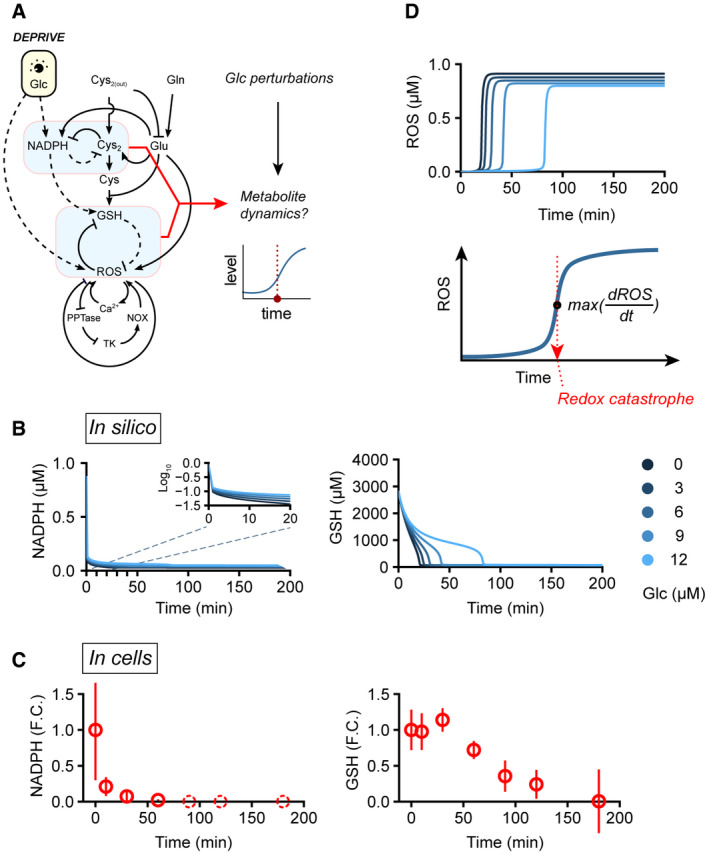
Redox dynamics and redox catastrophe upon glucose deprivation AIllustration of our *in silico* simulation strategy to establish the temporal dynamics of metabolites. Glucose perturbations (changed from 10 mM to 0–12 µM) were applied to the nutrient‐redox model, and then, the temporal dynamics of NADPH, cystine, GSH, and ROS were recorded to investigate changes in cellular redox state.B, CSimulated (*in silico*, (B)) and experimentally measured (in cells, (C)) NADPH and GSH dynamics upon glucose deprivation. Solid blue lines: simulated dynamics; solid red circles: mean of measured metabolite levels; red error bars: standard deviation of measured metabolite levels; dashed red circles: not detected in experiments. Experimental measurements were adopted from (Joly *et al*, 2020).DUpper panel: simulated ROS dynamics; lower panel: illustration of the occurrence of redox catastrophe, as defined by the time of maximum ROS rate of increase after glucose deprivation.

To determine how cells respond to low‐glucose conditions, we simulated dynamic changes of redox‐regulating metabolites (NADPH and GSH) and ROS after switching to low‐glucose concentrations (0–12 µM) (Fig 2A). In our simulations, NADPH was instantly depleted in response to low levels of glucose (slope ˜ 90% within 10 min; left panel, Fig 2B). Such instantaneous depletion could reflect our assumptions of rapid glucose breakdown and lack of glycogen storage in the model. In contrast, GSH exhibited delayed biphasic depletion, whereby an initially slow hyperbolic decline was followed by a second phase of rapid collapse (right panel, Fig 2B). Moreover, the duration of the first phase of GSH decline is modulated by glucose concentration in the low µM range (right panel, Fig 2B). Thus, depending on the concentration of glucose, the time difference between the 90% declines of NADPH and GSH can range from 20 min (0 µM) to 80 min (12 µM). In a glucose‐addicted cell line (T98), the experimentally measured NADPH time series shows a sharp 80% decrease in NADPH concentration within 10 min of glucose deprivation and a 90% decrease within 30 min (left panel, Fig 2C) (Joly *et al*, 2020). In contrast, the measured GSH time series in the T98 cell line reveals a 30‐min time lag before GSH concentration gradually declines thereafter over the course of 90 min (right panel, Fig 2C).

Consistent with our simulation results, we observed differing timescales for NADPH and GSH decline upon glucose deprivation, operating at fast and slow rates, respectively. Nevertheless, both NADPH and GSH in T98 cells exhibited delayed decline after glucose deprivation compared to simulations. In particular, GSH showed no sign of decline during the first 30 min of glucose deprivation (right panel, Fig 2C). These delays may be caused by potential compensatory mechanisms (e.g., glycogen storage) in T98 cells that sustain NADPH and GSH production upon glucose withdrawal. Alternatively, since our model uses metabolic rate constants approximated from multiple cell lines, it is possible that selection of parameters tailored to the T98 cell line would capture more precisely its redox dynamics upon glucose starvation.

**Figure EV1:**
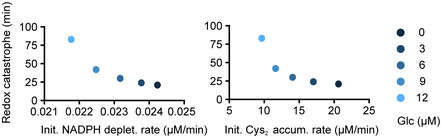
Rates of NADPH depletion and cystine accumulation determine the timing of redox catastrophe Time to redox catastrophe as functions of the NADPH depletion rate (left panel) or the cystine accumulation rate (right) after changing from maximal glucose (10,000 μM) to low glucose (0–12 μM) *in silico*.

Interestingly, in our simulations this rapid decline in GSH is concomitant with a swift increase in ROS (upper panel, Fig 2D). We refer to this rapid transition in redox state (collapse in GSH and rise in ROS) as a cellular redox catastrophe (lower panel, Fig 2D). It has been shown previously that NADPH depletion and cystine accumulation build up oxidative stress and are early determinants of cell death following glucose deprivation (Goji *et al*, 2017; Joly *et al*, 2020; Liu *et al*, 2020). In agreement with that notion, the initial rates of NADPH depletion and cystine accumulation in our simulations dictate the timing of redox catastrophe (Fig EV1). Thus, our model recapitulates redox dynamics in human cells undergoing glucose deprivation, providing a mathematical formulation for nutrient‐redox mapping in human cells.

### NADPH as a critical metabolite‐determining redox catastrophe

**Figure EV2:**
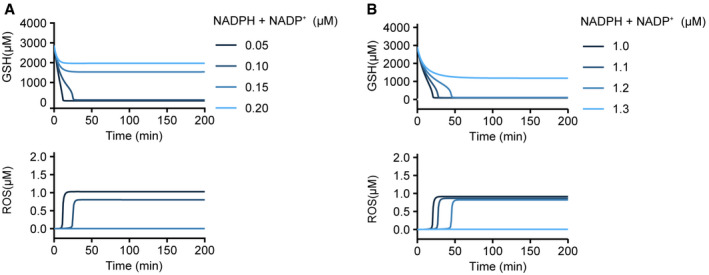
Total NADPH concentration determines redox dynamics and steady state Simulation of redox dynamics after a sudden decrease in total NADPH (NADPH + NADP^+^) concentration. The steady‐state metabolite concentrations (with 10,000 μM glucose and 1 μM total NADPH) were used as initial conditions for the simulation.Simulation of redox dynamics after glucose deprivation (from 10,000 to 0 μM) with varying total NADPH concentrations. Steady‐state metabolite concentrations with varying total NADPH concentrations before glucose deprivation were used as initial conditions for the simulation.

NADPH is an essential metabolite for both *de novo* synthesis and regeneration of GSH, so we explored if lowering the NADPH level alone (i.e., without glucose deprivation) could lead to similar redox catastrophe. We simulated redox dynamics upon decreasing total NADPH from 1 to 0.05–0.2 µM. We found that GSH was depleted when total NADPH ≤ 0.1 µM (upper panel, Fig EV2A), and this GSH depletion coincided with a swift increase in ROS (lower panel, Fig EV2A), which is reminiscent of the redox catastrophe observed after glucose deprivation. Given that NADPH can be a limiting factor for GSH production, we wondered if a larger pool of total NADPH could help prevent glucose deprivation‐induced redox catastrophe. To answer that question, we simulated glucose deprivation at varying concentrations of total NADPH and found that increasing total NADPH concentration from 1 to 1.3 µM was sufficient to prevent redox catastrophe after glucose deprivation (Fig EV2B), further underscoring the importance of NADPH for redox homeostasis. Together, these simulation results indicate that there is a threshold‐like NADPH level crucial for GSH maintenance. Glucose deprivation renders NADPH to fall below that threshold, thereby triggering GSH collapse and redox catastrophe.

### Feedback loop‐mediated redox bistability

**Figure 3:**
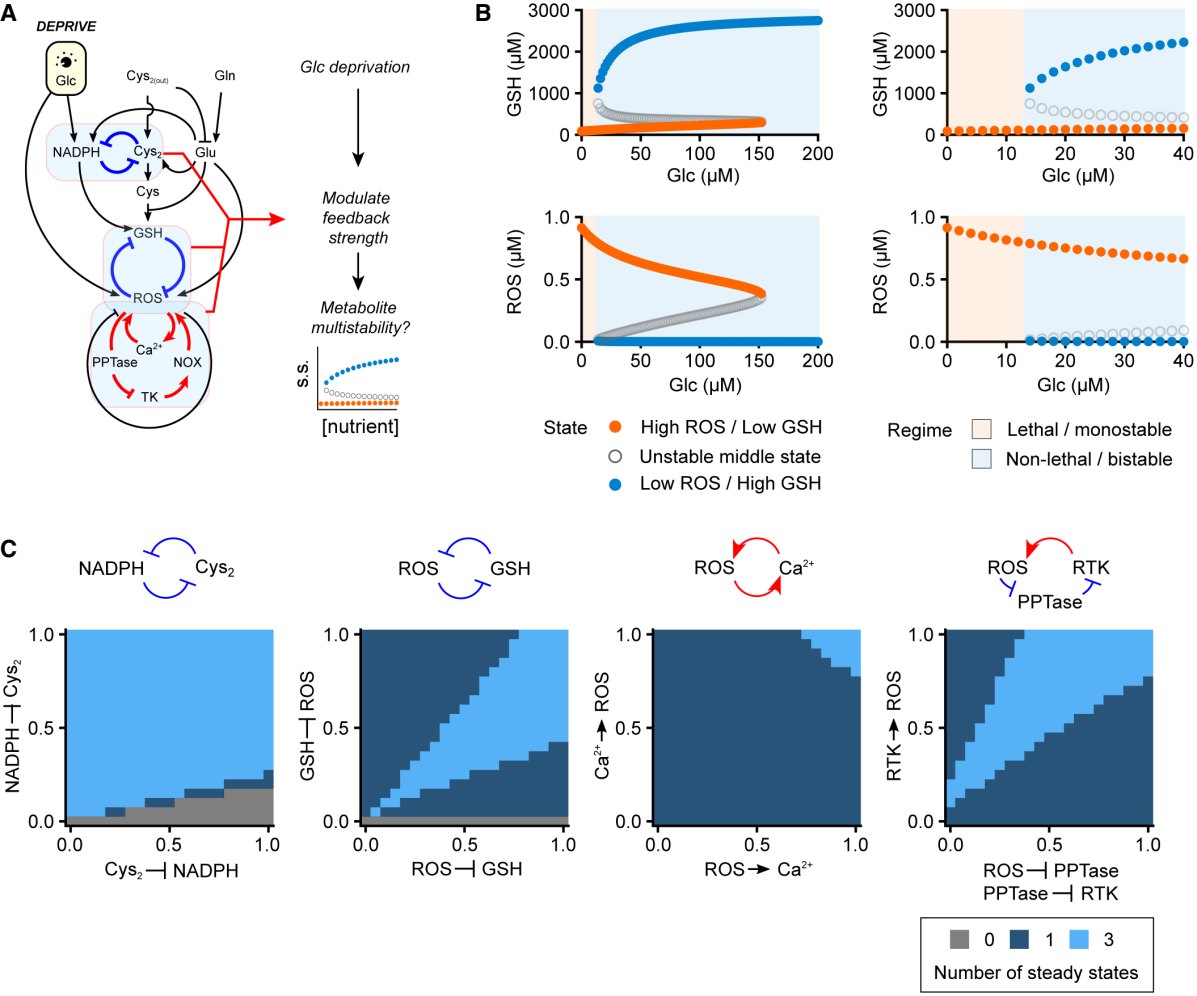
Bistability of the cellular redox state upon glucose deprivation Illustration of our *in silico* simulation strategy for revealing feedback effects on the redox steady state after glucose deprivation.Bifurcation diagrams of the redox state as a function of extracellular glucose concentration. Steady‐state concentrations of GSH (upper panel) and ROS (lower panel) were solved across a range of extracellular glucose concentrations. The high‐GSH/low‐ROS state (blue dots) disappears, with an unstable middle state (gray open circles) at a bifurcation point equivalent to ≈ 12 µM glucose. The panels at right are expanded views of the panels at left.Changes in redox bistability (light blue: bistable; mid‐blue: monostable; and dark blue: no steady state available) under perturbations of four different feedback loops.

Systems containing multiple feedback loops, such as the human redox system, can exhibit threshold‐like responses to input signals and multistability in steady states (Fig 3A). Therefore, we examined the steady‐state behavior of our modeled system with respect to varying concentrations of glucose and identified a bistable regime for redox state in the micromolar glucose range (12–160 µM), whereby a stable high‐GSH/low‐ROS state and a stable low GSH/high ROS state coexist (left panels for broader glucose range of 0–200 µM and right panels for focused glucose range of 0–40 µM, Fig 3B). This outcome indicates the existence of a glucose threshold (≈ 12 µM based on our current parameter settings) below which the redox state switches from being high‐GSH/low‐ROS to low GSH/high ROS, with this latter reflecting redox catastrophe. Next, we examined whether redox bistability depends on the strength of feedback loops in our nutrient‐redox model. Perturbations in all feedback loops, i.e., double‐negative feedback (cystine‐NADPH and ROS‐GSH) and positive feedback (ROS‐calcium and ROS‐PPTase‐TK) loops, abolished redox bistability (Fig 3C), consistent with previous reports on the involvement of these pathways for ROS regulation (Graham *et al*, 2012; Lee *et al*, 2018; Joly *et al*, 2020; Liu *et al*, 2020).

### Redox catastrophe as a ROS bistable switch at a low‐glucose threshold

**Figure 4:**
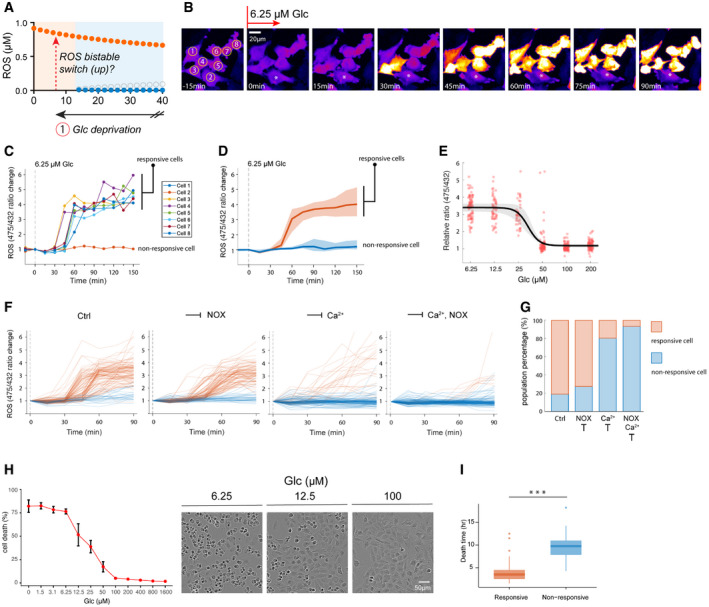
Redox catastrophe as a ROS bistable switch at the lethal glucose threshold A–DROS bistable switch upon glucose deprivation at 6.3 µM. (A) Illustration of an experimental strategy to induce the ROS bistable switch by lowering glucose to 6.3 µM. Time‐lapse images (B) and quantification (C) of ROS in individual cells. The asterisk‐labeled cell in (B) represents a non‐responsive cell with no increase in ROS after glucose starvation. (D) The median (solid line), as well as Q1 (lower bound) and Q3 (upper bound), of ROS levels for responsive (red) and non‐responsive (blue) cells after glucose starvation at 6.3 µM (*N* > 100).EA dose–response curve of ROS 1.5 h after glucose deprivation (from 200 to 6.3 µM). For each glucose concentration, the ROS levels of 80 cells were quantified. To deconvolute the effects of non‐responsive cells, they were not considered in the analyses of the lower three glucose concentrations (6.3 µM, 12.5 µM, and 25 µM). A Hill exponent (*n*) was fitted to be 6.2, with a 95% confidence interval of 4–8.FTemporal changes of single‐cell ROS after glucose deprivation at 0 µM (left panel, ctrl) or with additional inhibition of NOX (GKT137831, 10 µM, middle‐left panel) or calcium (BAPTA‐AM, 12.5 µM, middle‐right panel) signaling or inhibition of both (right panel).GPercentages of non‐responsive and responsive cells after glucose deprivation at 0 µM with or without inhibitors of calcium (BAPTA‐AM, 12.5 µM) and NOX (GKT137831, 10 µM) signaling. Three experimental repeats generated qualitatively identical results.HLeft panel: cell death was quantified with YoYo‐1 dye 6 h after glucose treatments ranging from 1.6 mM to 0 µM. Error bars represent standard deviations of six technical repeats. Three experimental repeats generated quantitatively similar results. Right panel: representative bright‐field cell images 6 h after glucose deprivation.IDeath times for responsive and non‐responsive cells are significantly different (*N* > 100, Mann–Whitney *U*‐test ***P‐value = 3.28 × 10^−11^). On each box, the central band indicates the median, and the bottom and top edges of the box indicate the 25^th^ (q1) and 75^th^ (q3) percentiles, respectively. The whiskers (upper one = q3 + 1.5(q3 − q1), lower one = q1–1.5(q3 − q1)) extend to the most extreme data points not considered outliers, and the outliers are plotted individually using the dot symbol.

We sought to experimentally explore the existence of redox bistability during glucose deprivation in human cells. To assess ROS dynamics, we utilized a genetically encoded hydrogen peroxide (H_2_O_2_) probe, HyPer7 (Pak *et al*, 2020), for single‐cell H_2_O_2_ quantification. Unlike conventional chemical‐based ROS reporters (e.g., DCF‐DA), HyPer7 is reversible and responds quantitatively to H_2_O_2_ in the low nanomolar range, thereby permitting real‐time measurements of H_2_O_2_ dynamics and its steady state in the relevant concentration range. We starved HyPer7‐expressing LN18 cells, a glucose‐addicted human glioblastoma cell line, with a glucose concentration (6.3 µM) below the predicted glucose threshold to examine the occurrence of a ROS bistable switch (Fig 4A). As illustrated in Fig 4B and C, we observed sharp increases in ROS level (≥ 2‐fold elevation from basal levels) in individual cells within a timeframe of 0.5–1.5 h. Approximately 80% of cells displayed this rapid increase in ROS in response to low‐glucose levels, accordingly termed responsive cells (see Fig 4C for single‐cell measurements and Fig 4D for a population‐level assessment of 100 cells). Non‐responsive cells exhibited a less than twofold increase in ROS within 1.5 h (as an example, see the asterisk‐labeled Cell 2 in Fig 4B). It is worth noting that due to heterogeneity of ROS dynamics within a cell population, such as arising from responsive versus non‐responsive cells (Fig 4B) and ROS increase time (Fig 4C), single‐cell quantification is necessary to reveal these sharp increases in cellular ROS levels, which can be easily masked by population‐level measurements.

If the redox system in LN18 cells is indeed bistable, they would display a threshold‐like, ultrasensitive ROS response to different levels of glucose starvation. Indeed, the majority of LN18 cells only exhibit ROS increases when the glucose concentration is ≤ 25 µM, which is ≈ 2 orders of magnitude lower than the physiological glucose concentration (Fig 4E). For the dose‐response curve, a Hill exponent (*n*) was fitted to be 6.2, with a 95% confidence interval of 4–8 (Fig 4E) suggesting ROS is ultrasensitive to glucose concentration. Similar to LN18, another glucose‐addicted cell line (U87‐MG) also presented the same pattern of elevated ROS when the glucose concentration was ≤ 25 µM (Appendix Fig S1A) and exhibited an ultrasensitive dose‐response curve with a Hill exponent (*n*) of 6.5 and a 95% confidence interval of 2–11 (Appendix Fig S1B).

Previous studies have demonstrated the contribution of NOX and calcium signaling to elevated ROS during glucose starvation (Graham *et al*, 2012). ROS elevation mediated by these signaling pathways activates tyrosine kinases, which then feed back to further amplify ROS. To test the involvement of these two major ROS feedback signaling loops in the switch‐like ROS response, we deployed chemical inhibitors to inhibit NOX (GKT137831: NOX1/4 inhibitor, 10 µM) and calcium (BAPTA‐AM, 12.5 µM, Appendix Fig S2A) signaling during glucose starvation (0 µM). Consistent with previous findings, glucose starvation induced tyrosine kinase phosphorylation, as measured by immunoblotting (Appendix Fig S2B). Suppression of either NOX or calcium diminished tyrosine phosphorylation (21.5 and 85.3% inhibition, respectively), and dual inhibition completely abolished glucose starvation‐induced tyrosine kinase phosphorylation (Appendix Fig S2B).

Accordingly, quantification of ROS levels in individual cells shows that suppression of either NOX or calcium signaling compromised the bistable ROS switch (Fig 4F) and increased the percentage of non‐responsive cells (NOX inhibition: 19.1–27.5%; calcium inhibition: 19.1–80.7%, Fig 4G). Further, the percentage of non‐responsive cells is higher (93.6%) when both signaling pathways are suppressed (Fig 4G), indicating their dual involvement in the switch‐like elevation of ROS. Notably, the degree of ROS suppression after single or dual inhibitor treatments (Fig 4F and G) is quantitatively consistent with their respective inhibitory strength in tyrosine kinase phosphorylation (Appendix Fig S2B), supporting the model of NOX‐ and calcium‐mediated ROS feedback loops upon glucose deprivation.

Phenotypically, the sharp increase in ROS level we observed in LN18 cells was usually followed by cell death (Fig 4H), as indicated by plasma membrane blebs (right panel images, Fig 4H), supporting the notion of an underlying ROS bistable switch during redox catastrophe. Similar to the ROS response curve in Fig 4E, cell death also followed a dose‐response curve with a cell death threshold around 12.5–50 µM (Fig 4H), which is consistent with our model analysis (Fig 3B), as well as previous findings that human cells can survive at a low micromolar range of glucose (Lee *et al*, 1998, 2018). Interestingly, the non‐responsive cells survived significantly longer than responsive cells after glucose deprivation (Fig 4I), further demonstrating the link between the occurrence of ROS bistable switch and cell death.

### Irreversibility and hysteresis of the redox system in glucose‐addicted LN18 cells

**Figure 5:**
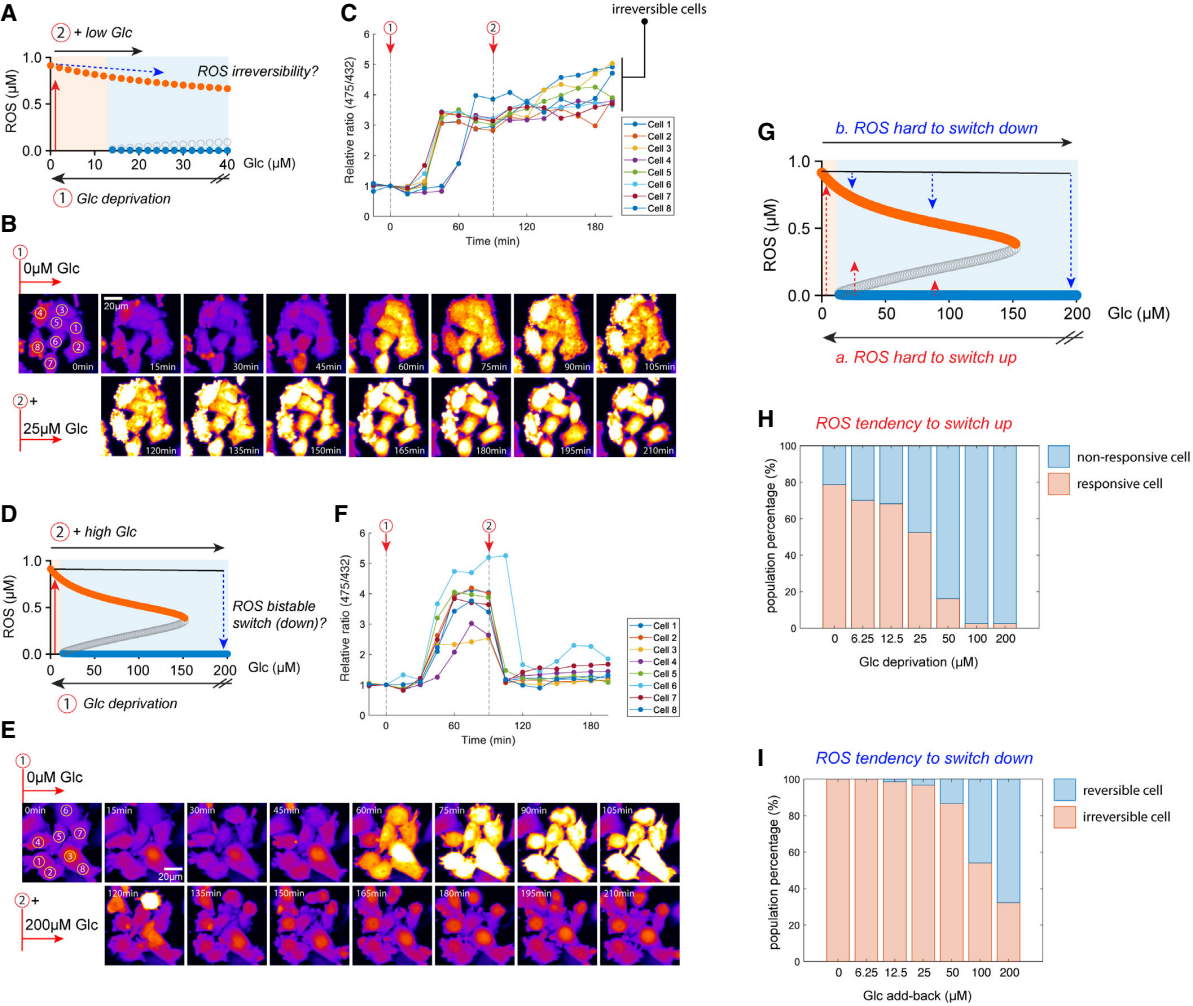
Irreversibility and hysteresis in the response of ROS to glucose perturbations A–CIrreversibility of ROS after adding back a low level of glucose (25 µM). (A) Illustration of the experimental test for ROS irreversibility: (1) lowering glucose (Glc) to 0 µM to induce an upswitch in ROS level; and (2) adding back 25 µM glucose and measuring the change in ROS. Time‐lapse images (B) and quantification (C) of ROS fold‐change in individual cells.D–FROS levels are switched down upon adding back a high level of glucose (200 µM). (D) Illustration of a ROS switch‐down experiment: (1) lowering glucose to 0 µM to induce an upswitch in ROS level; and (2) adding back 200 µM glucose and measuring the change in ROS. Time‐lapse images (E) and quantification (F) of ROS fold‐change in individual cells.GIllustration of ROS hysteresis (history dependence) in response to glucose perturbations. a: it is difficult to switch up ROS when glucose levels are lowered. b: It is difficult to switch down ROS when glucose levels increase.H, I(H) Percentages of responsive/non‐responsive cells 75 min after glucose starvation at different glucose concentrations. (I) Percentages of irreversible/reversible cells 75 min after adding back various glucose concentrations. Only the responsive cells are considered in this analysis. Three experimental repeats generated comparable differences in ROS tendency to switch up (H) or down (I).

One key characteristic of a bistable system is its irreversibility, i.e., once the system switches to a different steady state, it is robust against perturbations (e.g., ROS remains high despite a small add‐back glucose perturbation, Fig 5A), unless the perturbation is so large that it exceeds a threshold that reverts the system to the original state (e.g., ROS declines after a large glucose add‐back perturbation, Fig 5D). We tested if the redox system in LN18 cells exhibits irreversibility. As shown in Fig 5B and C, once the ROS level switched to an upper steady state during glucose starvation (0 µM), it remained high even after adding back a low level of glucose (25 µM). In contrast, when the concentration of glucose added back was high (200 µM), ROS levels rapidly and consistently declined to the lower steady state (Fig 5E and F).

In a broader sense, this characteristic irreversibility demonstrates the hysteretic (history‐dependent) behavior of bistable systems. In the context of the redox system responding to glucose perturbations, this means that cells are not easily committed to switching to higher ROS when glucose levels are lowered (Fig 5G; a: hard to switch up, red arrows), but once they surpass the threshold and have switched up, they tend to retain a higher ROS level even if the glucose concentration then increases (Fig 5G; b: hard to switch down, blue arrows). Accordingly, we measured the cellular tendency to elevate ROS levels at various low‐glucose concentrations (0–200 µM), represented by the percentage of responsive cells in the population (Fig 5H). Similarly, we measured the tendency for cells to switch ROS down represented by the percentage of irreversible cells (Fig 5C) in responsive cells after adding back various glucose concentrations (Fig 5I). Comparing Fig 5H and I, hysteresis of the redox system is manifested as the greater tendency for cells to remain at either low or high ROS, before and after ROS switch‐up, as indicated by the difference between the percentages of responsive cells (Fig 5H) and those of irreversible cells (Fig 5I) during glucose deprivation and glucose add‐back, respectively.

### Cystine and glutamine alter redox dynamics and the bistable switch after glucose deprivation

**Figure 6:**
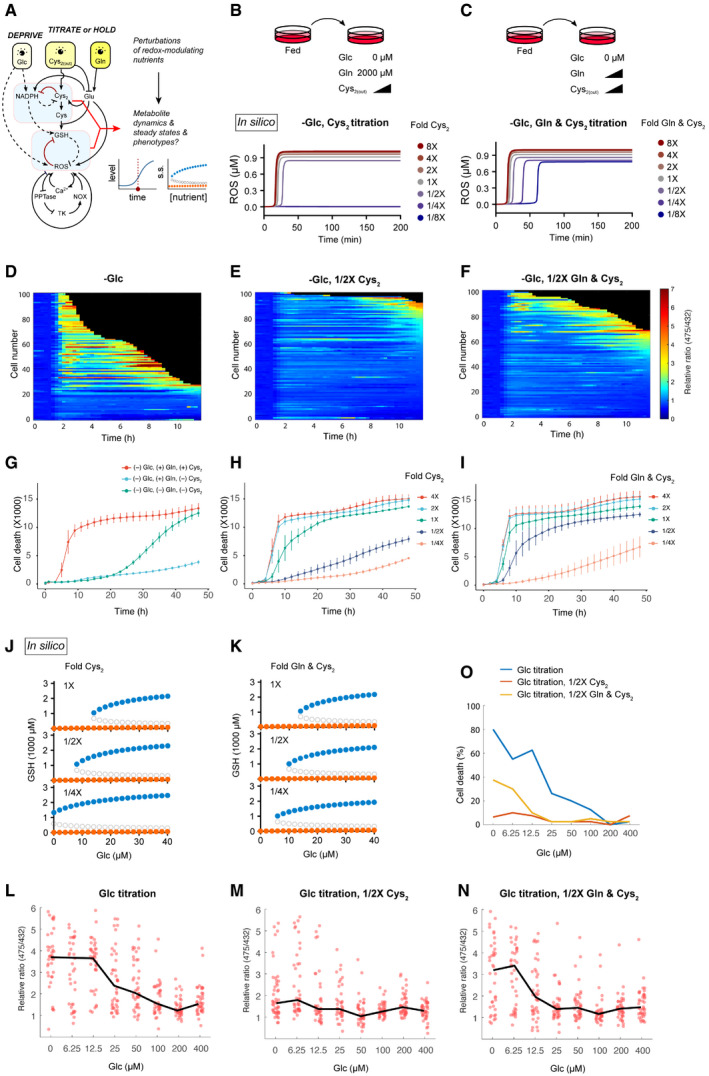
Nutrient interaction modulates the glucose threshold of the redox bistable switch AIllustration of our *in silico* simulation strategy for revealing the effects of perturbing cystine and glutamine on redox dynamics and steady state upon glucose deprivation.B, CDesign of perturbations of cystine alone (B) or cystine and glutamine (C) during glucose deprivation. Lower panels are simulations of temporal dynamics of ROS.D–FHeatmaps of ROS levels in 100 single cells after glucose deprivation (D), or in combination with 1/2X titration of cysteine (E), or cysteine and glutamine (F).G–ICell death was quantified with YOYO‐1 dye upon glucose deprivation (G), or in combination with titrations (H & I) of cystine, or cystine and glutamine. Error bars represent standard deviations of six technical repeats. Three experimental repeats generated quantitatively similar results.J, KBifurcation diagrams of GSH upon glucose deprivation and with additional perturbations of cystine (J) or cystine and glutamine (K).L–NROS response curves 10 h after glucose deprivation (from 400 µM to 0 µM, (L)) or in combination with 1/2X titrations (M, N) of cystine, or cystine and glutamine. For each glucose concentration, the ROS levels of 80 cells were quantified either 10 h after glucose deprivation or before cell death. The black lines connect the medians of ROS levels from each [glucose].OPercentages of death cells 10 h after glucose deprivation or in combination with 1/2X titrations of cystine, or cystine and glutamine.

**Figure EV3:**
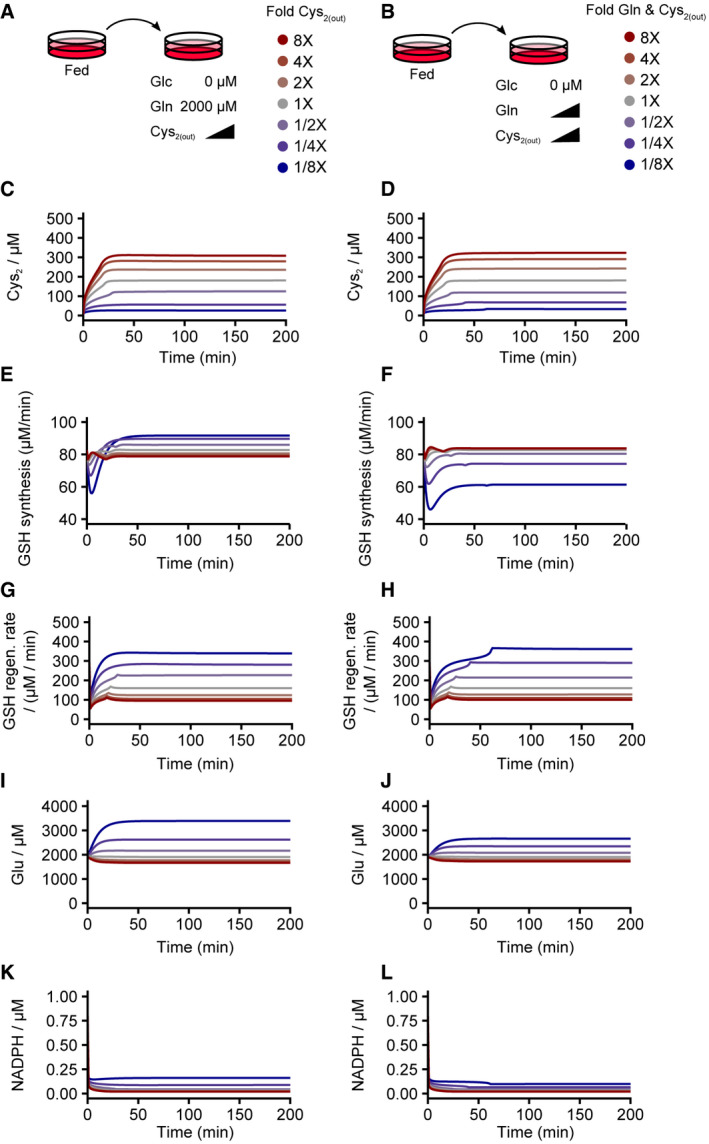
Metabolite and redox dynamics after glucose deprivation coupled with titration of cystine or co‐titration of cystine and glutamine A–LMetabolite and redox dynamics after glucose deprivation coupled with titration of cystine (A, C, E, G, I, K; left column) or co‐titration of cystine and glutamine (B, D, F, H, J, L; right column). Steady‐state cystine (C and D) and NADPH (K and L) concentrations and GSH regeneration rates (G and H) were comparable between cystine single‐titration and cystine plus glutamine co‐titration, whereas steady‐state glucose concentrations (I and J) and the *de novo* GSH synthesis rate (E and F) differed.

Since cystine and glutamine metabolism alters the cellular redox state via consumption and production of NADPH and glutathione, we explored if glucose‐dependent redox dynamics and the bistable switch are altered by these two redox‐modulating nutrients (Fig 6A). We simulated glucose deprivation in conjunction with a titration of extracellular cystine alone (Fig 6B) or co‐titration of cystine and glutamine (Fig 6C). Increasing the extracellular cystine concentration (from onefold to eightfold: 200–1,600 µM) accelerated the accumulation of intracellular cystine (Fig EV3C) and occurrence of redox catastrophe (bottom panel, Fig 6B), consistent with previous observations of cystine toxicity under conditions of glucose deprivation (Goji *et al*, 2017; Joly *et al*, 2020; Liu *et al*, 2020). In contrast, dilution of extracellular cystine (from 1‐ to 1/8‐fold: 200 to 25 µM) delayed intracellular cystine accumulation and prevented redox catastrophe (Fig EV3C & bottom panel, Fig 6B). In fact, when extracellular cystine was sufficiently diluted (i.e., 1/4‐ or 1/8‐fold dilutions), redox catastrophe could even be rescued (bottom panel, Fig 6B), in agreement with recent experimental observations (Goji *et al*, 2017; Joly *et al*, 2020; Liu *et al*, 2020). Similarly, co‐dilution of glutamine and cystine delayed cystine accumulation and redox catastrophe. However, this led to the compromise in the complete rescue from redox catastrophe at low cystine dilutions (bottom panel, Fig 6C).

To experimentally assess these simulation results, we quantified HyPer7 signals upon glucose starvation (Fig 6D), as well as in combination with titrations of cystine (Fig 6E) or both glutamine and cystine (Fig 6F). In stark contrast to the rapid ROS increase during glucose deprivation (Fig 6D), ROS levels were greatly suppressed when only half the amount of cystine was titrated (Fig 6E). This ROS suppression due to lower cystine was compromised when we co‐titrated glutamine (Fig 6F compared with Fig 6E). Phenotypically, cystine deprivation rescued glucose deprivation‐induced cell death (Fig 6G), and this rescue effect was compromised when glutamine was also deprived (Fig 6G). Consistently, our titration experiments reveal that glucose deprivation‐induced cell death is quantitatively modulated by the concentrations of cystine (Fig 6H) and glutamine (Fig 6I).

Next, we sought to infer the mechanism underlying these observations from our model. In our simulations, lowering cystine led to increases in both *de novo* GSH synthesis and regeneration (Fig EV3E and G). Despite cystine is a precursor metabolite for *de novo* GSH synthesis, cystine dilution is compensated for by the reduced export of glutamate, another precursor metabolite for GSH synthesis (Fig EV3I), which even enables a slight increase in GSH synthesis. Moreover, more NADPH becomes available for GSH regeneration when cystine is depleted (Fig EV3K). Consequently, the overall GSH production (regeneration plus *de novo* synthesis) is sufficient to suppress ROS accumulation. In the case of glutamine and cystine co‐dilution where redox catastrophe cannot be prevented (bottom panel, Fig 6C), it is most likely due to the diminished glutamate production by lowering glutamine levels (Fig EV3J), leading to compromised GSH synthesis (Fig EV3F).

Since extracellular cystine and glutamine modulate the kinetics of redox catastrophe in both simulations and experiments (Fig 6B–I), we then examined how the redox system's bistability can be altered upon dilutions of cystine and glutamine during glucose deprivation. Our simulations showed that both cystine dilution alone (Fig 6J) and co‐dilution of cystine and glutamine (albeit to a lesser extent, Fig 6K) stretched the GSH bifurcation point leftward. At a sufficient dilution of cystine alone (1/4‐fold: from 200 to 50 µM), the bifurcation point was extended to the physically impossible “negative” glucose regime, rendering the high‐GSH/low‐ROS state stable even at 0 µM glucose (upper panel, Fig 6J). In contrast, co‐dilution of cystine and glutamine could not stabilize the high‐GSH/low‐ROS state near 0 µM glucose as the bifurcation point could not extend lower than ≈ 5 µM glucose (upper panel, Fig 6K). To test the leftward shift of the GSH bifurcation point, we quantified both ROS (via HyPer7) and cellular phenotype (cell death) 10 h (to minimize non‐responsive cell effects) after a series of glucose concentrations in combination with half‐fold titrations of cysteine or cysteine and glutamine (Fig 6L–O). Consistent with our simulation results, cystine titration suppressed the ROS elevation in the majority of the cell population upon titrating glucose down to 0 µM, supporting the leftward shift of the high‐GSH/low‐ROS steady state (compare Fig 6M with Fig 6L) and rescuing cells from death (Fig 6O). However, co‐titration of glutamine with cysteine compromised this leftward shift in the ROS response curve, with the majority of the cell population exhibiting an increase in ROS at 6.25 µM glucose (compare Fig 6N with Fig 6M) and consequently rescue of the cellular phenotype (Fig 6O). Together, these results elucidate how the three redox‐regulating nutrients—glucose, cystine, and glutamine—govern the dynamics and bistability of the human redox system.

## Discussion

Glucose is a core metabolite catabolized, paradoxically, for the both production (TCA cycle and OXPHOS) and the consumption (oxPPP) of ROS. Combining modeling and experimental approaches, our study reveals the existence of a tight, lethal glucose threshold in glucose‐addicted human cells. Above this threshold, a stable, physiological GSH concentration (> 2 mM) is maintained down to a low micromolar glucose range (≈ 100 μM). When extracellular glucose falls below that threshold, the cellular antioxidant defense system fails, ROS rapidly accumulate, and oxidative cell death ensues (Figs 2, 3, 4). This phenomenon of redox catastrophe characterized by swift changes in ROS and GSH represents a bistable transition between two distinct redox states (Figs 2, 3, 4, 5). Many all‐or‐none and irreversible biological phenomena are regulated by bistability, e.g., cell cycle progression and stem cell differentiation. How can a bistable redox system benefit human cells or biological systems in general?

As a major determinant of the cellular redox state, ROS plays a complex role in cell fate decisions. At physiological levels, ROS promotes cellular signaling for survival and proliferation. In contrast, supra‐physiological levels of ROS can cause oxidative damages, leading to cell death. Hence, a bistable ROS system that imparts a characteristic switch‐like and irreversible response could elicit distinct all‐or‐none cell fate decisions. In addition, a bistable system of feedback controls enables the prevention of oxidative stress and maintenance of redox homeostasis. For instance, in the presence of fluctuating nutrient levels, a bistable redox system allows cellular responses to operate in a threshold‐like manner that helps reduce ROS stress. Without redox bistability, ROS could accumulate in a graded manner and become a significant source of stress given that mammalian cells can experience oxidative stress at an intracellular H_2_O_2_ level as low as 100 nM (Huang *et al*, 2016; Sies & Jones, 2020). Bistability allows for a mild ROS increase in response to a large decrease in nutrients (Fig 5G; a: hard to switch up, red arrows), even at the lower micromolar range, which is about two orders of magnitude lower than physiological glucose concentration. Moreover, bistability enables cellular resistance to sudden devastating nutrient shortages in the environment. Compared to common cell culture media, such as RPMI 1640 (11.11 mM glucose) and DMEM (25 mM glucose), human plasma typically has a lower glucose level (5 mM). Glucose concentration can even be lower within tumor microenvironments, which may prevent T‐cell activation (Ho *et al*, 2015). Hence, the ability of tumor cells to tolerate low‐glucose environments is directly linked to their survival. In the case of *in vitro* cell cultures, where glucose is not repleted by active circulation, extracellular glucose can also be low due to consumption. Thus, the ability of bistability to dampen ROS increases in scenarios of fluctuating extracellular glucose can be critical for redox homeostasis and cell survival in various cellular contexts.

Considering the essentiality of redox reactions for all living systems, redox bistability is likely important for all organisms. Most organisms constantly face the challenge of nutrient fluctuations, and how they maintain redox homeostasis under starvation conditions is important for their survival. The mutually inhibitory relationship between ROS and antioxidants, embedded in their biochemistry, is the core architecture for redox bistability. The metabolic origin of ROS from the ETC, production of GSH and NADPH from nutrients, as well as antioxidant defenses such as peroxiredoxins and GSH peroxidases, are all well conserved from bacteria to eukaryotes (Knoops *et al*, 2007; Margis *et al*, 2008). Therefore, it is tempting to speculate that the bistable redox system has a deep root in evolution.

One key mechanism underlying the bistable redox transition is depletion of NADPH below a critical concentration at which GSH consumption overwhelms production (Fig EV2). Consistent with a previous study (Goji *et al*, 2017), this limitation in NADPH availability can be alleviated if extracellular levels of the NADPH‐consuming nutrient cystine are diminished (Figs 6 and EV3). In addition, a low level of cystine better enables retention of intracellular glutamate for *de novo* GSH synthesis, stabilizes the low‐ROS/high‐GSH state, and prevents cell death even at 0 µM glucose (Fig EV3). Interestingly, when glutamine is co‐titrated with cystine, this rescue effect is compromised. Mechanistically, glutamine participates in both the GSH regeneration rate (via NADPH synthesis) and *de novo* GSH synthesis (via glutamate generation; Fig EV3). Our simulation results indicate that co‐titration of glutamine with cystine abrogates *de novo* GSH synthesis due to a shortage of intracellular glutamate (Fig EV3). Taken together, the occurrence of redox catastrophe in human cells depends on the availability of multiple redox‐modulating nutrients, i.e., glucose, cystine, and glutamine (Fig 6). This notion of an adjustable, nutrient‐based threshold for redox catastrophe is especially critical when considering targeted therapies for cancer metabolism. Cancer cells have been well characterized as displaying a heavy reliance on these redox‐modulating nutrients. However, various cancer types exhibit diverse metabolic profiles, so they can exhibit differential sensitivity to redox perturbations. Our study provides a quantitative framework for predicting the redox sensitivity of cancer cells based on their metabolic signatures, for instance nutrient uptake, in order to tailor strategies to induce redox catastrophe in those cells.

**Figure EV4:**
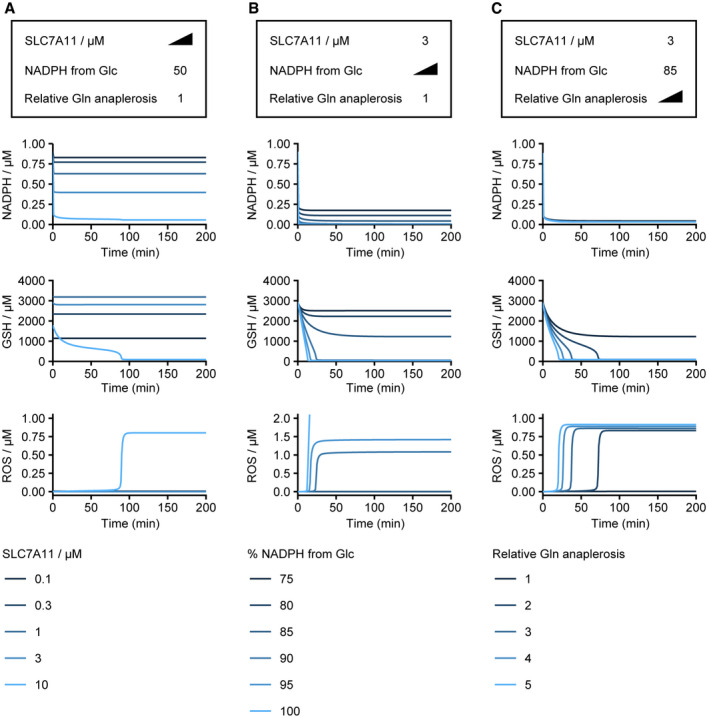
Nutrient‐redox model recapitulates *in silico* various susceptibility factors to glucose deprivation A–CEffects of *SLC7A11* expression level (A), dependence on glucose to regenerate NADPH (B), and upregulation of glutamine anaplerosis into TCA cycle/OXPHOS under conditions of high SLC7A11 expression (C) on redox dynamics upon glucose deprivation (from 10,000 to 0 μM). (A) Simulation with neutral dependence on glucose for NADPH regeneration and no upregulation of glutamine anaplerosis. Note that *SLC7A11* overexpression decreases intracellular glutamine through export, thereby limiting the contribution of glutamine to ROS production via OXPHOS in our model. (B) Simulation with varying glucose dependencies for NADPH under conditions of *SLC7A11* overexpression and no upregulation of glutamine anaplerosis. (C) Simulation with varying degrees of upregulated glutamine anaplerosis and *SLC7A11* overexpression and a dependency on glucose for NADPH regeneration. See Materials and Methods for *in silico* perturbation of glucose dependence of NADPH reduction (B) and strength of glutamine anaplerosis (C).

In this study, our aim was to build a quantitative and realistic nutrient‐redox model. To do so, we estimated model parameters from quantified metabolic fluxes, metabolite concentrations, or fold changes between various conditions arising from a catalog of studies using different human cell lines (see Materials and Methods). Consequently, the estimated parameter set represents an “averaged” metabolic configuration of human cells. Interestingly, though this “averaged” parameter set is resistant to glucose deprivation, parameter sets mimicking the hallmarks of glucose addiction sensitized the model to glucose deprivation (Fig EV4) by modulating the bistability of the redox system. This *in silico* recapitulation of glucose addiction affirms key drivers for glucose addiction, including (i) *SLC7A11* overexpression (Fig EV4A) (Goji *et al*, 2017; Koppula *et al*, 2017; Shin *et al*, 2017; Joly *et al*, 2020; Liu *et al*, 2020); (ii) reliance on glucose/oxPPP for NADPH regeneration (Fig EV4B) (Liu *et al*, 2020); and (iii) SLC7A11‐driven glutamate anaplerosis (Fig EV4C) (Muir *et al*, 2017). Hence, our model defines a set of metabolic parameters that quantitatively reflect the metabolic underpinnings of glucose addiction in human cells. More broadly, measuring and mapping various human cell lines/cell types within this parameter space can help establish cell‐specific nutrient dependencies and assist in rationalizing potential strategies for targeting redox imbalance in cancer cells.

Our nutrient‐redox model mimics well the phenomenon of redox catastrophe in a glucose‐addicted cell line (Figs 2, 3, 4 and (Joly *et al*, 2020). Our model was built under several assumptions: (i) no intracellular glucose reservoir; (ii) no regulatory relationship among nutrient concentrations, nutrient uptake rates, or metabolic rate constants; (iii) absence of de novo cysteine synthesis via the trans‐sulfuration pathway in high xCT‐expressing cells (Zhu *et al*, 2019); and (iv) no heterogeneity in nutrient metabolism or ROS signaling among individual cells. By relaxing those assumptions, we expect our model could be utilized to present stronger predictive power and could further be generalized to other regulatory contexts of cellular metabolism. For example, our experimental results for ROS responsiveness (Fig 4B–E) reveal cell–cell heterogeneity, i.e., ROS bistable switches in only ≈ 80% of cells. It is possible that individual cells display slightly divergent bifurcation points due to differing states of the nutrient‐redox network, such as nutrient uptakes and/or tyrosine kinase signaling activities. Moreover, cells having a larger intracellular glucose reservoir tend to be more robust to glucose deprivation or display delayed glucose deprivation‐induced cell death (Lee *et al*, 2018). This intracellular glucose reservoir, likely glycogen (Yang *et al*, 2015), may buffer the rate of NADPH depletion and delay reductions in GSH levels and thus redox catastrophe and cell death. Such buffering may allow time for signaling and gene expression to alter the cellular redox system. For example, 5′ AMP‐activated protein kinase (AMPK) can be activated upon glucose deprivation and restricts NADPH consumption in anabolic reactions, thereby preventing redox catastrophe and achieving metabolic adaptation (Jeon *et al*, 2012). Finally, to better understand cell type‐ and context‐dependent metabolic regulation, such as the paradoxical role of *SLC7A11* in regulating the glucose deprivation response (Koppula *et al*, 2017; Shin *et al*, 2017; Liu *et al*, 2020), requires integration of large‐scale quantitative data across different cell types in a generalizable, core mathematical model. Accordingly, further investigations into the signaling metabolism/drug–metabolism interfaces are necessary and extensions of our nutrient‐redox model are warranted to accommodate the wealth of redox regulation and adaptive strategies across various types of human cells.

## Materials and Methods

### Reagents

Glucose stock (20 mM) was prepared, confirmed using a GlucCell™ glucose meter, and stored at −80°C before usage. Dialyzed FBS (dFBS) was purchased from Gibco. To ensure its glucose concentration was lower than 1 µM, small molecules in the dFBS were filter‐diluted at least 125‐fold (5‐fold × 3) using an Amicon Ultra‐15 centrifugal filter. GKT137831 (Cat. No. HY‐12298) was purchased from MedChemExpress, and nifedipine (Cat. No. 11106) and dasatinib (Cat. No. 11498) were purchased from Cayman Chemical. BAPTA‐AM (Cat. No. ab120503) was purchased from Abcam. Fluo‐4 AM, a cell permeable Ca^2+^ indicator (Cat. No. F14217), was purchased from Invitrogen and stored in −20°C before usage. The chemicals were generally prepared into high concentration stocks in DMSO and stored in −80°C before usage. Phospho‐Tyrosine (4G10) Mouse mAb (Cat. No. 96215) was purchased from Cell Signaling Technology.

### Cell culture

Human brain glioblastoma LN18 cells were purchased from ATCC (CRL‐2610). U87‐MG parental cells (BCRB No. 60360), human brain glioblastoma cell line, were purchased from Bioresource Collection and Research Center (BCRC) in Taiwan. The ultrasensitive hydrogen peroxide indicator HyPer7 was purchased from Addgene (#136466) and cloned into a lentiviral backbone using the Gateway recombination system. HyPer7‐containing lentiviruses were produced by HEK293T transfection. LN18 and U87‐MG cells were infected with the HyPer7‐containing lentiviruses, followed by sorting for the top 5% of HyPer7‐expressing cells by means of flow cytometry. Polymerase chain reaction confirmed the LN18 and U87‐MG parentals as well as their ROS reporters (LN18‐HyPer7, and U87‐MG‐HyPer7) cells to be mycoplasma‐free. All cells were cultured in DMEM medium supplemented with 5% fetal bovine serum (FBS). Cells were kept at maximum 50% confluency and incubated at 37°C with 5% CO_2_. For time‐lapse imaging of ROS in individual cells, cells were grown in DMEM without phenol red and supplemented with 5% FBS in a iBidi 96‐well µ‐Plate for 2 days before imaging. To monitor the intracellular Ca^2+^, cells were stained with Fluo‐4 AM (1 µM) for 1 h followed by 30‐min incubation without Fluo‐4 AM for de‐esterification. Pre‐treatment of 10 µM nifedipine and 12.5 µM BAPTA‐AM was performed 1 h before glucose deprivation. To quantify cell death kinetics, parental LN18 cells were grown in DMEM without phenol red but containing YOYO‐1 iodide as a cell death indicator in Nunc 96‐well plates (Thermo Fisher Scientific) for 2 days before imaging.

### Live‐cell imaging

To monitor single‐cell ROS dynamics, time‐lapse imaging experiments were performed using a Nikon Eclipse TE‐2000 inverted microscope equipped with a Nikon Perfect Focus System and a Hamamatsu Orca ER camera under conditions of controlled temperature (37°C), atmosphere (5% CO_2_), and humidity (90–100%). Cells were excited at both 432 and 475 nm (5% intensity and 150 ms exposure) with 432/36–25 nm and 475/25–25 band‐pass filters, a 550‐nm dichroic mirror, and a 536/40–25 nm emission filter. Images were acquired every 15 min with a 20× Nikon plan apo objective (NA 0.75). Ratiometric readouts of F475 nm/F432 nm were acquired to report ROS levels. To quantify cell death kinetics, time‐lapse imaging experiments were performed using the IncuCyte S3 Live‐Cell Analysis System (Essen BioScience) with a 10× objective at 37°C and 5% CO_2_. Bright‐field and green fluorescent channels were used to capture cellular morphology and YOYO‐1 iodide fluorescence signal, respectively. To monitor the intracellular Ca^2+^, Fluo‐4 AM, cells were excited at 475 nm (5% intensity and 200 ms exposure) with 475/35–25 band‐pass filter, a 500‐nm dichroic mirror, and a 536/40–25 nm emission filter. Image analyses and quantifications of cell death based on YOYO‐1 iodide fluorescence signal were performed using the analysis tool in the IncuCyte S3 Live‐Cell Analysis System.

### Image processing

Image processing was conducted in ImageJ and using custom MATLAB scripts. Background subtraction (rolling ball radius: 50 pixels) and generation of ratiometric images were achieved using ImageJ with a custom script to process the image sets automatically. Cells were then tracked using a semi‐automated MATLAB program described previously (Reyes *et al*, 2018). Cell death events were manually annotated based on the morphology (plasma membrane blebs) of dead cells.

### Western blot analysis

LN18 parental cells were grown in DMEM without phenol red and supplemented with 5% FBS in a Nunc six‐well plates (Thermo Fisher Scientific) for 2 days. Cells were then treated with chemical inhibitors 1 h before glucose deprivation. Proteins were then harvested 2 h after glucose starvation. Phospho‐tyrosine levels were probed with phospho‐tyrosine (4G10) Mouse mAb (Cat. No. 96215) and quantified using ImageJ.

### Mathematical modeling

#### General considerations for cell biology

We modeled cellular redox homeostasis as a steady‐state balance of metabolites and signaling molecules. In Fig 1A, we provide a graphical outline of the metabolic processes considered in this study. We focused on metabolism of glucose, glutamine, and cystine. Glucose and glutamine fuel mitochondrial metabolism and ROS production, and all three nutrients directly affect antioxidant metabolism either by regenerating/consuming NADPH or by acting as substrates for GSH synthesis. By parameterizing extracellular nutrient concentrations, we were able to model the effect of starvation on redox metabolism and homeostasis. We specifically considered the consumption of NADPH upon cystine reduction (Pader *et al*, 2014), as it has been shown to be a strong driver of glucose deprivation‐induced cell death (Goji *et al*, 2017; Joly *et al*, 2020; Liu *et al*, 2020). However, oxidative stress is known to increase rapidly following glucose deprivation (Graham *et al*, 2012; Goji *et al*, 2017) by self‐amplifying positive feedback loops, including those of NOX signaling (Graham *et al*, 2012) or Ca^2+^ signaling (Hempel & Trebak, 2017; Lee *et al*, 2018; Maher *et al*, 2018). We also considered an upper bound for ROS production in the form of a negative feedback loop. Several mechanisms may underlie this autoinhibition of ROS, such as saturation of ROS‐producing enzymes/protein complexes, reversible oxidative inhibition of multiple TCA cycle/OXPHOS genes (Nulton‐Persson & Szweda, 2001), and possibly irreversible destruction of enzymes or mitochondria (Scherz‐Shouval & Elazar, 2011). In this study, we assumed that nutrients do not regulate the activity of metabolic enzymes, either directly or indirectly, via signaling processes on the timescale of the glucose deprivation response. The effects of such regulatory processes can be studied further using our model. Parameters used in this study are summarized in Appendix Table S9.

#### Modeling the nutrient‐redox relationship with a system of ordinary differential equations

##### General considerations for reaction terms

Generally, we assume reactions follow mass action without *a priori* knowledge, with the exceptions of nutrient uptake and ROS autoinhibition. For nutrient uptake, the glucose, glutamine, and cystine‐glutamate transporters are known to be saturated by nutrient concentrations in the culture media (Appendix Table S3) and are therefore modeled by hyperbolic functions. Note that glutamine uptake and glutamate production are considered together, and glucose uptake is likewise considered with the NADPH and ROS production terms.

##### Cystine

We considered cystine uptake by SLC7A11. Saturation of the antiporter by extracellular cystine (*Cys_2_*) was modeled as a hyperbolic function. The cystine uptake rate depends on the levels of *SLC7A11* expression and intracellular glutamate (*Glu*). Of note, we assumed steady‐state *SLC7A11* expression, overlooking its regulation given the timescale of the glucose deprivation response. However, we assumed that cystine is quickly reduced upon uptake and represents the main reaction responsible for cystine removal.

$$\begin{array}{ll} \frac{d{\left[Cys_{2}\right]}_{\left(t\right)}}{\mathrm{dt}}= & \;k_{Cys_{2}\_\text{Import}}\frac{{\left[Cys_{2}\right]}_{\text{External}}}{K_{Cys_{2}\_\text{Import}}+{\left[Cys_{2}\right]}_{\text{External}}}\left[SLC7A11\right]{\left[\mathrm{Glu}\right]}_{\left(t\right)}\\ & \;‐k_{Cys_{2}\_\text{Reduction}}{\left[Cys_{2}\right]}_{\left(t\right)}{\left[\text{NADPH}\right]}_{\left(t\right)}. \end{array}$$

##### Cysteine

We modeled cysteine (*Cys*) production from cystine reduction, cysteine consumption by GSH synthesis, and general cysteine consumption/degradation by other processes.

$$\begin{array}{ll} \frac{d{\left[\mathrm{Cys}\right]}_{\left(t\right)}}{\mathrm{dt}}= & \;2k_{Cys_{2}\_\text{Reduction}}{\left[Cys_{2}\right]}_{\left(t\right)}{\left[\text{NADPH}\right]}_{\left(t\right)}\\ & \;‐\;k_{Cys\_\text{Degradation}}{\left[\mathrm{Cys}\right]}_{\left(t\right)}‐k_{\text{GSH}\_\text{Production}}{\left[\mathrm{Cys}\right]}_{\left(t\right)}{\left[\mathrm{Glu}\right]}_{\left(t\right)}. \end{array}$$

##### Glutamate

We modeled the combined effect of glutamine (*Gln*) uptake and deamination to produce glutamate. We assumed glutamine uptake is saturated by extracellular glutamine and hence modeled it as a hyperbolic function. We assumed deamination of glutamine to glutamate is quick and thus represents the dominant process consuming intracellular glutamine. Accordingly, we combined uptake and deamination into a single production term for glutamate. We considered glutamate consumption by cystine uptake through SLC7A11, GSH synthesis, and general consumption.

$$\begin{array}{ll} \frac{d{\left[\mathrm{Glu}\right]}_{\left(t\right)}}{\mathrm{dt}}= & \;k_{Glu\_\text{Production}}\frac{\left[\mathrm{Gln}\right]}{K_{Gln\_\text{Import}}+\left[\mathrm{Gln}\right]}\;‐\;k_{Glu\_\text{Degradation}}{\left[\mathrm{Glu}\right]}_{\left(t\right)}\\ & \;‐k_{\text{GSH}\_\text{Production}}{\left[\mathrm{Cys}\right]}_{\left(t\right)}{\left[\mathrm{Glu}\right]}_{\left(t\right)}\\ & \;‐k_{Cys_{2}\_\text{Import}}\frac{{\left[Cys_{2}\right]}_{\text{External}}}{K_{Cys_{2}\_\text{Import}}+{\left[Cys_{2}\right]}_{\text{External}}}\left[SLC7A11\right]{\left[\mathrm{Glu}\right]}_{\left(t\right)}. \end{array}$$

##### NADPH

We assumed total NADP^+^/H to be constant, given that production of NADP^+^ from NAD+ by NADK is much slower compared to NADPH regeneration (Fan *et al*, 2014; Liu *et al*, 2018, 2020). Consequently, we were able to express NADP^+^ in terms of total NADP^+^/H and NADPH. We considered NADPH regeneration from glucose and glutamate metabolism. Glucose feeds into the pentose phosphate pathway and regenerates NADPH from NADP^+^ through G6PD and PGD. Both glucose and glutamate feed into and overflow from the TCA cycle to regenerate NADPH by fluxing through IDH1 and ME1. G6PD, IDH1, and ME1 are the dominant enzymes that regenerate NADPH (Chen *et al*, 2019). Hence, we modeled the contribution to NADPH by glucose and glutamate additively. Of note, the contribution to NADPH by glucose was modeled as a hyperbolic function of extracellular glucose, given the combined effect of glucose transporter saturation and the metabolic reactions of the pentose phosphate pathway. We considered three routes of consumption for NADPH, namely reduction of cystine, reduction of GSSG, and anabolism such as fatty acid synthesis (Fan *et al*, 2014). We modeled anabolic NADPH consumption as a first‐order reaction.

$$\begin{array}{ll} \frac{d{\left[\text{NADPH}\right]}_{\left(t\right)}}{\mathrm{dt}}\;= & \;k_{{\text{NADP}}^{+}\_\text{Reduction}\_Glc}\frac{\left[\mathrm{Glc}\right]}{K_{Glc\_\text{Import}}+\left[\mathrm{Glc}\right]}\left({\text{NADP}}_{\text{Total}}‐{\left[\text{NADPH}\right]}_{\left(t\right)}\right)\left({\text{1 + switch}}_{{\text{NADP}}^{+}\_\text{Import}}\right)\\ & \;+\;k_{{\text{NADP}}^{+}\_\text{Reduction}\_Glu}\left[Glu\right]\left({\text{NADP}}_{\text{Total}}‐{\left[\text{NADPH}\right]}_{\left(t\right)}\right)\left(1‐{\text{switch}}_{{\text{NADP}}^{+}\_\text{Import}}\right)‐\;k_{\text{NADPH}\_\text{Oxidation}\_\text{Anabolism}}{\left[\text{NADPH}\right]}_{\left(t\right)}‐\;k_{\text{NADPH}\_\text{Oxidation}\_Cys_{2}}{\left[Cys_{2}\right]}_{\left(t\right)}{\left[\text{NADPH}\right]}_{\left(t\right)}\\ & ‐k_{\text{NADPH}\_\text{Oxidation}\_\text{GSSG}}{\left[\text{GSSG}\right]}_{\left(t\right)}{\left[\text{NADPH}\right]}_{\left(t\right)}. \end{array}$$

We inserted the switch terms, $‐1\leq {\mathrm{switch}}_{{\mathrm{NADP}}^{+}\_\mathrm{Import}}\leq 1$, as an artificial handle to model the allocation of reducing power that reduces NADP^+^ coming from the nutrients. This allowed us to model the cellular dependence on glucose as the primary source of NADP^+^ reduction (Fig EV4B).

##### Reduced glutathione

We modeled GSH production from reducing GSSG and *de novo* synthesis, and GSH removal by oxidation and general degradation. We note that removal of one molecule of ROS consumes two molecules of GSH, thus representing a third‐order reaction. We assumed the same rate of general, first‐order degradation for both GSH and GSSG.

$$\begin{array}{ll} \frac{d{\left[\text{GSH}\right]}_{\left(t\right)}}{\mathrm{dt}}\;= & \;2k_{\text{GSH}\_\text{Reduction}}{\left[\text{NADPH}\right]}_{\left(t\right)}{\left[\text{GSSG}\right]}_{\left(t\right)}+\;k_{\text{GSH}\_\text{Synthesis}}{\left[\mathrm{Cys}\right]}_{\left(t\right)}{\left[\mathrm{Glu}\right]}_{\left(t\right)}‐2k_{\text{GSH}\_\text{Oxidation}}{\left[\text{ROS}\right]}_{\left(t\right)}{\left[\text{GSH}\right]}_{\left(t\right)}^{2}‐\;k_{\text{GSH}\_\text{Degradation}}{\left[\text{GSH}\right]}_{\left(t\right)}. \end{array}$$

##### Oxidized glutathione

We modeled GSSG production from oxidizing GSH, and GSSG removal by reduction and general first‐order degradation.

$$\begin{array}{ll} \frac{d{\left[\text{GSSG}\right]}_{\left(t\right)}}{\mathrm{dt}}\;= & \;k_{\text{GSH}\_\text{Oxidation}}{\left[\text{ROS}\right]}_{\left(t\right)}{\left[\text{GSH}\right]}_{\left(t\right)}^{2}\\ & \;‐k_{\text{GSH}\_\text{Reduction}}{\left[\text{NADPH}\right]}_{\left(t\right)}{\left[\text{GSSG}\right]}_{\left(t\right)}‐\;k_{\text{GSH}\_\text{Degradation}}{\left[\text{GSSG}\right]}_{\left(t\right)}. \end{array}$$

##### Reactive oxygen species

We modeled ROS production additively from glucose metabolism, glutamate metabolism, and other processes. In particular, we modeled the production of ROS from glucose metabolism as a hyperbolic function of extracellular glucose, representing the effect of glucose transporter saturation and the combined rates of glycolysis, anaplerosis of TCA cycle intermediates, and OXPHOS. We note that basal ROS production can be interpreted in different ways, such as mitochondrial ROS production arising from various anaplerotic reactions to the TCA cycle, ROS production from NADPH oxidases or, more broadly, processes that are not directly dependent upon glucose and glutamate metabolism. We modeled ROS regulation by NOX‐mediated tyrosine kinase and Ca^2+^ signaling multiplicatively given that signaling processes are more likely to regulate the speed of underlying metabolic processes instead of switching on or off certain ROS‐producing processes. We modeled ROS autoinhibition as a Hill function. Degradation of ROS by GSH was modeled as a third‐order reaction, since two molecules of GSH are consumed by one molecule of ROS.

$$\begin{array}{ll} \frac{d{\left[\text{ROS}\right]}_{\left(t\right)}}{\mathrm{dt}}= & \;{\text{ROS}}_{\text{Production\_Partition}}{\left[{\text{Ca}}^{2+}\right]}_{\left(t\right)}{\left[{\text{TK}}_{\text{phos}}\right]}_{\left(t\right)}\frac{K_{\text{ROS}\_\text{Inhibition}}^{n1}\;}{K_{\text{ROS}\_\text{Inhibition}}^{n1}+{\left[\mathrm{ROS}\right]}_{\left(t\right)}^{n1}}\\ & \;‐k_{\text{ROSDegradation}}{{\left[\text{GSH}\right]}^{2}}_{\left(t\right)}{{\left[\text{ROS}\right]}_{\left(t\right)}}^{.}\\ {\text{ROS}}_{\text{Production\_Partition}}= & \;k_{\text{ROS}\_\text{Production}\_\text{Basal}}\left(1‐f_{\text{ROS}\_\text{from}\_Glc}‐f_{\text{ROS}\_\text{from}\_Glu}\right)\\ & \;+k_{\text{ROS}\_\text{Production}\_\text{from}\_Glc}\frac{\left[\mathrm{Glc}\right]}{K_{Glc\_\text{Import}}+\left[\mathrm{Glc}\right]}f_{\text{ROS}\_\text{from}\_Glc}\\ & \;+k_{\text{ROS}\_\text{Production}\_\text{from}\_Glu}\frac{\left[\mathrm{Glu}\right]}{K_{Glu\_\text{Import}}+\left[\mathrm{Glu}\right]}f_{\text{ROS}\_\text{from}\_Glu}. \end{array}$$

Under SLC7A11 overexpression, the cells secrete more glutamate, potentially diverting it from entering the TCA cycle. The cells maintain the influx to TCA cycle through anaplerosis (Muir *et al,* 2017). To model this compensation to various degree and its consequence (Fig EV4C), we systematically altered the $k_{\text{ROS}\_\text{Production}\_\text{from}\_Glu}$. ROS contribution parameters $f_{\text{ROS}\_\text{from}\_Glc}$ and $f_{\text{ROS}\_\text{from}\_Glu}$ describe contribution to ROS by glucose and glutamate metabolism, respectively.

##### Calcium

We modeled basal influx of Ca^2+^ into the cytosol and ROS‐stimulated Ca^2+^ influx additively, and a general first‐order efflux of Ca^2+^.

$$\frac{d{\left[{\text{Ca}}^{2+}\right]}_{\left(t\right)}}{\mathrm{dt}}=k_{{\text{Ca}}^{2+}\_\text{Import}\_\text{Basal}}+k_{{\text{Ca}}^{2+}\_\text{Import}\_\text{ROS}}{\left[\text{ROS}\right]}_{\left(t\right)}‐k_{{\text{Ca}}^{2+}\_\text{Out}}{\left[{\text{Ca}}^{2+}\right]}_{\left(t\right)}.$$

##### Active, reduced tyrosine phosphatase

We considered reduced tyrosine phosphatase (*PPTase*) as the active species. We assumed a steady‐state expression level for total tyrosine phosphatase. Hence, we were able to express inactive, oxidized phosphatase in terms of total and reduced phosphatase. The tyrosine phosphatase PTP1B can be reduced by both glutaredoxin with GSH and by the thioredoxin/thioredoxin reductase system with NADPH (Barrett *et al*, 1999; Dagnell *et al*, 2017). In this study, we considered situations where NADPH is limited. Thus, as for ROS removal, we modeled GSH as the primary source of phosphatase reduction. Since one molecule of reduced active tyrosine phosatase has one reactive cysteine residue in its catalytic pocket, we assumed it is inactivated by one molecule of ROS, resulting in one molecule of oxidized tyrosine phosphatase and one molecule of a reactive radical species, representing a pseudo‐second‐order oxidation reaction. Of note, consumption of GSH and ROS by the phosphatase was negligible compared to destruction of GSH and ROS and, accordingly, it was excluded from the rate equations for GSH and ROS.

$$\begin{array}{ll} \frac{d{\left[PPTase_{\text{Reduced}}\right]}_{\left(t\right)}}{\mathrm{dt}}= & \;k_{PPTase\_\text{Reduction}\_\text{GSH}}{\left[\text{GSH}\right]}_{\left(t\right)}\left(PPTase_{\text{Total}}‐{\left[PPTase_{\text{Reduced}}\right]}_{\left(t\right)}\right)\\ & \;‐\;k_{PPTase\_\text{Oxidation}\_\text{ROS}}{\left[\text{ROS}\right]}_{\left(t\right)}{\left[PPTase_{\text{Reduced}}\right]}_{\left(t\right)}. \end{array}$$

##### Active, phosphorylated tyrosine kinases

We considered the phosphorylated tyrosine kinases (TK) as the active species. We assumed a steady‐state expression level for total TK. We expressed inactive, dephosphorylated TK in terms of total and phosphorylated TK. We assumed constant activation/phosphorylation of TK, so TK phosphorylation was modeled as a first‐order reaction.

$$\begin{array}{ll} \frac{d{\left[{\text{TK}}_{\text{Phos}}\right]}_{\left(t\right)}}{\mathrm{dt}}= & \;k_{\text{TK}\_\text{Phosphorylation}}\left({\text{TK}}_{\text{Total}}‐{\left[{\text{TK}}_{\text{Phos}}\right]}_{\left(t\right)}\right)\\ & \;‐\;k_{\text{TK}\_\text{Dephosphorylation}}{\left[PPTase_{\text{Reduced}}\right]}_{\left(t\right)}{\left[{\text{TK}}_{\text{Phos}}\right]}_{\left(t\right)}. \end{array}$$

#### Estimation of protein and metabolite concentrations

We gathered estimates or measurements of protein and metabolite concentrations in mammalian cells from various sources, including publications and databases (for the complete list, see Appendix Table S1). In general, the metabolites considered in this study have estimates in units of molarity (Appendix Table S1). Signaling proteins were generally expressed in the submicromolar range, so we used 100 nM as an estimate for both tyrosine kinase and phosphatase. In contrast, SLC7A11 concentration has not been determined previously in molarity, but this information was available in units of parts‐per‐million (ppm) from proteomic studies on PaxDb (Wang *et al*, 2012). To estimate the range of SLC7A11 concentration in molarity, we used actin as a reference since it is the best quantified protein in terms of both molarity and ppm, giving us an estimate of 0–10 µM SLC7A11 in human cells (Appendix Table S2).

#### Estimation of metabolic fluxes

We sourced measurements on metabolic fluxes from various publications. Generally, the flux measurements had been normalized to the number of cells used or the total amount of proteins in the lysate (see details in Appendix Table S4). Therefore, we assumed a general protein concentration (≈ 300 mg/ml) or volume (≈ 3 pl) for human cells and converted the fluxes into units of µM/min. For nutrient uptake, half‐maximal data were gathered from various publications (Appendix Table S3). We assumed the Michaelis parameter (*K*_M_) for all transporters to be constant.

#### Estimation of reaction constants

To estimate the reaction rate constants, we assumed that the estimates of fluxes, as well as those for the concentrations of metabolites and signaling proteins, represent steady‐state fluxes and concentrations in the cell. Lists of the concentration and flux estimates we used are presented in Appendix Tables S1, S4 and S5, respectively. First, we computed the rate constants for reactions in which the rate and the concentrations were known. By assuming the system at steady state, we were then able to compute the rate constants in equations where only one reaction rate was unknown (Appendix Table S8). That allowed us to compute the rate constants for the metabolism part of our model. For the signaling part (ROS feedback loops), the reaction rates are generally unknown for redox catastrophe. Consequently, instead of computing exact values from known reaction rates, we estimated the ratios of forward and reverse rate constants according to fold‐activation before and after redox catastrophe, assuming that the system shifted to another steady state (Appendix Table S6). In doing so, we constrained the relative value of the rate constants. Some artificial handles were inserted into the model to explore their effects, and their values were chosen arbitrarily (Appendix Table S7).

#### Simulation for steady‐state solutions and temporal dynamics

We implemented our model in Mathematica 12. For steady‐state solutions, we used the Solve[] function built into Mathematica. To simulate temporal dynamics upon perturbations, including nutrient starvation and disruptions to metabolic reaction rates, we used the built‐in DSolve[] function. We used the steady‐state solutions prior to the intended perturbation as the initial state for temporal dynamics. To confirm our simulation results, we independently implemented our systems in MATLAB (R2019b) and obtained the same results.

## Author contributions

J‐HH, HKCC, and S‐hC conceived the project. J‐HH and C‐CW conducted the computational and statistical analyses. HKCC, Y‐CL, and S‐hC designed and conducted the experiments. J‐HH, HKCC, and S‐hC wrote the manuscript.

## Conflict of interest

The authors declare that they have no conflict of interest.

## Supporting information



Appendix

Expanded View Figures PDF

## Data Availability

Mathematic code for our nutrient‐redox model is available at https://github.com/imb‐lcd/2021_redoxmodel.
