# Supplementary material for: Multistability maintains redox homeostasis in human cells
Source: Mol Syst Biol. 2021 Oct 6;17(10):e10480. doi: 10.15252/msb.202110480 (PMC8493564; doi:10.15252/msb.202110480)
Supplement: Supplementary file 1 — Appendix [file MSB-17-e10480-s002.pdf]

APPENDIX for "Multistability maintains redox homeostasis in human cells"

Jo-Hsi Huang[1,a], Hannah K. C. Co[2,a], Yi-Chen Lee[2], Chia-Chou Wu[2], Sheng-hong Chen[2,b]

1. Department of Chemical and Systems Biology, Stanford University School of Medicine, Stanford, CA 94305, USA
2. Institute of Molecular Biology, Academia Sinica, Taipei, Taiwan 115

a. These authors contributed equally
b. Corresponding author: shengchen@gate.sinica.edu.tw

TABLE OF CONTENTS

Appendix Supplementary Figures

Figure S1 --- page 2

Figure S2 --- page 3

Appendix Supplementary Tables

Table S1 --- page 4

Table S2 --- page 5

Table S3 --- page 6

Table S4 --- page 7

Table S5 --- page 8

Table S6 --- page 9

Table S7 --- page 10

Table S8 --- page 11

Table S9 --- page 12

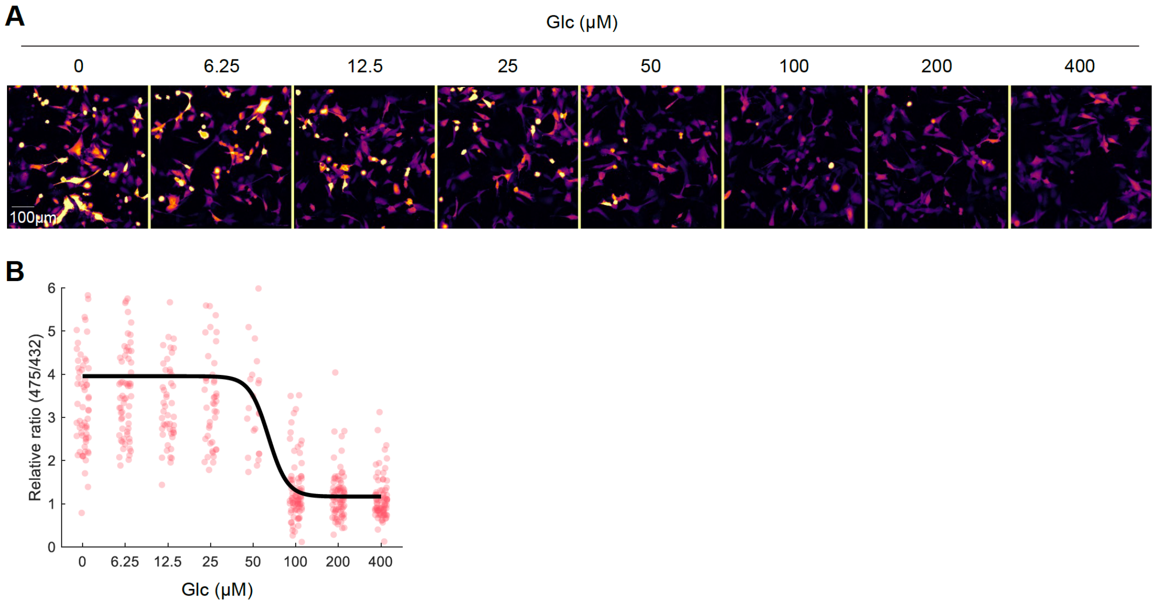

Appendix Figure S1. Ultrasensitivity of the ROS-response curve in U87-MG cells

(A) Representative HyPer7 images of U87-MG-HyPer7 cells 10 h after glucose deprivation (from 400 μM to 0 μM). (B) A dose-response curve of ROS 10 h after glucose deprivation. For each glucose concentration, the ROS levels of 80 cells were quantified. To deconvolute the effects of non-responsive cells, they were not considered in the analyses of the lower five glucose concentrations (0 μM, 6.3 μM, 12.5 μM, 25 μM and 50 μM). A Hill exponent (n) was fitted to be 6.5, with a 95% confidence interval of 2-11.

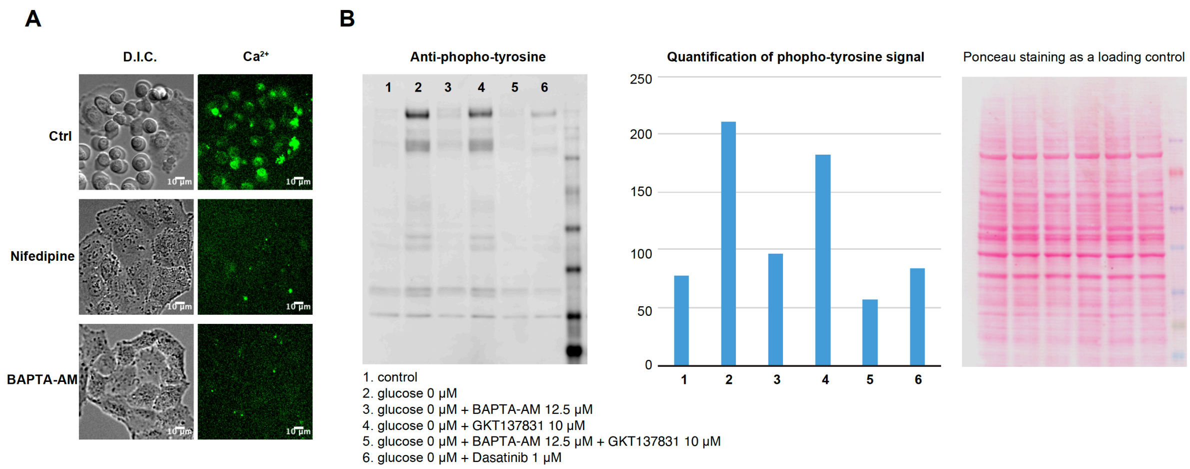

Appendix Figure S2. Validation of calcium and NOX inhibitors.

(A) Representative differential interference contrast (D.I.C., left panel) and fluorescent images of calcium indicator Fluo-4 AM (right panel) of LN18 cells 3 h after glucose deprivation. Cells were pre-treated with Nifedipine (10 μM) and BAPTA-AM (12.5 μM) 1 h before glucose deprivation to inhibit calcium signaling. (B) Activity of tyrosine kinase signaling was probed with western blot analysis using an anti-phospho-tyrosine antibody (4G10, Cell Signaling Technology) under normal [glucose] (band 1), no glucose (band 2), and no glucose with addition of BAPTA-AM alone (band 3), GKT137831 alone (band 4) and both (band 5). A multi-targeted tyrosine kinase inhibitor, Dasatinib (1 μM, band 6), was used as a positive control for suppression of tyrosine kinase signaling. The middle and right panels show the quantifications of phospho-tyrosine and the protein loading control respectively.

Appendix Table S1 - Variable Concentration Ranges

Molecule	Location	Organism	Concentration	Unit	Ref	Assumption made to convert unit	Converted quanty	Converted unit	Note
SLC7A11	Cell	Human	200	ppm	PaxDB (https://pax-db.org/protein/1846679); See table 002	10000 ppm ~ 500 uM (See Table EV2)	10	uM	Upper bound
GSH	Cell	Human	10	mM	Bionumbers #113865;<br>Forman et al. Mol. Aspects Med. 2009	NA	10000	uM	Upper bound
GSH	Cell	Human	1	mM	Bionumbers #113865;<br>Forman et al. Mol. Aspects Med. 2009	NA	1000	uM	Lower bound
GSH/GSSG	Cell	Human	100	NA	Zitka et al. Oncol. Lett. 2012	NA	100	NA	Upper bound
GSH/GSSG	Cell	Human	10	NA	Zitka et al. Oncol. Lett. 2012	NA	10	NA	Lower bound
GSSG	Cell	Human	1	mM	Calculated from above	NA	1000	uM	Upper bound
GSSG	Cell	Human	0.01	mM	Calculated from above	NA	10	uM	Lower bound
Glu	Cell	Human	20	mM	Bionumbers #100790	NA	20000	uM	NA
Glu	Cell	Human	10	mM	Featherstone. ACS Chem. Neurosci. 2010	NA	10000	uM	Upper bound
Glu	Cell	Human	5	mM	Featherstone. ACS Chem. Neurosci. 2010	NA	5000	uM	Lower bound
Cys2	Cell	Human	2	nmol / mg protein	Jamalpoor et al. Biomed. Chromatogr. 2017, Fig. 3	Cellular protein concentration 300 mg	600	uM	Cystinotic cell
Cys2	Cell	Human	0.05	nmol / mg protein	Jamalpoor et al. Biomed. Chromatogr. 2017, Fig. 3	Cellular protein concentration 300 mg	15	uM	Healthy cell
Cys	Cell	Human	1.5	nmol / mg protein	Sato et al. J. Biol. Chem. 2005, Fig. 7	Cellular protein concentration 300 mg	450	uM	NA
Cys	Cell	Human	4	nmol / mg protein	Stipanuk et al. J. Nutr. 2006, Fig. 4	Cellular protein concentration 300 mg	1200	uM	Lower bound
Cys	Cell	Human	6	nmol / mg protein	Stipanuk et al. J. Nutr. 2006, Fig. 4	Cellular protein concentration 300 mg	1800	uM	Upper bound
NADPH	Cell	Human	3	uM	Zou et al. Nat. Protoc. 2018, Table 2	NA	3	uM	NA
NADPH/NADP+	Cell	Human	300	NA	Zou et al. Nat. Protoc. 2018, Table 2	NA	300	NA	Upper bound
NADPH/NADP+	Cell	Human	15	NA	Zou et al. Nat. Protoc. 2018, Table 2	NA	15	NA	Lower bound
NADP+	Cell	Human	200	nM	Zou et al. Nat. Protoc. 2018, Table 2	NA	0.2	uM	Upper bound
NADP+	Cell	Human	10	nM	Zou et al. Nat. Protoc. 2018, Table 2	NA	0.01	uM	Lower bound
ROS	Cell	Human	0.01	uM	Giorgio et al. Nat.Rev. Mol. Cell Biol. 2007, Box 1	NA	0.01	uM	Proliferative
ROS	Cell	Human	0.1	uM	Giorgio et al. Nat.Rev. Mol. Cell Biol. 2007, Box 1	NA	0.1	uM	Proliferative
ROS	Cell	Human	1	uM	Giorgio et al. Nat.Rev. Mol. Cell Biol. 2007, Box 1	NA	1	uM	Growth arrest
ROS	Cell	Human	100	uM	Giorgio et al. Nat.Rev. Mol. Cell Biol. 2007, Box 1	NA	100	uM	Cell death
ROS	Cell	Human	1	uM	Antunes and Cadenas. Free Radic. Biol. Med. 2001, Fig. 2	NA	1	uM	Cell death
ROS	Cell	Human	50	nM	Huang et al. ACS Synth. Biol. 2016, Fig. 4 and 5	NA	0.5	uM	Cell death
ROS	Cell	Human	0.01	uM	Sies. Redox Biol. 2017;<br>Sies and Jones. Nat. Rev. Mol. Cell Biol. 2020	NA	0.01	uM	Proliferative
ROS	Cell	Human	0.1	uM	Sies. Redox Biol. 2017;<br>Sies and Jones. Nat. Rev. Mol. Cell Biol. 2020	NA	0.1	uM	Stress;
ROS	Cell	Human	1	uM	Sies. Redox Biol. 2017;<br>Sies and Jones. Nat. Rev. Mol. Cell Biol. 2020	NA	1	uM	Growth arrest; Cell death
RTK	Cell	Human	100	nM	Take generic number from Cell Biology by the Numbers (http://book.bionumbers.org/what-are-the-absolute-numbers-of-signaling-proteins/)	NA	0.1	uM	NA
PPTase	Cell	Human	100	nM	Take generic number from Cell Biology by the Numbers (http://book.bionumbers.org/what-are-the-absolute-numbers-of-signaling-proteins/)	NA	0.1	uM	NA
Ca2+	Cell	Human	100	nM	Bionumbers #107490;<br>Clapham. Cell 2007	NA	0.1	uM	NA
Glc	RPMI-1640	NA	11.11	mM	General RPMI-1640 recipe	NA	11110	uM	NA
Gln	RPMI-1640	NA	2	mM	General RPMI-1640 recipe	NA	2000	uM	NA
Cys2	RPMI-1640	NA	0.2	mM	General RPMI-1640 recipe	NA	200	uM	NA

Appendix Table S2 - Estimation of SLC7A11 using Actin

Molecule	Location	Organism	Concentration	Unit	Ref	Assumption made to convert unit	Converted quanty	Converted unit	Note
Total actin	Cell	Mammals	500	uM	Lodish et al. Freeman. 2000, Molecular Cell Biology, 4th Edition, Section 18.1	NA	500	uM	NA
Total actin	Cell	Acanthamoeba	200	uM	Pollard et al. Annu. Rev. Biophys. Biomol. Struct. 2000, Table1	NA	200	uM	NA
Total actin	Cell	Dictyostellium	250	uM	Pollard et al. Annu. Rev. Biophys. Biomol. Struct. 2000, Table1	NA	250	uM	NA
Total actin	Cell	Mammals (Neutrophils)	400	uM	Pollard et al. Annu. Rev. Biophys. Biomol. Struct. 2000, Table1	NA	400	uM	NA
Total actin	Cell	Mammals (Platelets)	550	uM	Pollard et al. Annu. Rev. Biophys. Biomol. Struct. 2000, Table1	NA	550	uM	NA
Polymerized actin	Cell	Eukaryotes	1000	uM	Bionumbers #109980	NA	1000	uM	NA
Total actin	Cell	Unspecified	100	uM	Bionumbers #109293	NA	100	uM	NA
Total G-actin	Cell	Unspecified	100	uM	Bionumbers #112131	NA	100	uM	NA
Total actin	Cell	Dictyostellium	250	uM	Bionumbers #109565	NA	250	uM	NA
ACTA1	Cell	Human	150	ppm	PaxDB (https://pax-db.org/protein/1854428)				normalized averge
ACTB	Cell	Human	5000	ppm	PaxDB (https://pax-db.org/protein/1853523)				normalized averge
ACTC1	Cell	Human	800	ppm	PaxDB (https://pax-db.org/protein/1847228)				normalized averge
ACTG1	Cell	Human	5000	ppm	PaxDB (https://pax-db.org/protein/1851354)				normalized averge
Total Actin	Cell	Human	10000	ppm	Summation over PaxDB entries	10000 ppm ~ 500 uM	500	uM	normalized averge
SLC7A11	Cell	Human	1.5	ppm	PaxDB (https://pax-db.org/protein/1846679)	10000 ppm ~ 500 uM	0.075	uM	normalized averge
SLC7A11	Cell	Human	200	ppm	PaxDB (https://pax-db.org/protein/1846679)	10000 ppm ~ 500 uM	10	uM	Upper bound
Total protein	Cell	Human	10 * 10^9	molecules	Cell biology by the numbers; http://book.bionumbers.org/how-many-proteins-are-in-a-cell/	Cell volume ~ 3 pL; Avogadro's number ~ 6 * 10^23	50	mM	

Appendix Table S3 - Nutrient Uptake Km

Nutrient	Transporter	Measure	Value	Unit	Ref	Assumption made to convert unit	Converted quanty	Converted unit	Note
Glc	SLC2A1	Km	1.5	mM	Lodish et al. Freeman. 2000, Molecular Cell Biology, 4th Edition, Section 15.3	NA	1500	uM	NA
Gln	SLC1A5	Km	0.1	mM	Pingitore et al. Biochim. Biophys. Acta 2013, Fig. 9A	NA	100	uM	NA
Gln	SLC1A5	Km	30	uM	Fuchs and Bode. Semin. Cancer Biol. 2005	NA	30	uM	Lower estimate
Gln	SLC1A5	Km	90	uM	Fuchs and Bode. Semin. Cancer Biol. 2005	NA	90	uM	Higher estimate
Gln	SLC7A5	Km	1.6	mM	Fuchs and Bode. Semin. Cancer Biol. 2005	NA	1600	uM	NA
Gln	SLC38A2	Km	2.3	mM	Menchini and Chaudhry. Neuropharmacol. 2019	NA	2300	uM	NA
Cys2	SLC7A11	Km	0.05	mM	Thomas et al. PLOS ONE 2015	NA	50	uM	NA
Glu	SLC7A11	Km	7.5	mM	Thomas et al. PLOS ONE 2015	NA	7500	uM	Export Km for intracellular Glu

Appendix Table S4 - Flux Ranges

Process	Value	Unit	Ref	Assumption made to convert unit	Converted quanty	Converted unit	Note
Cys2 uptake	0.2	nmol / mg protein / min	Thomas et al. PLOS ONE 2015, Fig. 6	Cellular protein concentration 300 mg / mL	60	uM / min	Lower measurement
Cys2 uptake	0.8	nmol / mg protein / min	Thomas et al. PLOS ONE 2015, Fig. 6	Cellular protein concentration 300 mg / mL	240	uM / min	Higher measurement
Cys2 uptake	0.6	nmol / mg protein / min	Ye et al. J. Neurosci. 1999, Fig. 5D	Cellular protein concentration 300 mg / mL	180	uM / min	NA
Cys2 uptake	7	fmol / cell / hr	Hosios et al. Dev. Cell 2016, Fig. 1	Cell volume 3 pL	40	uM / min	NA
Gln uptake	3.5	pmol / ug protein / min	Parker et al. Metab. Eng. 2017, Fig. 2	Cellular protein concentration 300 ug / uL	1000	uM / min	NA
Gln uptake	100	fmol / cell / hr	Hosios et al. Dev. Cell 2016, Fig. 1	Cell volume 3 pL	1700	uM / min	NA
Gln uptake	300	fmol / cell / hr	Jain et al. Science 2012, Database S1	Cell volume 3 pL	5000	uM / min	Max from 60 cell lines
Gln uptake	70	fmol / cell / hr	Jain et al. Science 2012, Database S1	Cell volume 3 pL	1200	uM / min	Median from 60 cell lines
Gln uptake	10	fmol / cell / hr	Jain et al. Science 2012, Database S1	Cell volume 3 pL	170	uM / min	Min from 60 cell lines
Glu export	2	pmol / ug protein / min	Parker et al. Metab. Eng. 2017, Fig. 2	Cellular protein concentration 300 ug / uL	600	uM / min	NA
Glu export	20	fmol / cell / hr	Hosios et al. Dev. Cell 2016, Fig. 1	Cell volume 3 pL	100	uM / min	NA
Glu export	100	fmol / cell / hr	Jain et al. Science 2012, Database S1	Cell volume 3 pL	500	uM / min	Max from 60 cell lines
Glu export	10	fmol / cell / hr	Jain et al. Science 2012, Database S1	Cell volume 3 pL	50	uM / min	Median from 60 cell lines
Glu export	-3	fmol / cell / hr	Jain et al. Science 2012, Database S1	Cell volume 3 pL	-15	uM / min	Min from 60 cell lines; Net uptake instead of export
Glu anaplerosis	1	pmol / ug protein / min	Parker et al. Metab. Eng. 2017, Fig. 2	Cellular protein concentration 300 ug / uL	300	uM / min	NA
Glu to other metabolites	0.5	pmol / ug protein / min	Parker et al. Metab. Eng. 2017, Fig. 2	Cellular protein concentration 300 ug / uL	150	uM / min	Flux unaccounted for in the diagram
Gln to other metabolites / total labeling	0.1	NA	Hosios et al. Dev. Cell 2016, Fig. 1	Gln uptake flux 1700 uM / min (Hosios et al. Dev. Cell 2016)	170	uM / min	Gln channeled to soluble, polar fraction (metabolites)
Glc uptake	900	fmol / cell / hr	Jain et al. Science 2012, Database S1	Cell volume 3 pL	15000	uM / min	Max from 60 cell lines
Glc uptake	300	fmol / cell / hr	Jain et al. Science 2012, Database S1	Cell volume 3 pL	5000	uM / min	Median from 60 cell lines
Glc uptake	40	fmol / cell / hr	Jain et al. Science 2012, Database S1	Cell volume 3 pL	700	uM / min	Min from 60 cell lines
Glc uptake	300	fmol / cell / hr	Hosios et al. Dev. Cell 2016, Fig. 1	Cell volume 3 pL	5000	uM / min	NA
Glc uptake	40	pmol / ug protein / min	Parker et al. Metab. Eng. 2017, Fig. 2	Cellular protein concentration 300 ug / uL	12000	uM / min	NA
Glc uptake	0.5	umol / uL packed cell / hr	Tanner et al. Cell Syst. 2018, Fig. 1	70 ug / uL packed cells (Fan et al. Nature 2014, Fig. S9); Cellular protein concentration 300 ug / uL	36000	uM / min	Basal rate
Glc uptake	1	umol / uL packed cell / hr	Tanner et al. Cell Syst. 2018, Fig. 1	70 ug / uL packed cells (Fan et al. Nature 2014, Fig. S9); Cellular protein concentration 300 ug / uL	72000	uM / min	Ras-stimulated rate
Glc uptake	1.25	pmol / cell / hr	Liu et al. Nat. Cell Biol. 2020, Fig. S1	Cell volume 3 pL	7000	uM / min	NA
NADPH synthesis by NADK	12	pmol / 10^6 cell / hr	Liu et al. Cell Metab. 2018, Fig. 1 (in text)	Cell volume 3 pL	0.07	uM / min	Very slow compared to regeneration; Assume to be total NADPH/+ to be constant
OXPPP flux	2	nmol / uL packed cell / hr	Fan et al. Nature 2014, Fig. 1	70 ug / uL packed cells (Fan et al. Nature 2014, Fig. S9); Cellular protein concentration 300 ug / uL	150	uM / min	NA
OXPPP flux / glycolysis flux	0.04	NA	Liu et al. Nat. Cell Biol. 2020, Fig. 1d	Glc uptake flux 7000 uM / min (Liu et al. Nat. Cell Biol. 2020)	280	uM / min	NA
OXPPP flux / glycolysis flux	0.02	NA	Bionumbers #112684	NA	NA	NA	NA
NADPH regeneration by OXPPP	2	NADPH / Glc	Generic;1 from G6PD, 1 from PGD	NA	NA	NA	NA
NADPH regeneration total	10	nmol / uL packed cell / hr	Fan et al. Nature 2014, Fig. 1	70 ug / uL packed cells (Fan et al. Nature 2014, Fig. S9); Cellular protein concentration 300 ug / uL	720	uM / min	NA
Fraction NADPH consumed by anabolism	0.9	NA	Fan et al. Nature 2014, Fig. 4	NA	NA	NA	Upper bound
Fraction NADPH consumed by anabolism	0.7	NA	Fan et al. Nature 2014, Fig. 4	NA	NA	NA	Lower bound

Appendix Table S5 - Fluxes Used To Estimate Parameters

Process	Flux value used	Flux unit	Model	Note	Interpretation	Parameter estimated	Parameter unit
NADPH regeneration by Glc metabolism	500	uM / min	kNadphRedGlc * glc / (KglcIn + glc) * (nadptot - nadph) * (1 + switchNadphRed)	Assume total flux = 1000 uM / min;<br>Assume switchNadphRed = 0 for estimation process	Not addicted to Glc for NADPH	kNadphRedGlc	/ min
NADPH regeneration by Glu metabolism	500	uM / min	kNadphRedGlu * glu * (nadptot - nadph) * (1 - switchNadphRed)	Assume total flux = 1000 uM / min;<br>Assume switchNadphRed = 0 for estimation process	Not addicted to Glc for NADPH	kNadphRedGlu	/ uM / min
Cys2 uptake	30	uM / min	kCys2Im * cys2o / (Kcys2In + cys2o) * xct * glu	Assume xct = 0.1 uM;<br>Assume Cys2 uptake flux = 30 uM/ min;<br>Both lower estimates;<br>Assume 1:1 exchange;<br>Same as Glu export flux	Not addicted to Cys2 for Cys	kCys2Im; kGluEx	/ uM / min
Glu export	30	uM / min	kGluEx * cys2o / (Kcys2In + cys2o) * xct * glu	Assume xct = 0.1 uM;<br>Assume Glu export flux = 30 uM/ min;<br>Both lower estimates;<br>Assume 1:1 exchange;<br>Same as Glu export flux	Not addicted to Cys2 for Cys	kGluEx; kCys2Im	/ uM / min
Cys2 reduction by NADPH	30	uM / min	kCys2Red * cys2 * nadph	Assume all uptaken Cys2 gets reduced to Cys;<br>Assume NADPH provides all reducing power needed;<br>Same as NADPH consumed by Cys2		kCys2Red; kNadphOxCys2	/ uM / min
NADPH consumed by Cys2	30	uM / min	kNadphOxCys2 * cys2 * nadph	Assume all uptaken Cys2 gets reduced to Cys;<br>Assume NADPH provides all reducing power needed;<br>Same as Cys2 reduction by NADPH		kNadphOxCys2; kCys2Red	/ uM / min
NADPH consumed by anabolism	800	uM / min	kNadphOxAnab * nadph	Assume anabolism consumes 80% NADPH			/ min
Gln uptake and Glu production	300	uM / min	kGluPro * glu / (KglnIn + glu)	Assume all Gln uptaken hydrolyzed to Glu;<br>Assume Gln uptake = 300 uM / min;<br>A lower estimate	Not addicted to Gln	kGluPro	uM / min
GSH synthesis	30	uM / min	kGshPro * cys * glu	Assume 10% of Glu produced goes to GSH;<br>An upper bound estimate		kGshPro	/ uM / min

Appendix Table S6 - Variable Steady States to Estimate Parameters

Molecule	State	Value	Unit	Note
ROS	Full nutrient	0.01	uM	
GSH	Full nutrient	1000	uM	
GSSG	Full nutrient	100	uM	
NADPH	Full nutrient	1	uM	
Cys2	Full nutrient	1	uM	Lower estimate;Consistent with lower SLC7A11 used
Cys	Full nutrient	100	uM	Lower estimate;Consistent with lower SLC7A11 used
Glu	Full nutrient	10000	uM	
Ca2+	Full nutrient	0.1	uM	
RTK-phospho / RTK-total	Full nutrient	0.1	NA	Ref. Graham et al. Mol. Syst. Biol. 2012
RTK-phospho / RTK-total	Glc deprivation	1	NA	Ref. Graham et al. Mol. Syst. Biol. 2012
PPTase-reduced / PPTase-total	Full nutrient	1	NA	Ref. Graham et al. Mol. Syst. Biol. 2012
PPTase-reduced / PPTase-total	Glc deprivation	0.5	NA	Ref. Graham et al. Mol. Syst. Biol. 2012

Appendix Table S7 - Predetermined or Arbitrary Parameters

Parameter	Type	Value	Range	Unit	Note
xct	Constant	0.1	0 to 10	uM	Model low SLC7A11 cell
n1	Hill exponent	5	1 to n	NA	Ultrasensitivity in auto-inhibition
fRosGlc	Artificial handle	0.1	0 to 1	NA	Arbitrary
fRosGlu	Artificial handle	0.4	0 to 1	NA	Arbitrary
switchNadphRed	Artificial handle	0	-1 to 1	NA	Model non-addicted cell
foldRos	Artificial handle	1	0 to n	NA	Base line

Appendix Table S8 - Estimate Parameter Values

Estimation step	Process	Flux value used	Flux unit	Model	Known parameters and estimated steady stat	Parameter estimated	Estimated value	Parameter unit
1	NADPH regeneration by Glc metabolism	500	uM / min	kNadphRedGlc * glc / (Kglcln + glc) * (nadptot - nadph) * (1 + switchNadphRed)	glc = 10000 uM;<br>Kglcln = 1500 uM;<br>nadptot = 1 uM;<br>nadph = 0.1 uM;<br>switchNadphRed = 0;	kNadphRedGlc	5750	/ min
2	NADPH regeneration by Glu metabolism	500	uM / min	kNadphRedGlu * glu * (nadptot - nadph) * (1 - switchNadphRed)	glu = 10000 uM;<br>nadptot = 1 uM;<br>nadph = 0.9 uM;<br>switchNadphRed = 0;	kNadphRedGlu	0.5	/ uM / min
3	NADPH consumed by anabolism	800	uM / min	kNadphOxAnab * nadph	nadph = 1 uM;	kNadphOxAnab	800	/ min
4	Cys2 uptake	30	uM / min	kCys2Im * cys2o / (Kcys2In + cys2o) * xct * glu	cys2o = 0.2 uM;<br>Kcys2In = 0.05 uM;<br>xct = 0.1 uM;<br>glu = 10000 uM;	kCys2Im; kGluEx	0.0375	/ uM / min
4	Glu export	30	uM / min	kGluEx * cys2o / (Kcys2In + cys2o) * xct * glu	cys2o = 0.2 uM;<br>Kcys2In = 0.05 uM;<br>xct = 0.1 uM;<br>glu = 10000 uM;	kGluEx; kCys2Im	0.0375	/ uM / min
5	Cys2 reduction by NADPH	30	uM / min	kCys2Red * cys2 * nadph	cys2 = 1 uM;<br>nadph = 1 uM;	kCys2Red; kNadphOxCys2	30	/ uM / min
5	NADPH consumed by Cys2	30	uM / min	kNadphOxCys2 * cys2 * nadph	cys2 = 1 uM;<br>nadph = 1 uM;	kNadphOxCys2; kCys2Red	30	/ uM / min
5	Cys production from Cys2 and NADPH	60	uM / min	2 * kNadphOxCys2 * cys2 * nadph	cys2 = 1 uM;<br>nadph = 1 uM;	kNadphOxCys2; kCys2Red	30	/ uM / min
6	NADPH consumed by GSSG	170	uM / min	kNadphOxGssg * gssg * nadph	gssg = 100 uM;<br>nadph = 1 uM;	kNadphOxGssg	1.7	/ uM / min
6	GSSG reduced by NADPH	170	uM / min	kGshRed * nadph * gssg	gssg = 100 uM;<br>nadph = 1 uM;	kGshRed	1.7	/ uM / min
6	GSH regenerated by NADPH	340	uM / min	2 * kGshRed * nadph * gssg	gssg = 100 uM;<br>nadph = 1 uM;	kGshRed	1.7	/ uM / min
7	Gln uptake and Glu production	300	uM / min	kGluPro * gln / (KglnIn + gln)	gln = 2000 uM;<br>KglnIn = 100 uM;	kGluPro	315	uM / min
8	GSH synthesis	30	uM / min	kGshPro * cys * glu	cys = 100 uM;<br>glu = 10000 uM;	kGshPro	0.00005	/ uM / min
9	Cys degradation through other processes	30	uM / min	kCysDeg * cys	cys = 100 uM;	kCysDeg	0.3	/ min
10	Glu degradation through other processes	240	uM / min	kGluDeg * glu	glu = 10000 uM;	kGluDeg	0.024	/ min
11	GSH and GSSG degradation	30	uM / min	kGshDeg * gsh + kGssgDeg * gssg	gsh + gssg = 1000 uM;	kGshDeg; kGssgDeg	0.027	/ min
12	GSH oxidation by ROS	340	uM / min	2 * kGshOx * ros * gsh^2	ros = 0.01 uM;<br>gsh = 1000 uM;	kGshOx; kRosDeg	0.017	/ uM / min
12	GSSG production from GSH oxidation	170	uM / min	kGshOx * ros * gsh^2	ros = 0.01 uM;<br>gsh = 1000 uM;	kGshOx; kRosDeg	0.017	/ uM / min
12	ROS removed by GSH	170	uM / min	kRosDeg * ros * gsh^2	ros = 0.01 uM;<br>gsh = 1000 uM;	kGshOx; kRosDeg	0.017	/ uM / min
13	ROS produced by lipid metabolism	170	uM / min	foldRos * kRosProBase * (1 - fRosGlc - fRosGlu) * ca * rtkPhos * KrosInhRos^n1 / (KrosInhRos^n1 + ros^n1)	foldRos = 1;<br>fRosGlc = 0;<br>fRosGlu = 0;<br>ca = 0.1 uM;<br>rtkPhos = 0.01 uM;<br>KrosInhRos = 0.5;<br>n1 = 5;	kRosProBase	170000	/ uM / min
14	ROS produced by Glc metabolism	170	uM / min	foldRos * kRosProGlc * glc / (Kglcln + glc) * fRosGlc * ca * rtkPhos * KrosInhRos^n1 / (KrosInhRos^n1 + ros^n1)	foldRos = 1;<br>fRosGlc = 1;<br>fRosGlu = 0;<br>glc = 10000 uM;<br>Kglcln = 1500 uM;<br>ca = 0.1 uM;<br>rtkPhos = 0.01 uM;<br>KrosInhRos = 0.5;<br>n1 = 5;	kRosProGlc	195500	/ uM / min
15	ROS produced by Glu metabolism	170	uM / min	foldRos * kRosProGlu * glu * fRosGlu * ca * rtkPhos * KrosInhRos^n1 / (KrosInhRos^n1 + ros^n1)	foldRos = 1;<br>fRosGlc = 0;<br>fRosGlu = 1;<br>glu = 10000 uM;<br>ca = 0.1 uM;<br>rtkPhos = 0.01 uM;<br>KrosInhRos = 0.5;<br>n1 = 5;	kRosProGlu	17	/ uM^2 / min

Appendix Table S9 - Parameter Summary

Parameter	Type	Process	Interpretation	Note	Value
kRosDeg	Rate constant	ROS removal; GSH oxidation	ROS removal by GSH	Same as kGshOx	0.017
kGshOx	Rate constant	ROS removal; GSH oxidation	GSH consumed by ROS	Same as kRosDeg	0.017
kGshRed	Rate constant	GSH regeneration; NADPH oxidation	GSH regenerated by NADPH	Same as kNadphOxGssg	1.7
kNadphOxAnab	Rate constant	Anabolism; NADPH oxidation	NADPH consumed in anabolism		800
kNadphOxGssg	Rate constant	GSH regeneration; NADPH oxidation	NADPH consumed by GSSG	Same as kGshRed	1.7
kNadphOxCys2	Rate constant	Cys production; NADPH oxidation	NADPH consumed by Cys2	Same as kCys2Red	30
kCys2Red	Rate constant	Cys production; NADPH oxidation	Cys2 reduced by NADPH	Same as kNadphOxCys2	30
kPptaseRedGsh	Rate constant	RTK signaling	PPTase reduced by GSH		0.1
kPptaseOxRos	Rate constant	RTK signaling	PPTase oxidized by ROS		10
kRtkPhos	Rate constant	RTK signaling	RTK got phosphorylated		0.1
kRtkDephos	Rate constant	RTK signaling	RTK dephosphorylated by PPTase		10
kRosProBase	Rate constant	ROS production	Basal ROS production; ROS production by lipid metabolism; ROS production not (instantly) responsive to Glc or Glu		170000
kRosProGlc	Rate constant	ROS production	ROS production by Glc metabolism; ROS production responsive to Glc		195500
kRosProGlu	Rate constant	ROS production	ROS production by Glu metabolism; ROS production responsive to Glu		17
kNadpRedGlc	Rate constant	NADPH regeneration	NADPH regenerated by Glc metabolism; NADPH regenerated by OXPPP		5750
kNadpRedGlu	Rate constant	NADPH regeneration	NADPH regenerated by Glu metabolism; NADPH regenerated by IDH1 and ME1		0.5
kGluPro	Rate constant	Gln uptake; Glu production	Gln uptake; Glu production from Gln		315
kGluDeg	Rate constant	Glu metabolism	Glu consumed in other processes		0.024
kGluEx	Rate constant	Glu export; Cys2 uptake	Glu exported in exchange of Cys2	Same as kCys2Im	0.0375
kGshPro	Rate constant	GSH synthesis	GSH de novo synthesis		0.00005
kGshDeg	Rate constant	GSH degradation	GSH degradation; GSH export		0.027
kGssgDeg	Rate constant	GSSG degradation	GSSG degradation; GSSG export		0.027
kCysDeg	Rate constant	Cys metabolism	Cys consumed in other processes		0.3
kCaInBase	Rate constant	Ca2+ influx	Basal Ca2+ influx		0.1
kCaInRos	Rate constant	Ca2+ influx	ROS-induced Ca2+ release		1
kCaOut	Rate constant	Ca2+ efflux	Ca2+ efflux		1
kCys2Im	Rate constant	Glu export; Cys2 uptake	Cys2 uptake at the expense of Glu	Same as kGluEx	0.0375
xct	Constant	Glu export; Cys2 uptake	SLC7A11 abundance; Mass conservation		3
nadpTot	Mass conservation	NADPH regeneration	Total NADPH/+;Mass conservation		1
pptaseTot	Mass conservation	RTK signaling	Total PPTase; Mass conservation		0.1
rtkTot	Mass conservation	RTK signaling	Total RTK; Mass conservation		0.1
KrosInhRos	Km	ROS production	ROS auto-inhibition; Redox inhibition of OXPHOS / ETC enzymes; Oxidatie damages to ETC / mitocondria		0.5
n1	Hill exponent	ROS production	Ultrasensitivity in ROS auto-inhibition		5
glc	Constant	ROS production; NADPH regeneration	Glc abundance in media		10000
gln	Constant	ROS production; Glu production	Gln abundance in media		2000
cys2o	Constant	NADPH oxidation	Cys2 abundance in media		200
KglnIn	Km	Gln uptake	Gln concentration of half max uptake rate		100
Kcys2In	Km	Cys2 uptake	Cys2 concentration of half max uptake rate		50
KglcIn	Km	Glc uptake	Glc concentration of half max uptake rate		1500
fRosGlc	Artificial handle	ROS production	Fraction ROS production dependent on Glc metabolism		0.1
fRosGlu	Artificial handle	ROS production	Fraction ROS production dependent on Glu metabolism		0.4
switchNadphRed	Artificial handle	NADPH regeneration	Fraction NADPH regeneration dependent on Glc metabolism		0.7
foldRos	Artificial handle	ROS production	Strength of ROS production		1
