# Supplementary material for: Multistability maintains redox homeostasis in human cells
Source: Mol Syst Biol. 2021 Oct 6;17(10):e10480. doi: 10.15252/msb.202110480 (PMC8493564; doi:10.15252/msb.202110480)
Supplement: Supplementary file 2 — Expanded View Figures PDF [file MSB-17-e10480-s003.pdf]

Expanded View Figures

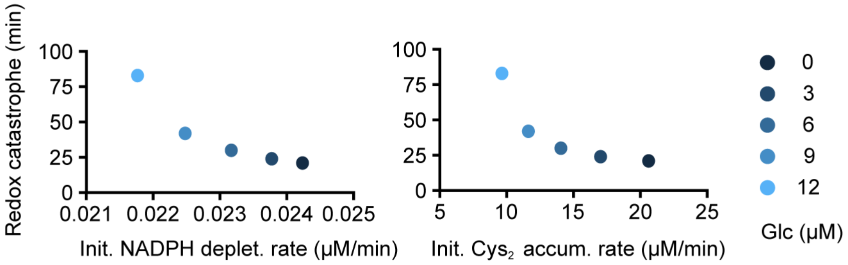

Figure EV1. Rates of NADPH depletion and cystine accumulation determine the timing of redox catastrophe.

Time to redox catastrophe as functions of the NADPH depletion rate (left panel) or the cystine accumulation rate (right) after changing from maximal glucose (10,000 μM) to low glucose (0–12 μM) *in silico*.

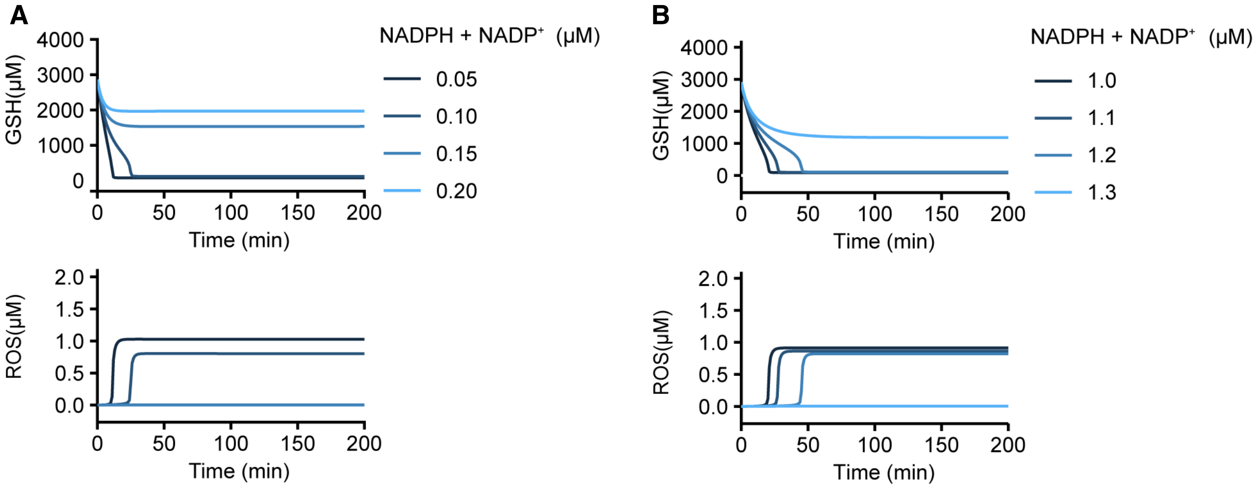

Figure EV2. Total NADPH concentration determines redox dynamics and steady state.

A Simulation of redox dynamics after a sudden decrease in total NADPH (NADPH + $NADP^+$) concentration. The steady-state metabolite concentrations (with 10,000 μM glucose and 1 μM total NADPH) were used as initial conditions for the simulation.

B Simulation of redox dynamics after glucose deprivation (from 10,000 to 0 μM) with varying total NADPH concentrations. Steady-state metabolite concentrations with varying total NADPH concentrations before glucose deprivation were used as initial conditions for the simulation.

 © 2021 The Authors

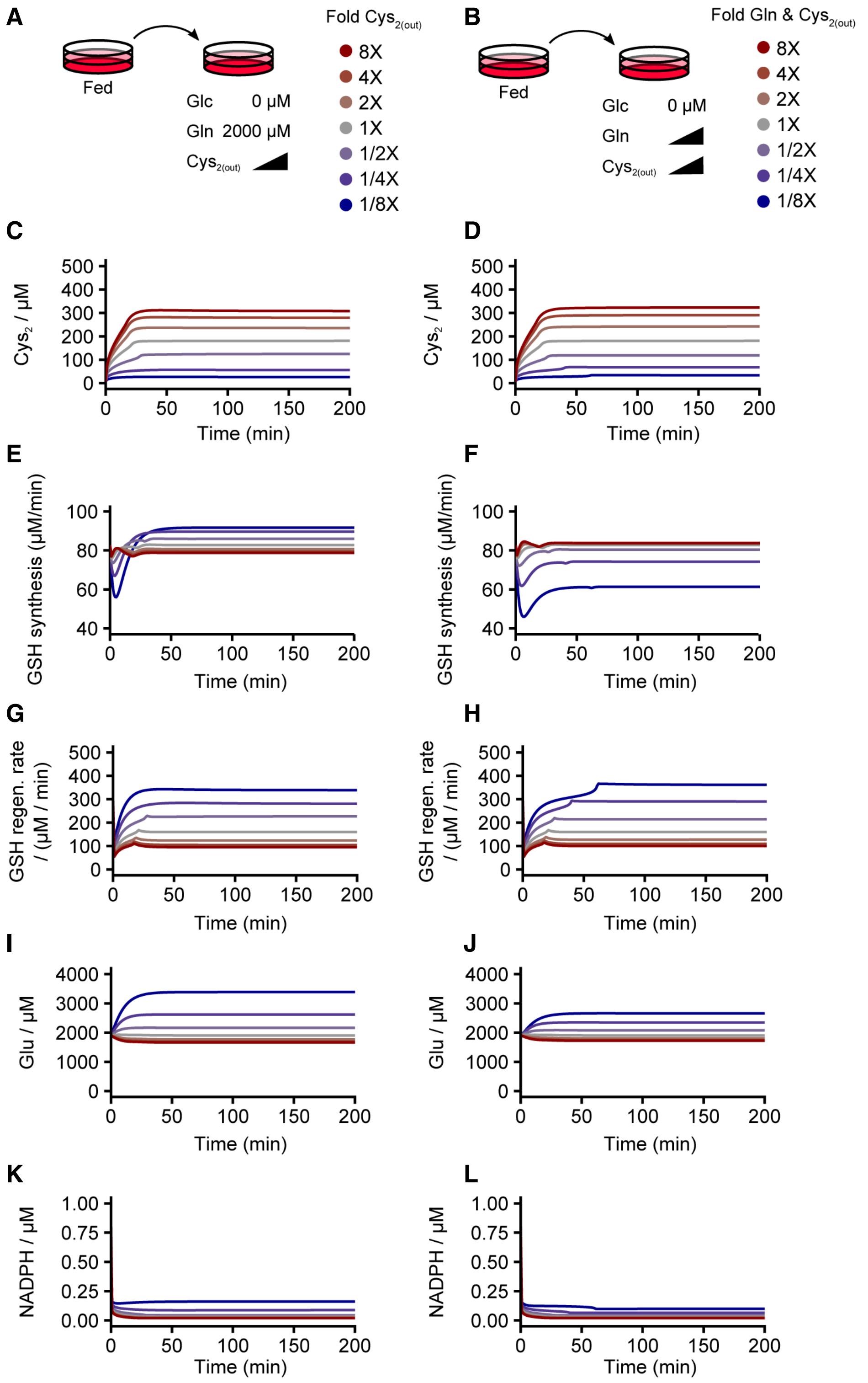

Figure EV3. Metabolite and redox dynamics after glucose deprivation coupled with titration of cystine or co-titration of cystine and glutamine.

A–L Metabolite and redox dynamics after glucose deprivation coupled with titration of cystine (A, C, E, G, I, K; left column) or co-titration of cystine and glutamine (B, D, F, H, J, L; right column). Steady-state cystine (C and D) and NADPH (K and L) concentrations and GSH regeneration rates (G and H) were comparable between cystine single-titration and cystine plus glutamine co-titration, whereas steady-state glucose concentrations (I and J) and the *de novo* GSH synthesis rate (E and F) differed.

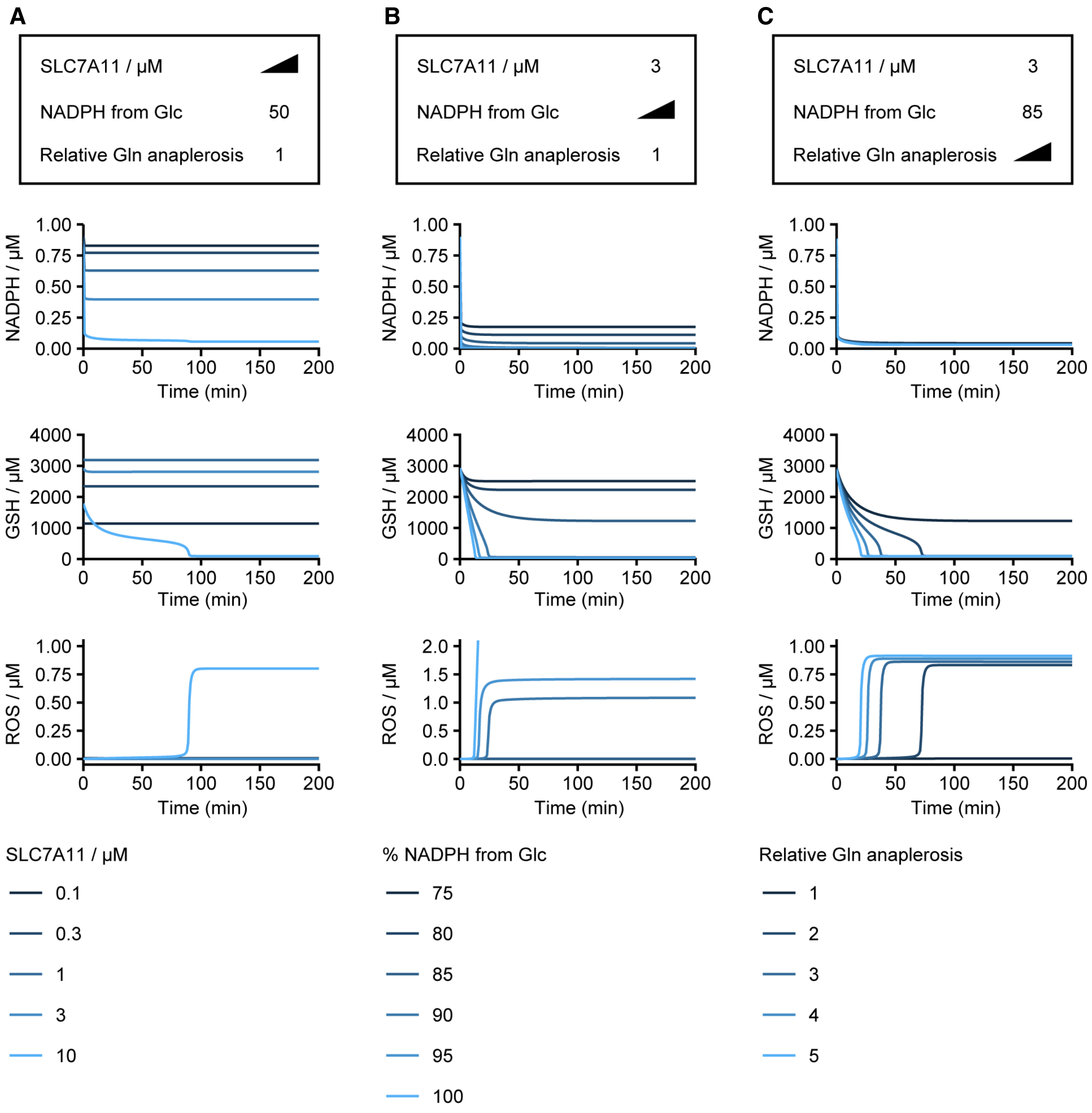

Figure EV4. Nutrient-redox model recapitulates *in silico* various susceptibility factors to glucose deprivation.

A–C Effects of *SLC7A11* expression level (A), dependence on glucose to regenerate NADPH (B), and upregulation of glutamine anaplerosis into TCA cycle/OXPHOS under conditions of high SLC7A11 expression (C) on redox dynamics upon glucose deprivation (from 10,000 to 0 µM). (A) Simulation with neutral dependence on glucose for NADPH regeneration and no upregulation of glutamine anaplerosis. Note that *SLC7A11* overexpression decreases intracellular glutamine through export, thereby limiting the contribution of glutamine to ROS production via OXPHOS in our model. (B) Simulation with varying glucose dependencies for NADPH under conditions of *SLC7A11* overexpression and no upregulation of glutamine anaplerosis. (C) Simulation with varying degrees of upregulated glutamine anaplerosis and *SLC7A11* overexpression and a dependency on glucose for NADPH regeneration. See Materials and Methods for *in silico* perturbation of glucose dependence of NADPH reduction (B) and strength of glutamine anaplerosis (C).

 © 2021 The Authors
