# Supplementary material for: Multistability maintains redox homeostasis in human cells
Source: Mol Syst Biol. 2021 Oct 6;17(10):e10480. doi: 10.15252/msb.202110480 (PMC8493564; doi:10.15252/msb.202110480)
Supplement: Supplementary file 3 [file MSB-17-e10480-s001.pdf]

molecular systems biology

Multistability maintains redox homeostasis in human cells

Jo-Hsi Huang, Hannah Co, Yi-Chen Lee, and Chia-Chou Wu, Sheng-hong Chen

DOI: 10.15252/msb.20211048

Corresponding author(s): Sheng-hong Chen (shengchen@gate.sinica.edu.tw)

Review Timeline:

Submission Date:	31st May 21
Editorial Decision:	1st Jul 21
Revision Received:	10th Aug 21
Editorial Decision:	31st Aug 21
Revision Received:	2nd Sep 21
Editorial Decision:	10th Sep 21
Revision Received:	11th Sep 21
Accepted:	14th Sep 21

Editor: Jingyi Hou

Transaction Report:

(Note: With the exception of the correction of typographical or spelling errors that could be a source of ambiguity, letters and reports are not edited. Depending on transfer agreements, referee reports obtained elsewhere may or may not be included in this compilation. Referee reports are anonymous unless the Referee chooses to sign their reports.)

1st Editorial Decision 1st Jul 2021

Thank you again for submitting your work to Molecular Systems Biology. We have now heard back from the three reviewers who agreed to evaluate your study. As you will see below, the reviewers raise substantial concerns about your work, which unfortunately preclude its publication in Molecular Systems Biology.

The reviewers acknowledge that the general topic of the study is interesting. However, they raised significant and overlapping concerns regarding the adequacy of the model, the focus on non-physiological glucose concentrations, the discrepancy between modeling results and experimental data, and the lack of experimental validation for some key predictions. As such, the reviewers express severe critiques with regard to the conclusiveness of the analyses. In particular, Reviewer #2 rated the technical quality, and the adequacy of method analysis and validation as "low/unacceptable", and Reviewer #3 rated the conceptual novelty and suitability of publication as "low".

Under these circumstances, and together with the fact that we only accept papers that receive enthusiastic support upon initial review, we see no other choice than to return the manuscript with the message that we cannot offer to publish it.

I am very sorry that the review of your work did not result in a more favorable outcome on this occasion. Still, I hope that you will not be discouraged from sending your work to Molecular

REFEREE REPORTS

Reviewer #1:

The manuscript by Huang et al. "Multistability maintains redox homeostasis in human cells" presents a nutrient-redox model that seeks to understand the system-level interplay between nutrient metabolic pathways (glucose, glutamine, cystine) and redox homeostasis. Using in silico modeling and experimental measurements, the authors show that ROS dynamics follow a switch-

like, bistable, hysteretic response to glucose deprivation. This manuscript builds on several recent publications demonstrating the role of cystine and the cystine/glutamate antiporter SLC7A11 in regulating cell death following glucose starvation. The paper is well written, and the results from the model nicely synthesize experimental findings from several groups on the role of SLC7A11, cystine, and glutamine in glucose addiction. This work fits squarely within the scope of MSB. Given the recent interest in cystine metabolism and its relevance to glucose starvation in cancer cells, this work will be a great interest to the cancer and redox metabolism communities, particularly the finding that bistability is a key mechanism required for redox homeostasis. However, there are several issues which the authors should address to improve the manuscript:

Major concerns:
- In Fig. 2C, the authors show a time course of NADPH and GSH levels where "Experimental measurements were adopted from (Joly et al, 2020)". In Fig. 3C of the original publication (PMID 31914417), the levels of NADPH were not detected at times greater than 30 min. It is therefore unclear how the authors here were able to show NADPH data at time points >30 min. In addition, the time course data in Fig. 1C of the original publication extends only to 120 min, so it is unclear how the authors were able to show GSH data at 180 min here.
- Page 7, the authors write "Again, our simulated data is consistent with the measured [GSH] time-series in the T98 cell line, with a ~50 min time lag before GSH concentration significantly declines upon glucose deprivation". Looking at Figs. 2B and 2C, I think there is a discrepancy between the simulation of 0 mM glucose Fig. 2B (no time lag, linear decrease in GSH from 3 mM to 0 mM) and the experimental data Fig. 2C (~50 min time lag before GSH begins to decrease). Can the authors explain this discrepancy?
- In Fig. 2D, the authors show a simulation of ROS levels where there is a very rapid transition from low ROS to high ROS. However, in the publication to which the authors have compared their model (PMID 31914417, see Fig. 3A), the experimentally measured levels of ROS increased only linearly (i.e., no rapid transition from low to high ROS). Can the authors explain the discrepancy?
- In Fig. 4, the authors are using very small concentrations of glucose (e.g., 6.3 uM). Is the glucose being depleted over the time course of these experiments? For example, does the emergence of the responsive cells in Fig. 4C at 30-40 min simply reflect that 6.3 uM glucose has been depleted to 0 uM? In Fig. 4H, the cell death measurements are taken at 6 h. For very low concentrations like 1.5-6.3 uM, has the glucose been fully depleted at 6 h? The dose response curves in Fig. 4E and 4H could simply reflect the differing amount of time for the glucose concentration to reach zero.
- In the methods, the authors write: "To ensure its glucose concentration was lower than 1 μM, small molecules in the dFBS were filter-diluted at least 125-fold (5-fold x 3) using an Amicon Ultra-15 centrifugal filter." Did the authors measure the glucose after filter dilution to confirm that the glucose concentration was zero? This seems a key detail since the authors do experiments with very low concentrations of glucose (e.g., 6.3 uM).
- Page 10 and Fig. 4F: The drug GKT137381 (Setanaxib) is described as being used "to inhibit RTK", but this drug is actually an NADPH oxidase 1/4 (NOX1/NOX4) inhibitor. Can the authors please clarify? If the drug is blocking NOX, that would reduce NADPH consumption, potentially explaining the observed results.
- In Fig. 6D, the authors make a very interesting prediction that lowering the cystine concentration will move the bifurcation point between high GSH/low ROS and low GSH/high ROS leftward to lower glucose concentrations. They do not, however, experimentally validate this finding. Could the authors show that cystine dilution affects the percentage of responsive/non-responsive or reversible/irreversible cells in the glucose deprivation / addback experiments that are done in Fig. 5? I believe that would support the prediction of "leftward moving" bifurcation point from Fig. 6.
- Page 13, the authors write "Both cystine dilution alone and co-dilution of cystine and glutamine stretched the GSH bifurcation point leftward (Fig. 6D)." Unless I have misunderstood Fig. 6D, the left

panel (cystine dilution) but *NOT* the right panel (co-dilution of cystine and glutamine) stretches the bifurcation point leftward. Was this a typo, or can the authors clarify?
- A key result from several of the glucose deprivation papers cited by the authors is that knockdown of SLC7A11 levels and/or chemical inhibition of SLC7A11 activity can rescue from glucose deprivation-induced cell death in the presence of cystine and glutamine. Can the authors' model also replicate this experimental finding?
- The authors clearly show that NADPH is the critical metabolite governing redox catastrophe, and this is experimentally supported by some of the cited papers. It would be interesting to see the breakdown of NADPH consumption after glucose deprivation. That is, what percentage of NADPH is consumed by cystine reduction? What percentage is consumed by GSSG reduction? Or other pathways?
- It appears that the authors have done all of their experiments in DMEM. In contrast to DMEM, the commonly used media RPMI contains reduced glutathione (1 mg/L). Can the authors model and/or discuss how addition of reduced glutathione to cell culture media would affect their findings?
- The authors list model assumptions that they have made on page 18, but I think they have overlooked one. Namely, cysteine can be synthesized de novo. I believe that most tumor cells prefer to scavenge cystine and reduce it to cysteine, so I think the authors are justified in making this assumption, but they should mention it in the discussion / model explanation.
- Have the authors made their ODE model and custom MATLAB scripts used for imaging available to community? This is essential for reproducibility.

Minor concerns:
- Fig. 3C, the chosen colors for 0 and 1 steady states are very similar. A different color scheme would make it easier to differentiate these values.
- In Fig. 4E, what time point is being used for ROS measurements?
- Fig. 5H and 5I, at what time point is the percentage of responsive/non-responsive or reversible/irreversible cells at different glucose concentrations calculated?
- In Fig. 6D, the chosen colors for cystine or cystine/glutamine dilution are very similar. A different color scheme would make it easier to differentiate these values.
- RPMI 1640 usually contains 11.11 mM glucose (2 g/mL) not 10 mM as listed on page 15. Table EV1 also lists the glucose concentration in RPMI as 10 mM.
- End of page 3, the authors write "Downstream metabolites of both glucose and glutamine can regenerate NADPH through enzymes (ME1 and IDH1) in the TCA cycle." Technically, ME1 is not part of the TCA cycle but rather adjacent to the TCA cycle.

Reviewer #2:

This manuscript describes a computational model of ROS production by metabolic activity and extracellular nutrients, which is coupled to a single-cell experimental analysis using a live-cell redox reporter. The model is shown to be bistable with respect to extracellular glucose, such that the GSH available to buffer ROS fails suddenly, allowing a rapid rise in ROS. The experimental analysis with an ROS biosensor supports this model, demonstrating a sudden rise in ROS following glucose reduction. The effects of changing extracellular cystine and glutamine concentrations are also explored, revealing a protective effect of cystine depletion. These are potentially important findings, given the importance of ROS in cellular physiology. The text and figures presented are in general very clear and technically sound. I found the paper quite interesting and it would be of interest to a broad audience. However, there are a number of points where the analysis is not fully formed to the point of justifying the intended conclusions (points below). Furthermore, while the systems regulating ROS are nicely introduced, there is essentially no context provided on the state of

quantitative models for ROS regulation, which would greatly enhance the readability of the paper.

1. The weakest part of the paper is the analysis of RTK and calcium effects on ROS. These are interesting regulators of the ROS equilibrium, and it makes sense that they should be included in the model. However, the experiments supporting their effect are minimal and poorly controlled. I am confused why GKT137831 is presented as a perturbation of RTK signaling. This compound is a NOX1/4 inhibitor which has only affects on RTK signaling indirectly. This is an unsatisfactory way to test the involvement of RTK signaling, as many direct inhibitors of tyrosine kinases and phosphatases are available. If the authors intend to include this connection in the manuscript, it would be important to examine such compounds, and to verify their effect on RTK signaling with immunoblots or equivalent assays for the appropriate pathways. Similarly, calcium is perturbed by a single drug, with no validation of how the drug is affecting intracellular calcium concentrations.

2. The analysis of cystine and glutamine dilution in Fig. 6 is interesting and leads to one of the most impressive results of the paper, which is the extension in viability achieved by cystine depletion. However, it is puzzling that the reporter is not used here to examine the predicted changes in ROS. I think this analysis is needed to confirm that the model predictions are correct, and to help better understand the relationship between ROS changes and cell death.

3. An alternative explanation of the all-or-none ROS reporter responses is that the reporter itself is ultrasensitive to ROS concentration under the conditions used. Could the reporter be saturated by low concentrations of ROS, leading to the appearance of bistability in many cells? Can additional evidence be provided that the reporter itself is not generating the sharp responses? Are there conditions under which the biosensor response is not bistable (as predicted by the model, Fig. 3C)?

4. It seems surprising that the sudden transition to high ROS and depletion of GSH doesn't result essentially immediately in cell death. What is happening to the cell in the 3+ hours (Fig. 4I) between the ROS crisis and cell death? How is the cell surviving?

5. A number of other studies have developed computational models of ROS and their interaction with metabolic pathways and signaling. However, the introduction lacks a review of previous work in this area that would help to put the current work in perspective. A paragraph or two making this connection is very much needed in my opinion.

6. The persuasiveness of the experimental analysis would be greatly increased if some of the main concepts could be tested in one or more additional cell lines. Could more justification be provided for the T98 cells that are used?

7. (minor) A few of the plots lack an indication of the experimental variation, which makes it difficult to interpret the significance of the effects shown, including Fig. 4G and 5H/I.

8. (minor) Fig. 5F has an extra text box.

Reviewer #3:

The present manuscript investigates the very important question of the interplay between redox metabolism and signaling. The approach is based on differential equation modeling and measurements of cell survival/ROS across different external glucose. Unfortunately, enthusiasm

was limited by no direct measurements of most key modeled metabolites (even though such measurements are readily experimentally achievable), lack of modeling of glycogen which is a key determinant of the cellular response to glucose removal, and focus on non-physiological glucose concentrations.

Other points:
*Some equations are MM and others mass action. Not clear enough why.
*NADPH equations involve hyperbolic terms of unclear biochemical origin
*Authors mis-report modeling outcomes like facts/experimental observations

We very much appreciate the positive assessments of our manuscript from the first two reviewers and thank all three reviewers for their insightful comments. Not only have we now strengthened our work by incorporating new experimental data that further support the main conclusions of our work, but we have also re-drafted the manuscript to address the points made by the reviewers.

Our revised manuscript comprises the following major modifications:

- We provide an updated figure panel (Figure 4E-4G) showing single-cell ROS dynamics (4F) and percentage of response/nonresponsive cells (4G) under calcium and NOX inhibition.

- We present a new figure panel (Figure 6D-6F) showing single-cell ROS dynamics under three different nutrient modulations.

- We illustrate ROS-response curves (new figure panels 6L-6N) and cell death curves (new figure panel 6O) under three different nutrient modulations.

- We show the ROS-response curves under different titrations of glucose in the U87-MG cell line (new Figure EV3).

- We present validation of calcium and NOX inhibitors (new Figure EV4).

We provide point-by-point responses to the reviewers' comments below.

Reviewer #1:

The manuscript by Huang et al. "Multistability maintains redox homeostasis in human cells" presents a nutrient-redox model that seeks to understand the system-level interplay between nutrient metabolic pathways (glucose, glutamine, cystine) and redox homeostasis. Using in silico modeling and experimental measurements, the authors show that ROS dynamics follow a switch-like, bistable, hysteretic response to glucose deprivation. This manuscript builds on several recent publications demonstrating the role of cystine and the cystine/glutamate antiporter SLC7A11 in regulating cell death following glucose starvation. The paper is well written, and the results from the model nicely synthesize experimental findings from several groups on the role of SLC7A11, cystine, and glutamine in glucose addiction. This work fits

squarely within the scope of MSB. Given the recent interest in cystine metabolism and its relevance to glucose starvation in cancer cells, this work will be a great interest to the cancer and redox metabolism communities, particularly the finding that bistability is a key mechanism required for redox homeostasis. However, there are several issues which the authors should address to improve the manuscript:

Major concerns:

- In Fig. 2C, the authors show a time course of NADPH and GSH levels where "Experimental measurements were adopted from (Joly et al, 2020)". In Fig. 3C of the original publication (PMID 31914417), the levels of NADPH were not detected at times greater than 30 min. It is therefore unclear how the authors here were able to show NADPH data at time points >30 min. In addition, the time course data in Fig. 1C of the original publication extends only to 120 min, so it is unclear how the authors were able to show GSH data at 180 min here.

We apologize for the confusion and thank reviewer #1 for raising this question. For the kinetics of GSH in the T98 cell line upon glucose deprivation shown in original Fig. 2C, the data points were taken from two different sources in the original publication (PMID 31914417). First, fold changes in GSH during the first 120 mins after glucose deprivation were taken from Table S4, which were also plotted in Fig. 3C of that publication. Second, the fold change in GSH 180 mins after glucose deprivation were taken from Table S1 (not shown in Fig. 3C of that publication).

The level of NADPH after 60 min of glucose deprivation had declined to lower than detection limits, as stated in the legend of Fig. 3C in PMID 31914417: "*NADPH dropping below the lower limit of detection within 60 min*". To avoid confusion, we have now labeled these undetected data points with dashed circles in Figure 2C of our revised manuscript and have corrected our figure legend accordingly (page 41 line 998-1000).

- Page 7, the authors write "Again, our simulated data is consistent with the measured [GSH] time-series in the T98 cell line, with a ~50 min time lag before GSH concentration significantly declines upon glucose deprivation". Looking at Figs. 2B and 2C, I think there is a discrepancy between the simulation of 0 mM glucose Fig. 2B (no time lag, linear decrease in GSH from 3 mM to 0 mM) and the experimental data Fig. 2C (~50 min time lag before GSH begins to decrease). Can the authors explain this discrepancy?

We thank reviewer #1 for raising this concern, which prompted us to clarify how we interpreted our simulation results. We acknowledge the discrepancy between our experimental data and simulations. Several factors may contribute to this discrepancy. First, the delays in NADPH and GSH decline in the T98 cell line may be due to compensatory mechanisms, such as glycogen

storage, that sustain NADPH and GSH production upon glucose withdrawal (Yang *et al*, 2015). Second, our model uses rate constants estimated from multiple independent resources of different cell lines, so it may not capture precisely the kinetics of NADPH and GSH specific to the T98 cell line. Despite this discrepancy, we wish to emphasize that our model approximates the real system reasonably well and without relying on exact measurements of the comparative cell line. In particular, distinct separation of the timescales of NADPH and GSH kinetics (fast and slow, respectively) upon glucose deprivation is apparent for both our simulations and experimental results. We have now revised the statement in the Results section noted by the reviewer (page 8-9 line 157-181).

- In Fig. 2D, the authors show a simulation of ROS levels where there is a very rapid transition from low ROS to high ROS. However, in the publication to which the authors have compared their model (PMID 31914417, see Fig. 3A), the experimentally measured levels of ROS increased only linearly (i.e., no rapid transition from low to high ROS). Can the authors explain the discrepancy?

We thank reviewer #1 for raising this important point. In Fig. 3A of PMID 31914417, the investigators used DCF-DA (an irreversible fluorescent indicator for ROS) to measure mean fluorescence intensities. Their experimental method reports population-level ROS. In a scenario where ROS kinetics are highly heterogeneous among individual cells (see our Fig. 4F), population-level measurements can easily mask true single-cell ROS dynamics. As observed in our single-cell ROS quantification in Fig. 4F, the time for ROS increase in individual cells varied from 15 to 75 minutes after glucose starvation. In addition, ~20 % of non-responsive cells maintained low levels of ROS during the first 90 min of glucose starvation (see our Fig. 4D & 4F). Hence, the linear increase in DCF-DA signal in the other publication may simply indicate a gradual increase in the percentage of responsive cells undergoing the switch-like ROS transition from 0 to 90 min. To test that possibility, we calculated mean HyPer7 signals from individual cells in Fig. 4F (shown below) and found that our averaged ROS kinetics resembled the gradually changing population-level changes in ROS of the Graham group (PMID 31914417). Therefore, the discrepancy in ROS measurements may be attributable to the heterogeneity of ROS kinetics among individual cells. Importantly, it is essential to conduct single-cell measurements to reveal: 1) switch-like ROS increases (Fig. 4F); and 2) ultra-sensitivity of ROS to glucose deprivation (Fig. 4E). Both of these important observations support redox multi-stability. We have now included respective statements on page 11 line 229-232 and page 11-12 line 241-245 in our revised manuscript to address this point.

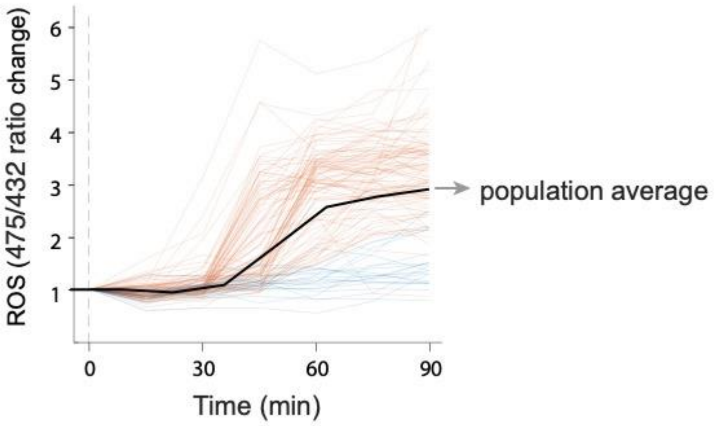

- In Fig. 4, the authors are using very small concentrations of glucose (e.g., 6.3 µM). Is the glucose being depleted over the time course of these experiments? For example, does the emergence of the responsive cells in Fig. 4C at 30-40 min simply reflect that 6.3 µM glucose has been depleted to 0 µM? In Fig. 4H, the cell death measurements are taken at 6 h. For very low concentrations like 1.5-6.3 µM, has the glucose been fully depleted at 6 h? The dose response curves in Fig. 4E and 4H could simply reflect the differing amount of time for the glucose concentration to reach zero.

We appreciate the concern of reviewer #1 regarding our experimental set-up with low glucose. We think that it is not glucose consumption driving our dose-response curves for two reasons. First, if cells consume glucose at a constant rate, a linear ROS dose-response curve would be expected. Instead, we observed a sigmoidal ROS response (Fig. 4E), suggesting ultrasensitivity of the human redox system to glucose starvation. Second, to maintain relatively constant glucose levels, we kept the cell density low in all of our experiments (9000 cells in 200 µL of culture media). Under this condition, it is likely that cells can only consume a small percentage of glucose (< 3%, see calculation below) in a timescale of 60 min.

<u>**Estimation of glucose consumption in our experimental setting:**</u>

Using a glucose uptake rate (see Table EV4, rows #19 - 26) and Km for uptake (Table EV3, row #2) published by others, we calculated:

Total glucose in the media -

$$6.3 \times 10^{-6}\ (mol \cdot L^{-1})\ \times\ 200 \times 10^{-6}\ (L^{-1}) =\ 1260 \times 10^{-12}\ (mol)$$

Rate of glucose consumption -

Since LN18 cells are considered rapid glucose consumers, we used the maximal estimated glucose uptake rate from a pan-cancer survey of 60 different cell lines (Jain et al. 2012):

$900\ fmol \cdot cell^{-1} \cdot hr^{-1}$ in Table EV4 row #19. In the unit of minutes, the rate is $15 \times 10^{-15}\ mol \cdot cell^{-1} \cdot min^{-1}$.

If we measure uptake in well-fed cells, i.e., uptake is close to saturation and $[Glucose] = 6.3\ \mu M$, we can scale down the rate by the Michaelis-Menten term $\frac{[Glucose]}{K_M + [Glucose]}$, where K_M is $1500\ \mu M$, so that the scaling factor is $\frac{6.3}{1500+6.3} \approx 0.004$. Hence, the rate of glucose uptake at $6.3\ \mu M$ glucose is $60 \times 10^{-18}\ mol \cdot cell^{-1} \cdot min^{-1}$. Assuming $9000\ cells$, the overall rate is $540 \times 10^{-15}\ mol \cdot min^{-1}$.

The percentage of glucose consumed in 60 min –

The fraction of glucose consumed is $\frac{540 \times 10^{-15}\ (mol \cdot min^{-1}) \times 60\ (min)}{1260 \times 10^{-12}\ (mol)} \approx 0.025$. Thus, less than 3% glucose is consumed after 60 min when most cells display increasing ROS.

Notes:

1. In these calculations, glucose consumption is assumed to scale linearly with time, whereas it should be exponential (from the Michaelis-Menten term where glucose uptake decelerates), hence our calculation represents a lower estimate of depletion time.
2. The time necessary to fully deplete $1260 \times 10^{-12}\ (mol)$ glucose is $\frac{1260 \times 10^{-12}\ (mol)}{540 \times 10^{-15}\ (mol \cdot min^{-1})} \approx 39\ hr$.
3. The measurements from the literature collated in Table EV4 vary widely due to variable experimental conditions/approaches. The lower and upper estimates of glucose consumption differ almost 100-fold. The estimate we present above assumes a high rate of glucose consumption, but it is still ~5-fold lower than the maximal value in Table EV4. Using that maximal value, the time to depletion would still be $> 7.5\ hr$.
4. It takes surprisingly long for $9000\ cells$ to use up $6.3\ \mu M$ glucose in $200\ \mu L$ media (our experimental condition), even for the fastest glucose-consuming cancer cell lines. This is because glucose uptake is inefficient in the μM range because of its K_M.

- In the methods, the authors write: "To ensure its glucose concentration was lower than 1 µM, small molecules in the dFBS were filter-diluted at least 125-fold (5-fold x 3) using an Amicon Ultra-15 centrifugal filter." Did the authors measure the glucose after filter dilution to confirm that the glucose concentration was zero? This seems a key detail since the authors do experiments with very low concentrations of glucose (e.g., 6.3 µM).

Commercially purchased dFBS usually contains ~ 1 to 5 mg/liter glucose. Whereas commercial dFBS is directly used in most glucose starvation studies, we further conducted dFBS filter-dilution to ensure a low glucose concentration in our experiments. Accordingly, the glucose

concentration in our “zero glucose” experiments is estimated to be less than 3 nM (see calculation below).

dFBS glucose estimation:
The dialyzed FBS we purchased contains ~ 1 mg/liter (5.56 µM) of glucose. We further filter-diluted the glucose concentration five-fold using a 1000 MWC filter three times, resulting in ~ 44.44 nM of glucose. In our experimental design of 5% dFBS, the final glucose concentration in the “zero glucose” group is ~ 2.22 nM.

- Page 10 and Fig. 4F: The drug GKT137381 (Setanaxib) is described as being used "to inhibit RTK", but this drug is actually an NADPH oxidase 1/4 (NOX1/NOX4) inhibitor. Can the authors please clarify? If the drug is blocking NOX, that would reduce NADPH consumption, potentially explaining the observed results.

We thank reviewer #1 for raising this point. NADPH oxidase (NOX) activity is known to contribute to activation of tyrosine kinase (TK) signaling, which further amplifies ROS during glucose deprivation via a feedback mechanism (PMID: L22735335, now stated in our revised Results section, page 5 line 86-88). For clarity, we have now corrected TK signaling feedback to NOX signaling feedback throughout the revised manuscript.

In addition, we validated the effects of calcium and NOX inhibition on tyrosine kinase signaling by probing for phospho-tyrosine kinase by means of Western blot (now added to our revised manuscript as Fig. EV4) and found that treatment with calcium (BAPTA-AM*) and NOX (GKT137831) inhibitors diminished TK phosphorylation by 85.3% and 21.5%, respectively. Dual inhibition of calcium and NOX activity resulted in complete abolition of phospho-tyrosine kinase signaling upon glucose deprivation.

* We switched the calcium inhibitor from Nifedipine to BAPTA-AM given the broader usage and stronger potency of this latter.

Notably, the levels of phospho-tyrosine kinase signaling quantitatively reflect ROS dynamics during glucose deprivation, with suppression of either calcium or NOX signaling compromising the switch-like behavior in ROS and increasing the percentage of non-responsive cells (NOX inhibition: from 19.1% to 27.5%; calcium inhibition: from 19.1% to 80.7%, Fig. 4G). Furthermore, the percentage of non-responsive cells is higher (93.6%) when both signaling pathways are suppressed. We have now included these results on page 12 line 256-266 and page 13 line 272-276 of our revised manuscript.

We agree with reviewer #1 that there is a possibility that a reduction in NADPH consumption after NOX inhibition could contribute to suppression of the switch-like increase in ROS. In the context of the NOX-RTK feedback loop, suppression of NOX can lead to a reduction of: 1) NADPH consumption by NOX; 2) RTK activation; and 3) ROS production by NOX. As indicated by reviewer #1, a reduction in NADPH consumption via NOX inhibition could at least partly contribute to our results presented in Fig. 4F. However, to determine exactly how each of the three aforementioned mechanisms contribute to suppression of ROS and cell death would entail detailed quantitative analyses of the NOX-RTK feedback loop, which we feel is beyond the scope of the current study.

- In Fig. 6D, the authors make a very interesting prediction that lowering the cystine concentration will move the bifurcation point between high GSH/low ROS and low GSH/high ROS leftward to lower glucose concentrations. They do not, however, experimentally validate this finding. Could the authors show that cystine dilution affects the percentage of responsive/non-responsive or reversible/irreversible cells in the glucose deprivation / addback experiments that are done in Fig. 5? I believe that would support the prediction of "leftward moving" bifurcation point from Fig. 6.

We appreciate reviewer #1's enthusiasm for this supposition. We have now experimentally tested the leftward shift in the bifurcation point by quantifying the ROS curve in response to glucose deprivation (similar to Fig. 4E), with additional titrations of cysteine and cysteine/glutamine. As expected, the ROS response curves for both experiments shifted leftward, with a much more pronounced shift for cysteine titration alone. These results are now presented in Fig. 6L-6O and are described on page 17 line 365-375 of the revised manuscript.

*- Page 13, the authors write "Both cystine dilution alone and co-dilution of cystine and glutamine stretched the GSH bifurcation point leftward (Fig. 6D)." Unless I have misunderstood Fig. 6D, the left panel (cystine dilution) but *NOT* the right panel (co-dilution of cystine and glutamine) stretches the bifurcation point leftward. Was this a typo, or can the authors clarify?*

We apologize for the confusion. In our simulations, both dilution of cystine alone (Fig. 6J) and cystine and glutamine co-dilution (Fig. 6K) stretched the bifurcation point leftward. However, only cystine dilution alone stretched the bifurcation point beyond a glucose concentration of zero. To address the reviewer's concern, we have revised our statement on page 17 line 359 and changed the layout of our figure panels (Fig. 6J & 6K) to make our point clearer.

- A key result from several of the glucose deprivation papers cited by the authors is that knockdown of SLC7A11 levels and/or chemical inhibition of SLC7A11 activity can rescue from glucose deprivation-induced cell death in the presence of cystine and glutamine. Can the authors' model also replicate this experimental finding?

We agree with Reviewer #1 that SLC7A11 is a key regulator of glucose deprivation-induced cell death. Given recent interest in SLC7A11 and the centrality of cystine-glutamate interaction in our model, we investigated if our model encompasses the function of SLC7A11. Our results are presented in Fig. EV6A, showing that low SLC7A11 levels may lead to high levels of NADPH and GSH being maintained and low ROS during glucose deprivation. At high SLC7A11 levels, redox catastrophe can occur, reflected in a rapid collapse of GSH and NADPH levels and a concomitant swift increase in ROS. These simulation results are consistent with previous studies (Joly *et al*, 2020; Koppula *et al*, 2017; Liu *et al*, 2020; Shin *et al*, 2017) showing that SLCA11 knockdown may rescue cells from glucose deprivation-induced cell death.

- The authors clearly show that NADPH is the critical metabolite governing redox catastrophe, and this is experimentally supported by some of the cited papers. It would be interesting to see the breakdown of NADPH consumption after glucose deprivation. That is, what percentage of NADPH is consumed by cystine reduction? What percentage is consumed by GSSG reduction? Or other pathways?

Reviewer #1 has raised an important aspect of redox homeostasis, i.e. NADPH budgeting. We can estimate NADPH allocation based on steady-state metabolite concentrations using our model. In fact, this is how we estimated reaction rate constants and allocated unknown fluxes (Table EV6, EV8, and EV9). In Table EV8, we estimated NADPH allocation under the fed condition. That same approach can also be applied to glucose-deprived conditions. Please note that we estimated NADPH allocation using the glucose-addicted parameter set, i.e. the same parameter set used throughout our paper.

Table 1. Simulated steady-state concentrations of NADPH, cystine, and GSSG under glucose-fed (10000 μM glucose) and glucose-deprived (0 μM glucose) conditions

Steady-state concentration	Glucose-fed (μM)	Glucose-deprived (μM)
[NADPH]	0.88	0.031
[Cystine]	6.5	180
[GSSG]	100	1500

Table 2. Simulated NADPH allocation under glucose-fed and glucose-deprived conditions

Reaction	NADPH allocation (glucose-fed)		NADPH allocation (glucose-deprived)	
	Flux (µM / min)	Percentage used (%)	Flux (µM / min)	Percentage used (%)
Cystine reduction	170	16.7	170	62
GSSG reduction	150	14.7	79	29
Anabolism	700	68.6	25	9

The numbers reported in these tables have been rounded to two significant digits. We note that the allocation of NADPH to cystine reduction in terms of flux is the same for glucose-fed and glucose deprived conditions. However, the allocations to GSSG reduction and anabolism are greatly reduced under glucose-deprived conditions, explaining the inflated percentage allocation to cystine reduction under those conditions. Without explicitly imposing a hierarchy for NADPH allocation, our model recapitulates the toxicology of cystine under glucose-deprived conditions, monopolising the limited supply of NADPH. One interpretation of the reduced NADPH allocation to anabolism is that cells would not be able to replace damaged macromolecules adequately (e.g., lipids) due to NADPH insufficiency, leading to an accumulation of oxidative damage.

- It appears that the authors have done all of their experiments in DMEM. In contrast to DMEM, the commonly used media RPMI contains reduced glutathione (1 mg/L). Can the authors model and/or discuss how addition of reduced glutathione to cell culture media would affect their findings?

We acknowledge the possibility that using different media for cell culture may affect experimental results. We postulate that addition of GSH to the media would delay glucose deprivation-induced ROS elevation and cell death.

Previous studies have shown that GSH displays low cellular permeability (Levy *et al*, 1993), which limits its direct cellular import. Most cell types, other than kidney and intestinal epithelial cells, lack direct GSH uptake mechanisms (Deneke *et al*, 1995). One mechanism by which cells can utilize extracellular GSH is via membrane-bound γ-glutamyl-transferase (GGT) (Schafer *et al*, 2001), which facilitates breakdown of extracellular GSH into its constituent amino acids (glycine, glutamate, cysteine), followed by cysteine uptake by the neutral amino acid transporter ASCT for *de novo* GSH synthesis in cells (Zhang *et al*, 2005). Thus, this pathway represents an alternative mechanism for synthesizing GSH, with the advantage of bypassing NADPH consumption for cystine reduction into bioactive cysteine. Therefore, we reason that addition of GSH would delay ROS elevation and cell death.

- The authors list model assumptions that they have made on page 18, but I think they have overlooked one. Namely, cysteine can be synthesized de novo. I believe that most tumor cells prefer to scavenge cystine and reduce it to cysteine, so I think the authors are justified in making this assumption, but they should mention it in the discussion / model explanation.

We thank reviewer #1 for this suggestion. Indeed, we built our model under the assumption that *de novo* cysteine synthesis via the trans-sulfuration pathway plays a relatively minor role in glucose-addicted cancer cells displaying high xCT expression. This assumption is based on previous studies demonstrating that cell lines having high expression levels of xCT lack or exhibit low expression of cystathionine b-synthase (CBS), an enzyme crucial to the trans-sulfuration pathway (Zhu *et al*, 2019). We have now modified our list of assumptions accordingly and cite this reference on page 21 line 467-469.

- Have the authors made their ODE model and custom MATLAB scripts used for imaging available to community? This is essential for reproducibility.

Yes. Our ODE model is available at https://github.com/imb-lcd/2021_redoxmodel.

Minor concerns:
- Fig. 3C, the chosen colors for 0 and 1 steady states are very similar. A different color scheme would make it easier to differentiate these values.

Acknowledged. We have adjusted the colors in our revised manuscript.

- In Fig. 4E, what time point is being used for ROS measurements?

The time-point is 1.5 hr after glucose deprivation. We have now included this information in our figure legend.

- Fig. 5H and 5I, at what time point is the percentage of responsive/non-responsive or reversible/irreversible cells at different glucose concentrations calculated?

For Fig. 5H, the time-point is 75 min after glucose starvation. For Fig. 5I, the time-point is 75 min after adding back glucose. We have now included this information in our figure legend.

- In Fig. 6D, the chosen colors for cystine or cystine/glutamine dilution are very similar. A different color scheme would make it easier to differentiate these values.

Acknowledged. We have adjusted these colors in our revised manuscript.

- RPMI 1640 usually contains 11.11 mM glucose (2 g/mL) not 10 mM as listed on page 15. Table EV1 also lists the glucose concentration in RPMI as 10 mM.

Apologies. We have now corrected the glucose concentration of RPMI to 11.11 mM in our revised manuscript.

- End of page 3, the authors write "Downstream metabolites of both glucose and glutamine can regenerate NADPH through enzymes (ME1 and IDH1) in the TCA cycle." Technically, ME1 is not part of the TCA cycle but rather adjacent to the TCA cycle.

Acknowledged. We have now modified our description on page 4 line 61 of our revised manuscript.

Reviewer #2:

This manuscript describes a computational model of ROS production by metabolic activity and extracellular nutrients, which is coupled to a single-cell experimental analysis using a live-cell redox reporter. The model is shown to be bistable with respect to extracellular glucose, such that the GSH available to buffer ROS fails suddenly, allowing a rapid rise in ROS. The experimental analysis with an ROS biosensor supports this model, demonstrating a sudden rise in ROS following glucose reduction. The effects of changing extracellular cystine and glutamine concentrations are also explored, revealing a protective effect of cystine depletion. These are potentially important findings, given the importance of ROS in cellular physiology. The text and figures presented are in general very clear and technically sound. I found the paper quite interesting and it would be of interest to a broad audience. However, there are a number of points where the analysis is not fully formed to the point of justifying the intended conclusions (points below). Furthermore, while the systems regulating ROS are nicely introduced, there is essentially no context provided on the state of quantitative models for ROS regulation, which would greatly enhance the readability of the paper.

We thank reviewer #2 for this valuable suggestion. We have now included a paragraph in our revised Introduction describing previous efforts to mathematically model the human redox system (page 5 line 93-103).

1. The weakest part of the paper is the analysis of RTK and calcium effects on ROS. These are interesting regulators of the ROS equilibrium, and it makes sense that they should be included in the model. However, the experiments supporting their effect are minimal and poorly controlled. I am confused why GKT137831 is presented as a perturbation of RTK signaling. This compound is a NOX1/4 inhibitor which has only affects on RTK signaling indirectly. This is an unsatisfactory way to test the involvement of RTK signaling, as many direct inhibitors of tyrosine kinases and phosphatases are available. If the authors intend to include this connection in the manuscript, it would be important to examine such compounds, and to verify their effect on RTK signaling with immunoblots or equivalent assays for the appropriate pathways. Similarly, calcium is perturbed by a single drug, with no validation of how the drug is affecting intracellular calcium concentrations.

We thank reviewer #2 for raising this point.
NADPH oxidase (NOX) activity is known to contribute to activation of tyrosine kinase (TK) signaling, which further amplifies ROS during glucose deprivation via a feedback mechanism (PMID: L22735335, now stated in our revised Results section, page 5 line 86-88). For clarity, we have now corrected TK signaling feedback to NOX signaling feedback throughout the revised manuscript.

In addition, we validated the effects of calcium and NOX inhibition on tyrosine kinase signaling by probing for phospho-tyrosine kinase by means of Western blot (now added to our revised manuscript as Fig. EV4) and found that treatment with calcium (BAPTA-AM*) and NOX (GKT137831) inhibitors diminished TK phosphorylation by 85.3% and 21.5%, respectively. Dual inhibition of calcium and NOX activity resulted in complete abolition of phospho-tyrosine kinase signaling upon glucose deprivation.

* We switched the calcium inhibitor from Nifedipine to BAPTA-AM given the broader usage and stronger potency of this latter.

Notably, the levels of phospho-tyrosine kinase signaling quantitatively reflects ROS dynamics during glucose deprivation, with suppression of either calcium or NOX signaling compromising the switch-like behavior in ROS and increasing the percentage of non-responsive cells (NOX inhibition: from 19.1% to 27.5%; calcium inhibition: from 19.1% to 80.7%, Fig. 4G). Furthermore, the percentage of non-responsive cells is higher (93.6%) when both signaling pathways are suppressed. We have now included these results on page 12 line 256-266 and page 13 line 272-276 of our revised manuscript.

2. The analysis of cystine and glutamine dilution in Fig. 6 is interesting and leads to one of the most impressive results of the paper, which is the extension in viability achieved by cystine depletion. However, it is puzzling that the reporter is not used here to examine the predicted changes in ROS. I think this analysis is needed to confirm that the model predictions are correct, and to help better understand the relationship between ROS changes and cell death.

We appreciate reviewer #1's enthusiasm for this supposition. We have now experimentally tested (1) the ROS dynamics and (2) the leftward shift in the bifurcation point by quantifying the ROS-response curve in response to glucose deprivation (similar to Fig. 4E), with additional titrations of cysteine and cysteine/glutamine. As expected, the ROS responses for both experiments are suppressed (Fig. 6D-6F) and ROS-response curves shifted leftward (Fig. 6L-6O), with a much more pronounced respective suppression and shift for cysteine titration alone. These results are now presented in Fig. 6D-6F & 6L-6O and are described on page 15 line 332-342, and page 17 line 365-375 of the revised manuscript.

3. An alternative explanation of the all-or-none ROS reporter responses is that the reporter itself is ultrasensitive to ROS concentration under the conditions used. Could the reporter be saturated by low concentrations of ROS, leading to the appearance of bistability in many cells? Can additional evidence be provided that the reporter itself is not generating the sharp responses? Are there conditions under which the biosensor response is not bistable (as predicted by the model, Fig. 3C)?

As mentioned by reviewer #2, the ROS kinetics reported by the HyPer7 reporter mostly showed switch-like elevations upon glucose deprivation (left panel, Fig. 4F). Two lines of evidence suggest that this swift increase in ROS signal is not due to inherent properties of Hyper7 nor of its saturation. First, the original Hyper7 study clearly demonstrates its graded response to H_2O_2 in the nanomolar range (Fig. 3A of (Pak *et al*, 2020). Second, we show that when cells were treated with inhibitors of NOX and calcium, most ROS responses (> 90%) were transformed from being sharp and switch-like to graded responses (middle and right panels, Fig. 4F).

4. It seems surprising that the sudden transition to high ROS and depletion of GSH doesn't result essentially immediately in cell death. What is happening to the cell in the 3+ hours (Fig. 4I) between the ROS crisis and cell death? How is the cell surviving?

We thank reviewer #2 for this interesting question that is central to the mechanism underlying oxidative cell death, yet it remains an open question in the field. We suspect that ROS, most likely H_2O_2 (a relatively low-reactive species) as the predominant form, may take some time to

cause lethal and irreversible cell damage.

5. A number of other studies have developed computational models of ROS and their interaction with metabolic pathways and signaling. However, the introduction lacks a review of previous work in this area that would help to put the current work in perspective. A paragraph or two making this connection is very much needed in my opinion.

We thank reviewer #2 for this suggestion. As mentioned previously, we have now included a paragraph in our revised Introduction describing previous efforts to mathematically model the human redox system (page 5 line 93-103).

6. The persuasiveness of the experimental analysis would be greatly increased if some of the main concepts could be tested in one or more additional cell lines. Could more justification be provided for the T98 cells that are used?

We now include quantification of the ultrasensitivity of the ROS-response curve in another glucose-addicted cell line (U87-MG) in Fig. EV3, with a respective statement in page 12 line 252-255.

7. (minor) A few of the plots lack an indication of the experimental variation, which makes it difficult to interpret the significance of the effects shown, including Fig. 4G and 5H/I.

Apologies. We now include experimental descriptions on page 42 line 1034 and page 44 line 1058-1059 in our revised manuscript.

8. (minor) Fig. 5F has an extra text box.

Apologies, now deleted.

Reviewer #3:

The present manuscript investigates the very important question of the interplay between redox metabolism and signaling. The approach is based on differential equation modeling and measurements of cell survival/ROS across different external glucose. Unfortunately, enthusiasm was limited by no direct measurements of most key modeled metabolites (even though such measurements are readily experimentally achievable), lack of modeling of glycogen which is a key determinant of the cellular response to glucose removal, and focus on non-physiological glucose concentrations.

We thank Reviewer #3 for recognizing the importance of the question that we investigated and

for highlighting the importance of glycogen in the glucose starvation response.

In response to "*lack of modeling of glycogen*":

We are keenly interested in understanding the potential cytoprotective roles of glycogen in cancer cells and, like Reviewer #3, we regard glycogen as one of the potential key determinants of the immediate cellular response to glucose deprivation. We did in fact consider modeling glycogen as an approach to tackling this problem. However, due to the severe paucity of quantitative studies on glycogen, the bottom-up modeling approach we used in this study could not be applied to glycogen because there are so few quantitative measurements of glycogen across different cell lines/types and the rates at which glycogen is utilized in both fed and starved cells. To our knowledge, there is only one published study from the past decade that directly assessed by molecular genetics the role of glycogen metabolism in glucose starvation (Yang et al. 2015) and, regrettably, glycogen content has not received much attention from the field. Lee et al. (2018(Lee *et al*, 2018) showed that intracellular glucose content is correlated with the response of cell lines to glucose starvation. Although intracellular glucose may reflect glycogen content, it remains unclear if it represents a faithful measure of the glycogen reservoir. Moreover, glycogen deposits and the way in which they are supplied can vary widely across cell lines, thus representing an additional layer of variability. In striving for a realistic model of nutrient-redox metabolism, we look forward to developing quantitative assays providing single-cell glycogen measurements, enabling us to extend the model presented in the current manuscript. Nevertheless, the major insights pertaining to redox bistability derived from our model and our supporting experiments represent a strong foundation for characterizing redox homeostasis, despite our limited understanding of glycogen. Redox bistability describes the steady-state behavior of a system, whereas regulation of the glycogen reservoir most likely only affects the system's kinetics but not its steady state. We anticipate that once glycogen is depleted, the redox system will follow the same steady-state trajectory shown by our study.

In response to "no direct measurements of most key modeled metabolites":

We agree with Reviewer #3 that key variables should be measured in order to obtain a comprehensive understanding of redox metabolism, when technologies permit. In fact, Dr. Nicholas Graham's group at University of Southern California has recently published a comprehensive metabolomic and phenotypic study encompassing several glucose-addicted glioblastoma cell lines (Joly *et al.*, 2020), including the LN18 cell line we employed in our experimental set-up. In Joly et al. (2020), the Graham group showed that the LN18 cell line closely resembles the T98 cell line in terms of its metabolomic and phenotypic dynamics upon glucose deprivation. They also resolved the metabolome dynamics of the T98 cell line over a 3-hour time-course—including NADPH and GSH, i.e., the two central redox metabolites in our study—and our simulations are in general agreement with their findings. Importantly, our use of the H_2O_2 sensor Hyper7, a newly developed reversible sensor for quantitative and steady-state measurements, resolves one key variable that metabolomics could not determine, i.e., ROS (the effectors of cell damage), providing quantitative data at single-cell resolution. Our

study greatly complements the work of the Graham group, and *vice versa*, together providing a comprehensive overview of the dynamics of the redox metabolome.

In response to "focus on non-physiological glucose concentrations":

We understand Reviewer #3's concern regarding the "non-physiological" concentration of glucose. We wish to emphasize the scientific and applied value of perturbing glucose concentrations to the low micromolar range, in particular for understanding its regulatory impact on redox homeostasis and its potential application in anticancer therapeutics.

Studies in the past two decades of low glucose perturbations have revealed it functions in maintaining redox homeostasis. Those studies range from simple cytological observations to explorations of underlying molecular mechanisms and system-level syntheses. Together, they offer a mechanistic overview of the glucose addiction of many cancers from the perspective of cellular redox homeostasis. In the late 1990s, Dr. Spitz's group showed that ROS-mediated oxidative stress, not energy stress, accounts for low glucose (< 10 μM)-induced cell death in a multidrug-resistant but glucose-dependent cell line (Lee *et al*, 1998). Subsequently, this lethal elevation in ROS was shown to be caused by a NOX-ROS-TK signaling feedback loop in glucose-addicted cell lines (Graham *et al*, 2012). Consistently, it has been shown that prioritizing NADPH usage for antioxidant defense over anabolic reactions via AMPK signaling enables cell survival in the wake of glucose withdrawal, leading to the discovery that AMPK displays a previously unappreciated function in metabolic reprogramming (Jeon *et al*, 2012). More recently, gene expression profiling (Koppula *et al.*, 2017), genetic screening (Shin *et al.*, 2017), and metabolite screening (Goji *et al*, 2017), all in the background of severe glucose restriction, identified SLC7A11 as a key regulator and cystine uptake as an early event in glucose deprivation-induced cell death, thereby delineating the upstream element of this distinct cell death pathway. Those studies have revealed intricate functional coordination between glucose and other nutrients in redox metabolism, which have profound but long overlooked implications for cancer metabolism. Moreover, they have exposed deregulation of redox metabolism in glucose-addicted cell lines as a targetable vulnerability that may be exploited by cancer therapeutics (Joly *et al.*, 2020; Liu *et al.*, 2020). The aim of our study was to extend that body of work, providing a systems-level understanding of the human redox system during low glucose stress. We have built the first nutrient-redox model, predicted a key mechanism for redox homeostasis (i.e., bistability), and experimentally demonstrated bistability using quantitative single-cell measurements of ROS.

In a broader context, glucose has been found to exert a critical role in maintaining redox homeostasis during the extreme glucose restrictions that can occur under various pathological conditions, such as in the tumor microenvironment or during hypoglycemia-induced brain failure (Chang *et al*, 2015; Cryer, 2007; Navratilova *et al*, 2013). Importantly, without low-level glucose perturbations, none of those findings would have been possible. Finally, in terms of methodology, the findings of all aforementioned studies on glucose display parallels with research on another nutrient, i.e., cysteine. Non-physiological levels of cystine have been shown to induce a newly identified form of regulated cell death, ferroptosis, which plays a

significant role in various pathological conditions, including cancers and neurodegenerative diseases (Jiang *et al*, 2021).

To emphasize the functional role of glucose in maintaining redox homeostasis, especially at low glucose concentrations, we have modified the Introduction of our revised manuscript (page 5-6 line 104-117).

Other points:
**Some equations are MM and others mass action. Not clear enough why.*

We thank Reviewer #3 for pointing this out. We now include a section in our revised Methods (page 27 line 583-591) to explain our choice of Michaelis-Menten (hyperbolic) and mass action terms in our model.
"Generally, we assume reactions to follow mass action without *a priori* knowledge, with the exceptions of nutrient uptake and ROS autoinhibition. For nutrient uptake, the glucose, glutamine, and cystine-glutamate transporters are known to be saturated by nutrient concentrations in the culture media (Table EV3) and are therefore modeled by Michaelis-Menten kinetics. Note that glutamine uptake and glutamate production are considered together, and glucose uptake is likewise considered with the NADPH and ROS production terms."

**NADPH equations involve hyperbolic terms of unclear biochemical origin*

Apologies. We have now included a section in our revised Methods (page 27 line 583-591, detailed above) to explain our choice of hyperbolic terms for the NADPH equations in our model.

**Authors mis-report modeling outcomes like facts/experimental observations*

Apologies, we have now revised how we describe our modeling results to more clearly distinguish them from experimental results (page 8 line 157, page 8 line 172, page 9 line 182, and page 16 line 344 & line 358 of the revised manuscript).

References:

Chang CH, Qiu J, O'Sullivan D, Buck MD, Noguchi T, Curtis JD, Chen QY, Gindin M, Gubin MM, van der Windt GJW *et al* (2015) Metabolic Competition in the Tumor Microenvironment Is a Driver of Cancer Progression. *Cell* 162: 1229-1241
Cryer PE (2007) Hypoglycemia, functional brain failure, and brain death. *J Clin Invest* 117: 868-870
Deneke SM, Susanto I, Vogel KA, Williams CE, Lawrence RA (1995) Mechanisms of Use of Extracellular Glutathione by Lung Epithelial-Cells and Pulmonary-Artery Endothelial-Cells. *Am J Resp Cell Mol* 12: 662-668

Goji T, Takahara K, Negishi M, Katoh H (2017) Cystine uptake through the cystine/glutamate antiporter xCT triggers glioblastoma cell death under glucose deprivation. *Journal of Biological Chemistry* 292: 19721-19732
Graham NA, Tahmasian M, Kohli B, Komisopoulou E, Zhu MG, Vivanco I, Teitell MA, Wu H, Ribas A, Lo RS *et al* (2012) Glucose deprivation activates a metabolic and signaling amplification loop leading to cell death. *Molecular Systems Biology* 8
Jeon SM, Chandel NS, Hay N (2012) AMPK regulates NADPH homeostasis to promote tumour cell survival during energy stress. *Nature* 485: 661-+
Jiang XJ, Stockwell BR, Conrad M (2021) Ferroptosis: mechanisms, biology and role in disease. *Nat Rev Mol Cell Bio* 22: 266-282
Joly JH, Delfarah A, Phung PS, Parrish S, Graham NA (2020) A synthetic lethal drug combination mimics glucose deprivation-induced cancer cell death in the presence of glucose. *Journal of Biological Chemistry* 295: 1350-1365
Koppula P, Zhang YL, Shi JJ, Li W, Gan BY (2017) The glutamate/cystine antiporter SLC7A11/xCT enhances cancer cell dependency on glucose by exporting glutamate. *Journal of Biological Chemistry* 292: 14240-14249
Lee HY, Itahana Y, Schuechner S, Fukuda M, Je HS, Ogris E, Virshup DM, Itahana K (2018) Ca2+-dependent demethylation of phosphatase PP2Ac promotes glucose deprivation-induced cell death independently of inhibiting glycolysis. *Science Signaling* 11
Lee YJ, Galoforo SS, Berns CM, Chen JC, Davis BH, Sim JE, Corry PM, Spitz DR (1998) Glucose deprivation-induced cytotoxicity and alterations in mitogen-activated protein kinase activation are mediated by oxidative stress in multidrug-resistant human breast carcinoma cells. *Journal of Biological Chemistry* 273: 5294-5299
Levy EJ, Anderson ME, Meister A (1993) Transport of Glutathione Diethyl Ester into Human-Cells. *P Natl Acad Sci USA* 90: 9171-9175
Liu X, Olszewski K, Zhang Y, Lim EW, Shi J, Zhang X, Zhang J, Lee H, Koppula P, Lei G *et al* (2020) Cystine transporter regulation of pentose phosphate pathway dependency and disulfide stress exposes a targetable metabolic vulnerability in cancer. *Nat Cell Biol* 22: 476-486
Navratilova J, Hankeova T, Benes P, Smarda J (2013) Low-glucose conditions of tumor microenvironment enhance cytotoxicity of tetrathiomolybdate to neuroblastoma cells. *Nutr Cancer* 65: 702-710
Pak VV, Ezerina D, Lyublinskaya OG, Pedre B, Tyurin-Kuzmin PA, Mishina NM, Thauvin M, Young D, Wahni K, Gache SAM *et al* (2020) Ultrasensitive Genetically Encoded Indicator for Hydrogen Peroxide Identifies Roles for the Oxidant in Cell Migration and Mitochondrial Function. *Cell Metab* 31: 642-+
Schafer C, Fels C, Brucke M, Holzhausen HJ, Bahn H, Wellman M, Visvikis A, Fischer P, Rainov NG (2001) Gamma-glutamyl transferase expression in higher-grade astrocytic glioma. *Acta Oncol* 40: 529-535
Shin CS, Mishra P, Watrous JD, Carelli V, D'Aurelio M, Jain M, Chan DC (2017) The glutamate/cystine xCT antiporter antagonizes glutamine metabolism and reduces nutrient flexibility. *Nature Communications* 8
Yang RQ, Zhang M, Gustafson AR, Wang E, Cole MP, Tooley CES, Cheng A (2015) Loss of protein targeting to glycogen sensitizes human hepatocellular carcinoma cells towards glucose deprivation mediated oxidative stress and cell death. *Bioscience Reports* 35
Zhang HQ, Forman HJ, Choi J (2005) gamma-Glutamyl transpeptidase in glutathione biosynthesis. *Method Enzymol* 401: 468-483
Zhu JJ, Berisa M, Schworer S, Qin WG, Cross JR, Thompson CB (2019) Transsulfuration Activity Can Support Cell Growth upon Extracellular Cysteine Limitation. *Cell Metab* 30: 865-+

Thank you for sending us your revised manuscript. We have now heard back from the two reviewers who agreed to evaluate your manuscript. You will see from the comments below that both reviewers are satisfied with the modifications made and think that the points raised by all three reviewers have been adequately dealt with and the study is now suitable for publication in Molecular Systems Biology.

Before we can formally accept your manuscript, we would ask you to address the following editorial-level issues.

REFEREE REPORTS

Reviewer #1:

The authors have sufficiently addressed all of my previous concerns. In particular, the addition of the new experimental data in Fig. 6 regarding the effects of cystine and cystine/glutamine dilution confirms some of the results from their model. I think this is a high quality study combining model and experiments that will be of broad interest to the cancer metabolism/redox community.

I reviewed the comments from and responses to Reviewer #3, and in short, I do believe they have appropriately addressed all concerns.

Reviewer #2:

In this revised manuscript, the authors have thoroughly responded to the large majority of the points raised. This study adds significantly to the existing body of literature on redox regulation by presenting an integrated model of multiple processes, which are well validated by elegant experiments with single cell resolution.

Responses after revision

Reviewer #1:

The authors have sufficiently addressed all of my previous concerns. In particular, the addition of the new experimental data in Fig. 6 regarding the effects of cystine and cystine/glutamine dilution confirms some of the results from their model. I think this is a high quality study combining model and experiments that will be of broad interest to the cancer metabolism/redox community.

I reviewed the comments from and responses to Reviewer #3, and in short, I do believe they have appropriately addressed all concerns.

Reviewer #2:

In this revised manuscript, the authors have thoroughly responded to the large majority of the points raised. This study adds significantly to the existing body of literature on redox regulation by presenting an integrated model of multiple processes, which are well validated by elegant experiments with single cell resolution.

2nd Revision - Editorial Decision 10th Sep 2021

Thank you for sending us your revised manuscript. Our data editors have seen the manuscript, and they have made some comments and suggestions that need to be addressed (see attached). Please send back a revised version (in track change mode), as we will need to go through the changes. In addition, please address the following editorial-level issues.

3rd Authors' Response to Reviewers 11th Sep 2021

The authors have made all requested editorial changes.

ACCEPTED **14th Sep 2021**

Thank you again for sending us your revised manuscript. We are now satisfied with the modifications made and I am pleased to inform you that your paper has been accepted for publication.

EMBO PRESS

YOU MUST COMPLETE ALL CELLS WITH A PINK BACKGROUND

PLEASE NOTE THAT THIS CHECKLIST WILL BE PUBLISHED ALONGSIDE YOUR PAPER

Corresponding Author Name: Sheng-hong Chen
Journal Submitted to: Molecular Systems Biology
Manuscript Number: MSB-2021-10480RR

Reporting Checklist For Life Sciences Articles (Rev. June 2017)

This checklist is used to ensure good reporting standards and to improve the reproducibility of published results. These guidelines are consistent with the Principles and Guidelines for Reporting Preclinical Research issued by the NIH in 2014. Please follow the journal's authorship guidelines in preparing your manuscript.

A- Figures
1. Data
The data shown in figures should satisfy the following conditions:

- the data were obtained and processed according to the field's best practice and are presented to reflect the results of the experiments in an accurate and unbiased manner.
- figure panels include only data points, measurements or observations that can be compared to each other in a scientifically meaningful way.
- graphs include clearly labeled error bars for independent experiments and sample sizes. Unless justified, error bars should not be shown for technical replicates.
- if n< 5, the individual data points from each experiment should be plotted and any statistical test employed should be justified
- Source Data should be included to report the data underlying graphs. Please follow the guidelines set out in the author ship guidelines on Data Presentation.

2. Captions
Each figure caption should contain the following information, for each panel where they are relevant:

- a specification of the experimental system investigated (eg cell line, species name).
- the assay(s) and method(s) used to carry out the reported observations and measurements
- an explicit mention of the biological and chemical entity(ies) that are being measured.
- an explicit mention of the biological and chemical entity(ies) that are altered/varied/perturbed in a controlled manner.
- the exact sample size (n) for each experimental group/condition, given as a number, not a range;
- a description of the sample collection allowing the reader to understand whether the samples represent technical or biological replicates (including how many animals, litters, cultures, etc.).
- a statement of how many times the experiment shown was independently replicated in the laboratory.
- definitions of statistical methods and measures:
  - common tests, such as t-test (please specify whether paired vs. unpaired), simple χ2 tests, Wilcoxon and Mann-Whitney tests, can be unambiguously identified by name only, but more complex techniques should be described in the methods section;
  - are tests one-sided or two-sided?
  - are there adjustments for multiple comparisons?
  - exact statistical test results, e.g., P values = x but not P values < x;
  - definition of 'center values' as median or average;
  - definition of error bars as s.d. or s.e.m.

Any descriptions too long for the figure legend should be included in the methods section and/or with the source data.

In the pink boxes below, please ensure that the answers to the following questions are reported in the manuscript itself. Every question should be answered. If the question is not relevant to your research, please write NA (non applicable). We encourage you to include a specific subsection in the methods section for statistics, reagents, animal models and human subjects.

USEFUL LINKS FOR COMPLETING THIS FORM

http://www.antibodypedia.com
http://1degreebio.org
http://www.equator-network.org/reporting-guidelines/improving-bioscience-research-repor
http://grants.nih.gov/grants/olaw/olaw.htm
http://www.mrc.ac.uk/Ourresearch/Ethicsresearchguidance/Useofanimals/index.htm
http://ClinicalTrials.gov
http://www.consort-statement.org
http://www.consort-statement.org/checklists/view/32-consort/66-title
http://www.equator-network.org/reporting-guidelines/reporting-recommendations-for-tumo
http://datadryad.org
http://figshare.com
http://www.ncbi.nlm.nih.gov/gap
http://www.ebi.ac.uk/ega
http://biomodels.net/
http://biomodels.net/miriam/
http://jjj.biochem.sun.ac.za
https://osp.od.nih.gov/biosafety-biosecurity-and-emerging-biotechnology/
http://www.selectagents.gov/

B- Statistics and general methods

Please fill out these boxes (Do not worry if you cannot see all your text once you press return)

1.a. How was the sample size chosen to ensure adequate power to detect a pre-specified effect size?	In general, the sample size of single-cell measurements is equal or bigger than 100 cells, which captures the quantitative properties (mean and variation) of population response.
1.b. For animal studies, include a statement about sample size estimate even if no statistical methods were used.	N.A.
2. Describe inclusion/exclusion criteria if samples or animals were excluded from the analysis. Were the criteria pre-established?	Non-responsive cells were excluded for the estimation of ROS ultrasensitivity (Fig. 4E & Fig. S1) due to their distinct regulation of redox state. The non-responsive cells are defined by their ROS fold change (< 2 fold) within 90 min of glucose deprivation as stated in our manuscript.
3. Were any steps taken to minimize the effects of subjective bias when allocating animals/samples to treatment (e.g. randomization procedure)? If yes, please describe.	In general, three experimental repeats were carried out and each experimental repeat has technical repeats (n > 3). The order of imaging is randomized through the imaging software control.
For animal studies, include a statement about randomization even if no randomization was used.	N.A.
4.a. Were any steps taken to minimize the effects of subjective bias during group allocation or/and when assessing results (e.g. blinding of the investigator)? If yes please describe.	In general, three experimental repeats were carried out and each experimental repeat has technical repeats (n > 3). The order of imaging is randomized through the imaging software control.
4.b. For animal studies, include a statement about blinding even if no blinding was done	N.A.
5. For every figure, are statistical tests justified as appropriate?	Nonparametric Mann-Whitney U-test was used in Fig. 4I without assuming a specific distribution of data.
Do the data meet the assumptions of the tests (e.g., normal distribution)? Describe any methods used to assess it.	Nonparametric Mann-Whitney U-test was used in Fig. 4I without assuming a specific distribution of data.
Is there an estimate of variation within each group of data?	N.A.

Is the variance similar between the groups that are being statistically compared?	N.A.

C- Reagents

6. To show that antibodies were profiled for use in the system under study (assay and species), provide a citation, catalog number and/or clone number, supplementary information or reference to an antibody validation profile. e.g., Antibodypedia (see link list at top right), 1DegreeBio (see link list at top right).	Phospho-Tyrosine (4G10) Mouse mAb (Cat. No. 96215) was purchased from Cell Signaling Technology.
7. Identify the source of cell lines and report if they were recently authenticated (e.g., by STR profiling) and tested for mycoplasma contamination.	Human brain glioblastoma LN18 cells were purchased from ATCC (CRL-2610). U87-MG parental cells (BCRB No. 60360), human brain glioblastoma cell-line, were purchased from Bioresource Collection and Research Center (BCRC) in Taiwan. Polymerase chain reaction confirmed the LN18 and U87-MG parentals as well as their ROS reporters (LN18-HyPer7, and U87-MG-HyPer7) cells to be mycoplasma-free.

* for all hyperlinks, please see the table at the top right of the document

D- Animal Models

8. Report species, strain, gender, age of animals and genetic modification status where applicable. Please detail housing and husbandry conditions and the source of animals.	N.A.
9. For experiments involving live vertebrates, include a statement of compliance with ethical regulations and identify the committee(s) approving the experiments.	N.A.
10. We recommend consulting the ARRIVE guidelines (see link list at top right) (PLoS Biol. 8(6), e1000412, 2010) to ensure that other relevant aspects of animal studies are adequately reported. See author guidelines, under 'Reporting Guidelines'. See also: NIH (see link list at top right) and MRC (see link list at top right) recommendations. Please confirm compliance.	N.A.

E- Human Subjects

11. Identify the committee(s) approving the study protocol.	N.A.
12. Include a statement confirming that informed consent was obtained from all subjects and that the experiments conformed to the principles set out in the WMA Declaration of Helsinki and the Department of Health and Human Services Belmont Report.	N.A.
13. For publication of patient photos, include a statement confirming that consent to publish was obtained.	N.A.
14. Report any restrictions on the availability (and/or on the use) of human data or samples.	N.A.
15. Report the clinical trial registration number (at ClinicalTrials.gov or equivalent), where applicable.	N.A.
16. For phase II and III randomized controlled trials, please refer to the CONSORT flow diagram (see link list at top right) and submit the CONSORT checklist (see link list at top right) with your submission. See author guidelines, under 'Reporting Guidelines'. Please confirm you have submitted this list.	N.A.
17. For tumor marker prognostic studies, we recommend that you follow the REMARK reporting guidelines (see link list at top right). See author guidelines, under 'Reporting Guidelines'. Please confirm you have followed these guidelines.	N.A.

F- Data Accessibility

18: Provide a "Data Availability" section at the end of the Materials & Methods, listing the accession codes for data generated in this study and deposited in a public database (e.g. RNA-Seq data: Gene Expression Omnibus GSE39462, Proteomics data: PRIDE PXD000208 etc.) Please refer to our author guidelines for 'Data Deposition'.<br><br>Data deposition in a public repository is mandatory for:<br>a. Protein, DNA and RNA sequences<br>b. Macromolecular structures<br>c. Crystallographic data for small molecules<br>d. Functional genomics data<br>e. Proteomics and molecular interactions	N.A.
19. Deposition is strongly recommended for any datasets that are central and integral to the study; please consider the journal's data policy. If no structured public repository exists for a given data type, we encourage the provision of datasets in the manuscript as a Supplementary Document (see author guidelines under 'Expanded View' or in unstructured repositories such as Dryad (see link list at top right) or Figshare (see link list at top right).	N.A.
20. Access to human clinical and genomic datasets should be provided with as few restrictions as possible while respecting ethical obligations to the patients and relevant medical and legal issues. If practically possible and compatible with the individual consent agreement used in the study, such data should be deposited in one of the major public access-controlled repositories such as dbGAP (see link list at top right) or EGA (see link list at top right).	N.A.
21. Computational models that are central and integral to a study should be shared without restrictions and provided in a machine-readable form. The relevant accession numbers or links should be provided. When possible, standardized format (SBML, CellML) should be used instead of scripts (e.g. MATLAB). Authors are strongly encouraged to follow the MIRIAM guidelines (see link list at top right) and deposit their model in a public database such as Biomodels (see link list at top right) or JWS Online (see link list at top right). If computer source code is provided with the paper, it should be deposited in a public repository or included in supplementary information.	Mathematic code for our nutrient-redox model is available at https://github.com/imb-lcd/2021_redoxmodel.

G- Dual use research of concern

22. Could your study fall under dual use research restrictions? Please check biosecurity documents (see link list at top right) and list of select agents and toxins (APHIS/CDC) (see link list at top right). According to our biosecurity guidelines, provide a statement only if it could.	N.A.
